# A dose escalation study to evaluate the safety of an aerosol BCG infection in previously BCG-vaccinated healthy human UK adults

**DOI:** 10.3389/fimmu.2024.1427371

**Published:** 2024-11-14

**Authors:** Timothy Fredsgaard-Jones, Stephanie A. Harris, Hazel Morrison, Alberta Ateere, Beatrice Nassanga, Raquel Lopez Ramon, Celia Mitton, Eve Fletcher, Jonathan Decker, Hannah Preston-Jones, Susan Jackson, Andrew Mawer, Iman Satti, Michael Barer, Timothy Hinks, Henry Bettinson, Helen McShane

**Affiliations:** ^1^ The Jenner Institute, University of Oxford, Oxford, United Kingdom; ^2^ Department of Respiratory Science, University of Leicester, Leicester, United Kingdom; ^3^ Oxford Centre for Respiratory Medicine, Nuffield Department of Clinical Medicine, University of Oxford, Oxford, United Kingdom

**Keywords:** tuberculosis, BCG, CHIM, vaccine, aerosol

## Abstract

**Introduction:**

Tuberculosis (TB) is the leading cause of death worldwide from a single infectious agent. Bacillus Calmette-Guérin (BCG), the only licensed vaccine, provides limited protection. Controlled human infection models (CHIMs) are useful in accelerating vaccine development for pathogens with no correlates of protection; however, the need for prolonged treatment makes *Mycobacterium tuberculosis* an unethical challenge agent. Aerosolised BCG provides a potential safe surrogate of infection. A CHIM in BCG-vaccinated as well as BCG-naïve individuals would allow identification of novel BCG-booster vaccine candidates and facilitate CHIM studies in populations with high TB endemicity. The purpose of this study was to evaluate the safety and utility of an aerosol BCG CHIM in historically BCG-vaccinated volunteers.

**Methods:**

There were 12 healthy, historically BCG-vaccinated UK adults sequentially enrolled into dose-escalating groups. The first three received 1 × 10^4^ CFU aerosol BCG Danish 1331 via a nebuliser. After safety review, subsequent groups received doses of 1 × 10^5^ CFU, 1 × 10^6^ CFU, or 1 × 10^7^ CFU. Safety was monitored through self-reported adverse events (AEs), laboratory tests, and lung function testing. Immunology blood samples were taken pre-infection and at multiple timepoints post-infection. A bronchoalveolar lavage (BAL) taken 14 days post-infection was analysed for presence of live BCG.

**Results:**

No serious AEs occurred during the study. Solicited systemic and respiratory AEs were frequent in all groups, but generally short-lived and mild in severity. There was a trend for more reported AEs in the highest-dose group. No live BCG was detected in BAL from any volunteers. Aerosol BCG induced potent systemic cellular immune responses in the highest-dose group 7 days post-infection.

**Discussion:**

Aerosol BCG infection up to a dose of 1 × 10^7^ CFU was well-tolerated in historically BCG-vaccinated healthy, UK adults. No live BCG was detected in the BAL fluid 14 days post-infection despite potent systemic responses, suggesting early clearance. Further work is needed to expand the number of volunteers receiving BCG via the aerosol route to refine and establish utility of this aerosol BCG CHIM.

**Clinical trial registration:**

https://clinicaltrials.gov/, identifier NCT04777721.

## Introduction

Globally, tuberculosis (TB) remains the leading cause of death from a single infectious agent (1), and even in survivors, it exerts a significant impact on the quality of their health (2). The WHO has set ambitious targets to curb the impact of TB, with an aim to reduce mortality to 95% of the numbers seen in 2015 (3). Integral to the success of this strategy will be the development and deployment of a more efficacious vaccine than BCG (3, 4), which provides limited protection to *Mycobacterium tuberculosis* (*M.tb*) infection in adults and has variable efficacy against pulmonary disease (5). To date, the lack of defined correlates of protection and a robust model for assessing efficacy and immunogenicity for candidate vaccines has frustrated vaccine development (6). There is an urgent need for other tools to identify at an early stage which vaccine candidates have the greatest chance of success. Controlled human infection models (CHIMs) have been successfully utilised in the development of vaccine strategies for other globally important diseases including malaria, influenza, cholera, and typhoid (7–10). CHIMs also provide the opportunity to interrogate the immunological responses to early infection (11), something which is challenging in *M.tb* field studies where infection often predates symptoms by months or years. A functioning TB CHIM could also be utilised to directly assess the vaccine efficacy of a novel vaccine as part of early-stage clinical trials on healthy volunteers, by providing a head-to-head comparison with unvaccinated controls. Given the high costs of phase 3 trials for TB vaccines (12), this would be invaluable in ensuring that only the most promising candidates are taken forward.

Developing a CHIM for TB vaccines is complex, as the duration and toxicity of treatment as well as lack of a definitive proof of cure mean that virulent *M.tb* would not be an ethical challenge agent. In the absence of an attenuated form of *M.tb*, Bacillus Calmette-Guérin (BCG) provides an attractive alternative challenge agent due to its low pathogenic potential and excellent safety record as a vaccine (13). We have previously developed an intradermal BCG CHIM (14–16); however, using a respiratory CHIM would be beneficial, as it accurately mimics the natural route of *M.tb* infection and allows for the characterisation of local mucosal immune responses.

Instilling BCG directly into the lungs has previously been shown to be safe and well tolerated (17); however, aerosol delivery provides a less invasive approach and logistical and operational benefits in a low-resource setting. We have previously shown that aerosol BCG can be safely administered to BCG-naïve healthy adult volunteers and that viable BCG can be successfully recovered from bronchoalveolar lavage (BAL) samples taken 14 days after initial challenge (18). Ongoing work has focussed on evaluating the local respiratory and systemic responses to aerosol challenge (ClinicalTrials.gov NCT03912207). As the majority of the world’s population are vaccinated with BCG as part of routine childhood vaccine schedules, it is important that any TB CHIM could also be conducted on historically BCG-vaccinated volunteers. Not only would this allow for CHIMs to be conducted on more varied populations including settings where TB is endemic, but also it would allow for the evaluation of future TB vaccine candidates in a prime-boost regimen with intradermal BCG. Prime-boost approaches have been shown to enhance *M.tb* immunity in animal models (19), and heterologous vaccine programs have recently shown promise in providing protection against COVID-19 (20).

Evaluation of the safety of aerosol BCG infection in historically BCG-vaccinated humans is prudent given the large surface area of the respiratory mucosa. Intradermal BCG revaccination has not been associated with an increased side effect profile in large randomised controlled trials (21), whereas studies involving non-human primates are reassuring, showing that an aerosol vaccination boost of historically intradermally vaccinated animals can also be achieved safely (22). The results of another respiratory BCG human challenge model via bronchoscopic instillation included some participants with previous BCG vaccination and also showed a favourable side effect profile (17), albeit at a lower dose to a single lobe of the lung.

Here, we present the results of a dose escalation study investigating the safety and tolerability of aerosol BCG infection in historically BCG-vaccinated healthy adult volunteers from an area with low *M.tb* prevalence. We also present data on BCG recovery from bronchoalveolar lavage fluid and face mask sampling post-infection and exploratory immunological data to characterise the response to aerosol BCG infection, and to further inform the immunological response to mycobacteria.

## Materials and methods

### Study design

We designed a controlled human infection study using BCG delivered via the aerosol route. The study, along with associated documents, was approved by the Oxford A Research Ethics Committee (Ref. 20/SC/0059) and was registered with ClinicalTrials.gov prior to study commencement (ClinicalTrials.gov ID: NCT04777721). All aspects of the study were conducted according to the principle of the Declaration of Helsinki and Good Clinical Practice.

### Trial participants

Healthy, historically BCG-vaccinated UK adults aged 18–50 residing in the Oxford area were recruited by use of approved adverts. Following written informed consent, volunteers were screened for eligibility at the Centre for Clinical Vaccinology and Tropical Medicine, University of Oxford. Volunteers were screened for previous exposure to *M.tb* through clinical history, physical examination, chest radiographs, and a negative interferon gamma release assay (QuantiFERON-TB Gold Plus (Qiagen, Hilden, Germany)) on fresh blood. BCG vaccination status was confirmed via occupational health/medical records or evidence of a BCG scar, whereas to be eligible, previous intradermal BCG vaccination must have occurred at least 12 months prior to enrolment.

Volunteers were screened for evidence of an underlying immunodeficiency, including HIV, through serological and haematological testing and medical history. Volunteers with underlying asthma or other significant respiratory disease evidenced through medical history or clinically significant abnormalities in pulmonary function tests or chest radiograph were excluded. Volunteers who were current smokers, pregnant, or breastfeeding and those who had a medical history, which was deemed by the study physicians to be clinically relevant, were also excluded. The full eligibility criteria can be found in the study protocol (**Supplementary Document 1**). Volunteers who fulfilled the inclusion criteria were invited to participate.

### Group allocation

Volunteers were sequentially enrolled into one of four study groups, starting at the lowest aerosol BCG dose until each study group was full (three volunteers per group). Volunteers were not blinded to their dose. A review of the safety data taken from volunteers was completed at least 7 days after the final volunteer in each group had been enrolled. Safety data assessment included solicited and unsolicited adverse events (AEs) reported in electronic diaries (eDiaries), safety blood tests, pulmonary function tests, bronchoscopy reports, and physical observations from study visits. Data were discussed with an independent safety monitoring committee (SMC). The decision to continue dose escalation was at the discretion of the committee, and only after agreement with the SMC was enrolment to the next dosing group initiated.

### Challenge agent

BCG Danish 1331 (AJV Vaccines, Denmark) was used for all groups. According to the manufacturer, lyophilised vials contain 2–8 × 10^6^ CFU; therefore, the median of 5 × 10^6^ CFU was assumed for dosing. All vials were mixed with BCG solvent (AJV vaccines) and diluted with 0.9% normal saline as appropriate for the dose in each study group. The total volume of fluid aerosolised for each volunteer was 1 mL. Aerosolisation was achieved using the handheld Omron MicroAir U22 ultrasonic mesh nebuliser (OMRON Healthcare Ltd., UK). Volunteers were instructed to breathe at a normal rate and the device set in a continuous aerosolisation mode.

### Clinical interventions

All volunteers received one dose of aerosolised BCG (either 1 × 10^4^, 1 × 10^5^, 1 × 10^6^, or 1 × 10^7^ CFU) on D0 of the study. Blood was obtained on all study visits (D0, D2, D7, D14, D28, D56, D84, and D168) whereas bronchoscopy was completed on D14, and induced sputum was an optional investigation on D168.

After challenge, the volunteers were provided with an eDiary to record solicited respiratory AEs (cough, sputum production, haemoptysis, sore throat, wheeze, dyspnoea, chest tightness, and chest pain) and systemic AEs (fever, feverishness, fatigue, malaise, myalgia, arthralgia, headache, and nausea) for 28 days. Volunteers were also asked to record any other unlisted AEs. All AEs were scored between 0 (not present) and 3 (severe) based on predefined criteria (see **Supplementary Figure 1**). The inclusion of systemic AEs was identified based on SmPC for BCG (23), whereas respiratory AEs were based on previous experience from aerosol vaccine trials including previous trials completed using aerosol BCG (18, 24). AEs (solicited and unsolicited) were also recorded at all study visits where durations of AEs and their severity were collected retrospectively.

Basic vital signs were recorded at all study visits. Spirometry was completed immediately after administration of aerosol BCG on D0 and repeated on D2 and D7 visits, and Transfer Factor of the lung for Carbon Dioxide (TL_CO_) was completed on the D7 visit. Any volunteers with potentially significant drops in TL_CO_ at D7 (≥15% compared with baseline or a TL_CO_ below the predicted lower limit of normal adjusted for age, height, gender, and ethnicity) had repeat measurement undertaken at D84. Further lung function was also repeated where deemed clinically indicated.

Volunteers reporting any grade 3 AEs during eDiary attended for an additional clinic visit. Volunteers with a fever (≥37.5°C) were investigated with physical examination and history and underwent blood tests and microbiological investigation including throat swabs for respiratory pathogens and mycobacterial blood cultures as appropriate.

A single bronchoscopy was completed on D14 following aerosol challenge by an experienced respiratory physician. Volunteers were offered intravenous sedation (midazolam and fentanyl). After local anaesthesia was administered to the oropharynx, above and below the vocal cords, a fibreoptic bronchoscope was used to macroscopically inspect respiratory mucosa and to obtain a bronchoalveolar lavage of the right middle lobe using 150 mL of sterile 0.9% saline.

Face mask sampling was completed using an adapted duckbill face mask (Integrity^®^ 600-3004) containing four 3D-printed polyvinyl alcohol (PVA) sampling matrix strips to detect any exhaled BCG. Masks were worn for 30 min on several study visits: D0, both before and immediately after aerosol challenge, D2, D7, D14, and D28. Masks were positioned to avoid any contact with face and sampling strips. Volunteers were asked to avoid touching the internal components of the masks but were permitted to talk and cough while wearing the masks. Volunteers were not permitted to eat while wearing the mask; however, if they felt the need to expectorate sputum or required a drink during the procedure, they were permitted to briefly remove the mask before replacing. Masks were placed in a sampling bag and allowed to dry before sealing for transport at room temperature.

### Detection and quantification of BCG

#### BCG infection dose

The infection dose was verified by generating serial dilutions of BCG from the diluted vaccine vials and adding the appropriate dilution to a BACTEC™ Mycobacterial Growth Indicator Tube (MGIT) containing Middlebrook 7H9 media, supplemented with 800 µL of BBL MGIT OADC (oleic acid, albumin, dextrose, and catalase) and PANTA (polymyxin B, amphotericin B, nalidixic acid, trimethoprim, and azlocillin) mixture (Becton Dickinson, UK) (BD). Tubes were added to the BACTEC™ MGIT instrument (Becton Dickinson, UK) and time to positivity (TTP) by fluorescence recorded. TTP is inversely correlated with the number of CFU present and was converted to CFU by means of a standard curve, where serial dilutions of a BCG vaccine vial were generated and an aliquot of each dilution plated onto solid M7H11 agar and an aliquot added to a supplemented MGIT tube. Resulting TTP was plotted against log10 CFU and a linear regression performed to obtain an equation for the line. Results are reported as CFU/vaccine vial and CFU/dose for each group.

#### Detection of BCG in BAL and induced sputum samples

BCG detection was performed on the entire BAL sample volume from all 12 volunteers in the study, as previously described (18). Briefly, BAL samples were centrifuged at 3,000 g for 17 min and the supernatant decanted into fresh 50 mL centrifuge tubes and stored at −80°C. The pellet was decontaminated using the BBL^®^ MycoPrep™ Specimen Digestion/Decontamination Kit (Becton Dickinson, UK) according to the manufacturer’s instructions. Briefly, the ampule in the MycoPrep™ Reagent bottle was broken and N-acetyl-L-cysteine (NALC) was dissolved by gentle shaking to activate the NALC-sodium hydroxide (NALC-NaOH) solution. 5 mL was added to the BAL pellet and incubated for 15 min at room temperature with occasional gentle mixing. Phosphate-buffered saline (PBS) was then added to 50 mL, and the solution was centrifuged at 3,000 g for 17 min. Supernatant was discarded and the pellet resuspended in supplemented media from the corresponding MGIT tube and returned to the tube. The MGIT tube was added to the BACTEC™ MGIT instrument (Becton Dickinson, UK), as above, and TTP was recorded.

Induced sputum samples were obtained from 11/12 volunteers at D168 post-aerosol BCG. Induced sputum samples were decontaminated by adding an equal volume of activated BBL^®^ MycoPrep™ following the above procedure for BCG detection in the BAL.

#### Processing of adapted PVA masks

For groups 3 and 4 (1 × 10^6^ and 1 × 10^7^ CFU aerosol BCG), facemasks from D0 pre- and post-aerosol BCG, D2, and D7 were processed for detection of live BCG. Under sterile conditions, the two PVA strips from one side of the face mask were removed and placed into a stomacher bag. The remainder of the mask containing the other two PVA strips was repackaged and stored at RT for later qPCR. 4.5 mL of molecular-grade water was added to the strips in the stomacher bag and the bag manipulated by hand for 5 min to dissolve the strips. Once dissolved, the solution was transferred to a 50 mL Falcon tube and an equal volume of BBL^®^ MycoPrep™ was added and processed as above for detection of BCG. All facemasks from all time points were processed for qPCR.

For qPCR analysis, the remaining two PVA strips were removed from the face mask and dissolved in 4 mL Tris buffer (25 mM, pH 8.0) using a multifunction tube rotator for 60 min. After dissolution, samples were pelleted using centrifugation (15,000 x *g* for 10 min). The pellet was resuspended in 100 µL Tris–EDTA (20 mM Tris, 2 mM EDTA, pH 8.0) before being transferred to a screwcap tube. 0.25 g of lysing matrix B and 100 µL Chelex suspension (50% w/v Chelex 100, 1% w/v Nonidet P-40, 1% w/v Tween 20) were added to the resuspended pellet. Cells were disrupted in a FastPrep-24 5G homogeniser (MP Biomedicals, CA, USA) at 6.5 m/s for 45 s (4 rounds with a 2 min incubation on ice between each round). Samples were centrifuged at 15,000 x *g* for 2 min and the supernatant collected for qPCR analysis. Sample DNA was initially detected using SensiFAST™ Probe No-ROX (Bioline, UK) following the manufacturer’s instructions, with primers and probes targeting the IS1081 gene: forward primer: 5′-CTG CTC TCG ACG TTC ATC GCC C-3′ (final concentration: 333 nM); reverse primer: 5′-GGC TAG CAG ACC TCA CCT ATG TGT-3′ (final concentration: 333 nM); probe: 5′-6-FAM GGC TGA AGC/ZEN/CGA CGC CCT GTG CGG G-3′-IABFQ (final concentration: 167 nM). Thermal cycling conditions for the IS1081 assay was as follows: incubation at 50°C for 2 min, incubation at 95°C for 10 min, followed by 45 PCR cycles (denaturation: 95°C for 15 s; combined annealing/extension: 60°C for 60 s). Positive samples were re-assayed for detection of the IS6110 gene using SensiFAST™ SYBR^®^ No-Rox (Bioline, UK) according to the manufacturer’s instructions. Primers used to target the IS6110 gene: forward primer: 5′-AGC GTA GGC GTC GGT GAC-3′ (final concentration: 400 nM); reverse primer: 5′-GGG TAG CAG ACC TCA CCT ATG TGT-3′ (final concentration: 400 nM) (25). Thermal cycling for the IS6110 assay was performed with the conditions as follows: incubation at 95°C for 15 min, followed by 40 PCR cycles (denaturation: 95°C for 15 s; annealing: 68°C for 30 s; extension: 72°C for 20 s; additional extension step: 82°C for 20 s). All thermal cycling was performed using a Rotor-Gene Q thermocycler (Qiagen, Cat N: 9001590).

#### Genotyping of mycobacteria isolated from BAL and induced sputum

After a minimum of 42 days on the BACTEC™ MGIT instrument (when the manufacturer’s protocol considers the sample negative), the contents of positive MGIT tubes were transferred to a 50 mL centrifuge tube and centrifuged at 3,000 g for 17 min. Supernatant was discarded and the pellet resuspended in 200 µL PBS and stored at −80°C for batched DNA extraction. DNA extraction was carried out as previously described (26). Briefly, DNA was released from 200 µL of thawed homogenate using the tough microorganism lysing kit (Precellys) in a Precellys 24 machine at 6,500 rpm for 3 × 30 s. Homogenate was transferred to a separate tube and 50 µL PBS used to wash the remaining homogenate from the beads. 180 µL of ATL buffer and 20 µL of proteinase K (Qiagen) were added, vortexed, and incubated at 56°C for 4 h. From this point, the extractions were carried out following the manufacturer’s instructions (Qiagen DNeasy Blood and Tissue Kit).

Genotyping was carried out using the HAIN™ GenoType MTBC VER 1.X kit (Bruker) according to the manufacturer’s instructions for use. Briefly, DNA extracted from BAL and induced sputum was amplified using kit-specific primers and PCR conditions. 20 µL of the PCR product was chemically denatured and hybridised to the kit-specific DNA•STRIP, which contains specific probes complementary to the amplified PCR products. The bound amplicon is detected by addition of streptavidin–alkaline phosphatase and made visible by a colorimetric reaction. This results in a specific banding pattern on the strip, corresponding to a particular mycobacterium species, determined by comparing with the kit-specific insert.

### Immunology

#### *E vivo* enzyme-linked immunospot

Fresh peripheral blood mononuclear cells (PBMC) separated from whole blood (WB) were used to measure *ex vivo* IFN-γ enzyme-linked immunospot (ELISpot) responses as previously described on samples collected at D0, D7, D14, D28, D56, D84, and D168 of the study (27). Briefly, cells were stimulated in triplicate at 3 × 10^5^ PBMC/well with 20 µg/mL of PPD from *M.tb* (AJ Vaccines, Denmark), 2 × 10^5^ CFU/mL BCG Danish (AJ Vaccines, Denmark), 1 µg/mL of MTB300, a pool comprising 300 peptides from *M.tb* (Sette, La Jolla), and anti-human CD3 mAb (positive control; Mabtech AB) or left unstimulated as a negative control for the assay. Background (unstimulated) subtracted antigen-specific responses are presented as spot-forming cells (SFC) per 1 × 10^6^ PBMC.

#### Concentration of BAL fluid

Frozen BAL fluid (BALF) was thawed and duplicate 20 µL volumes used to determine the phospholipid concentration by means of a phospholipid assay kit (Sigma-Aldrich). The manufacturer’s instructions were followed and the concentration obtained in µM converted to concentration in µg/mL using the molecular weight of phosphatidyl-choline (28).

The physiological concentration of phospholipid in the lung is estimated to be 1 mg/mL, so it would have been desirable to concentrate BALF to this physiological concentration; however, samples were normalised to 0.5 mg/mL of phospholipid to ensure sufficient volume remained for enzyme-linked immunosorbent assay (ELISA) analysis, while still increasing the likelihood that the antibody concentration would rise above the limit of detection (29). BAL antibody arbitrary units were then multiplied by 2 in order to present BAL antibody per 1 mg/mL phospholipid. BAL samples were concentrated by adding 15 mL volumes to separate Amicon^®^ Ultra-15 Centrifugal Filter Units—10-kDa cutoff (Millipore) and centrifuging at 3,000 g for between 5 min and 30 min, depending on the extent of concentration required. When the desired volume was achieved, the portion of sample remaining above the filter was transferred to a 2 mL cryovial and stored at –80°C for later analysis.

#### Enzyme-linked immunosorbent assay

ELISAs, measuring PPD-specific IgG and IgA, were performed on serum taken at D0 and at days 2, 7, 14, 28, 56, 84, and 168 post-aerosol BCG infection and on concentrated BALF taken at D14. ELISA plates (Nunc-Immuno MaxiSorp) were coated with 50 µL/well of 5 µg/mL PPD in PBS and incubated overnight at 4°C. Plates were washed and blocked with 100 µL/well casein for 1 h at room temperature. Serum samples were diluted 1 in 50 (or repeated with a higher dilution if necessary) in casein and standards prepared by serial 1 in 2 dilutions of a reference pool of serum. Blocking solution was discarded, and 50 µL of samples and standard curve dilutions were added to duplicate wells and plates incubated at room temperature for 2 h. Plates were washed, and 50 µL of a 1 in 1,000 dilution of anti-human goat IgG (y-chain-specific)-alkaline phosphatase antibody (Sigma-Aldrich) or a 1 in 500 dilution of anti-human goat IgA (α-chain-specific)-alkaline phosphatase antibody (Sigma-Aldrich) was added. Plates were incubated for 1 h at room temperature. Plates were washed and developed by adding 50 µL/well of p-nitrophenyl phosphate and disodium salt (pNPP) substrate (Sigma-Aldrich). After 5 min, plates were read at 405 nm using Gen5 software (v2.0.7, BioTek) and read approx. every 30 min to ensure a read where the standards produced a curve with parameters within the specified range. Plates passed if average optical density values for the blank wells were <0.15, the standard curve was within predefined parameters and if the optical density of replicate sample values had a coefficient of variation <20%. BAL antibody values are presented as arbitrary units based on the standard curve and multiplied by 2, based on the phospholipid physiological concentration of 1 mg/mL.

#### Direct PBMC mycobacterial growth inhibition assay

The direct PBMC Mycobacterial growth inhibition assay (MGIA) was carried out on frozen PBMC from all 12 volunteers, collected at D0 and at days 7, 28, and 56 post-aerosol BCG, as previously described (30). Briefly, 3 ×10^6^ PBMC and ~500 CFU BCG Pasteur were combined in a total volume of 600 µL RPMI (containing 25 mM HEPES, 2 mM L-glutamine 20% foetal calf serum, and 1% v/v sodium pyruvate) in a 48-well plate. Plates were incubated at 37°C and 5% CO_2_ for 96 h, and then contents of each well were added to separate 2 mL screw-cap tubes and centrifuged at 12,000 rpm for 10 min. 500 µL sterile water was added to each well and left for a minimum of 5 min to lyse adherent monocytes. Supernatants were carefully removed from the 2 mL tubes and discarded, and water from a corresponding well was added to the pellet. Tubes were vortexed for 1 s and the contents added to BACTEC™ MGIT tubes supplemented as above and added to the BACTEC™ MGIT instrument until TTP was detected. On day 0, duplicate viability control tubes were set up by adding the same volume of BCG Pasteur stock directly to supplemented BACTEC™ MGIT tubes, as was added to each well containing the PBMC sample. TTP readout was converted to log_10_ CFU using a standard curve of TTP against CFU (enumerated by growth on solid M7H11 agar). Results are presented as growth ratio (log10 CFU sample/log10 CFU control).

### Statistical analysis

All statistical analysis was performed using GraphPad Prism (v10). Prism calculates exact p-values, which takes into account tied values. Non-parametric tests were applied as data were not normally distributed. Differences in medians between two groups were calculated using the Mann–Whitney test and the Wilcoxon signed rank test for paired data. Correction for multiple comparisons was done using Friedman test. For comparison of non-continuous safety data, chi-squared tests were used. In all cases, significant differences are presented with their p value; the significance level was set as p < 0.05.

## Results

### Participants

The first volunteer was enrolled into group 1 (1 × 10^4^ CFU) on 06/07/2022; volunteers were sequentially enrolled until each study group was full (three volunteers per group). After a satisfactory SMC outcome was reached, enrolment continued in this way until enrolment into the final group (group 4) was completed on 04/01/2023.

**Figure 1:**
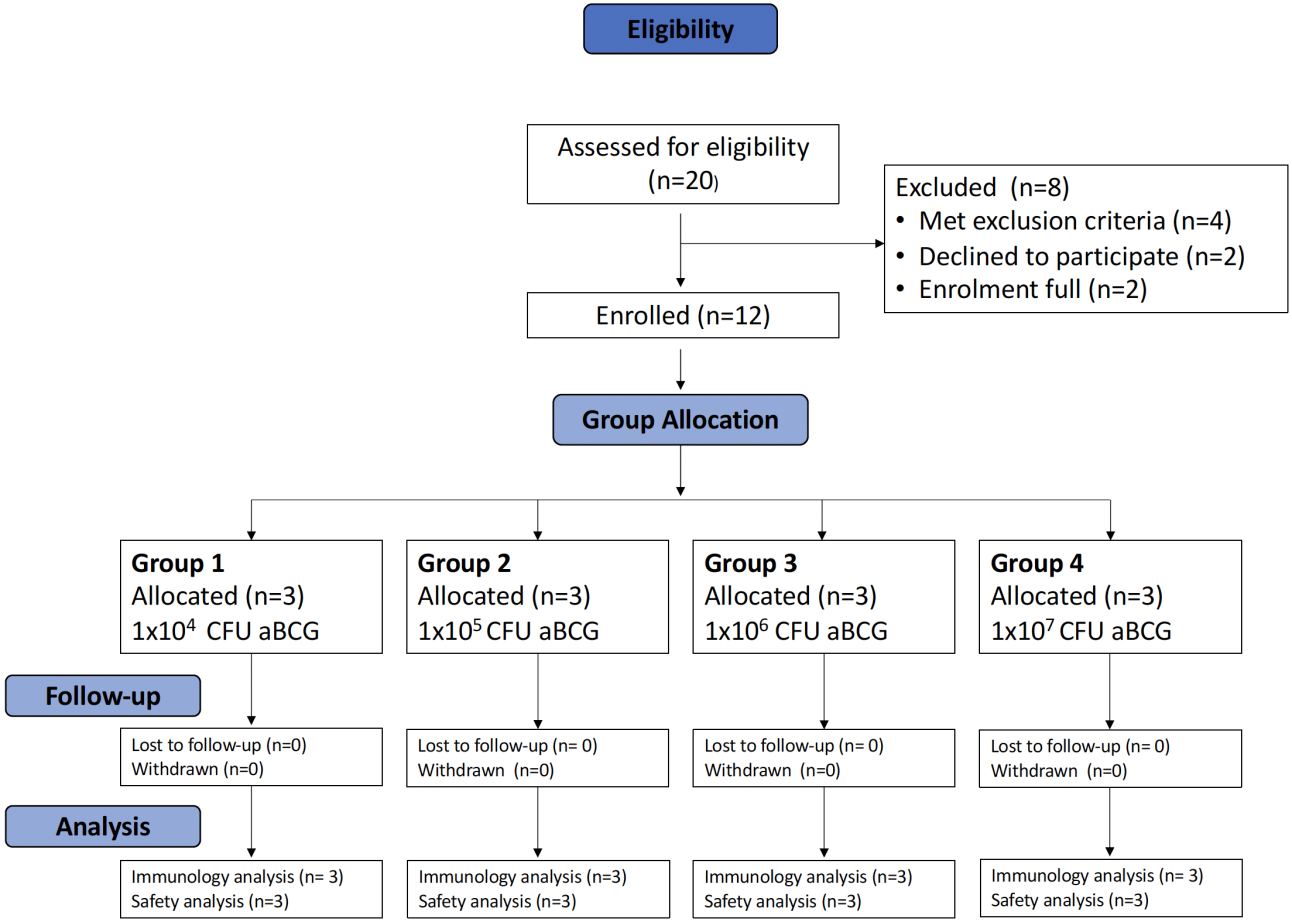
Consort diagram for TB044.

In total, 20 volunteers were assessed for eligibility in the trial, and 12 volunteers were enrolled across 4 groups (**Figure 1**). In total, eight women and four men were enrolled; the median age of the volunteers was 35 years (range 23 to 49). The baseline demographics of the volunteers are displayed in **Table 1**.

**Table 1:** Demographics and baseline respiratory function of all enrolled volunteers.

Characteristic	
**Female, n (%)**	8 (67)
**Median age in years (range)**	35 (23–49)
**BMI (kg/m^2^) median (range)**	26.6 (21.4–35.4)
Country of birth	
**UK**	7
**South Africa**	2
**Chile**	1
**Pakistan**	1
**Portugal**	1
**Time since first BCG vaccination in years, median (range)**	28 (16–37)
Baseline spirometry	
**Mean % predicter FEV1 (range)**	109 (87–124)
**Mean % predicted FVC (range)**	107 (90–123)
**Mean % predicted TL_CO_ (range)**	100 (87–116)
**Mean % predicted K_CO_ (range)**	103 (87–113)
**Median baseline % SaO_2_ (range)**	98 (97–100)

### Safety

No serious adverse events of any kind occurred during the study. All volunteer visits were completed as per the protocol and were performed within the specified window.

Solicited symptoms reported by the volunteers are presented below. Few unsolicited symptoms deemed to be possibly, probably, or definitely related were reported throughout the study. These were generally mild and of short duration and are outlined in **Supplementary Table 1**.

### Solicited adverse events in 14 days following aerosol BCG administration

Solicited symptoms were frequent in all groups, but generally mild in severity. The median duration of AEs first reported within 72 hours of challenge was between 1 and 3 days for all systemic AEs and 2 and 4 days for respiratory AEs (**Table 2**). There were no grade 3 AEs of any type in the first 14 days after challenge.

**Table 2:** Median duration of solicited AEs reported within 72 h of aerosol BCG challenge.

Solicited AE	Number of volunteers (proportion, max grade)				Total n=12	Median duration of AE in days (range)	
	Group 1n=3	Group 2 n=3	Group 3 n=3	Group 4 n=3			
**Temperature**	0	0	0	1 (0.33, 2)	1	2	–
**Arthralgia**	0	0	0	2 (0.66, 2)	2	2.5	(2–3)
**Myalgia**	0	1 (0.33, 1)	2 (0.66, 1)	3 (1, 2)	6	1.5	(1–4)
**Feverishness**	0	0	0	3 (1, 2)	3	2	(1–5)
**Headache**	2 (0.66, 2)	0	0	3 (1, 1)	5	2	(1–4)
**Fatigue**	1 (0.33, 1)	1 (0.33, 1)	1 (0.33, 1)	3 (1, 2)	6	2.5	(1–13)
**Nausea**	0	0	0	0	0	–	–
**Malaise**	0	0	0	3 (1, 1)	3	1	(1–14)
**Cough**	0	0	1 (0.33, 1)	3 (1, 2)	4	2.5	(1–162)
**Sore throat**	0	1 (0.33, 1)	0	2 (0.66, 1)	3	2	(2–5)
**Tickly throat**	1 (0.33, 1)	1 (0.33, 1)	2 (0.66, 1)	1 (0.33, 1)	5	1	(1–6)
**Wheeze**	0	0	0	2 (0.66,1)	2	1.5	(1–2)
**SOB**	0	0	0	1 (0.33, 1)	1	4	–
**Cough phlegm**	0	0	0	2 (0.66,1)	2	1.5	(1–2)
**Cough blood**	0	0	0	0	0	–	–
**Chest tightness**	0	0	0	2 (0.66, 1)	2	2.5	(2–3)
**Chest pain**	0	0	0	0	0	–	–

Grading range 1–3, AE presented here started within the first 72 h of e-Diary self-reporting. Group 1 = 1 × 10^4^ CFU; group 2 = 1 × 10^5^ CFU; group 3 = 1 × 10^6^ CFU; group 4 = 1 × 10^7^ CFU BCG SSI. SOB, shortness of breath.

**Figure 2:**
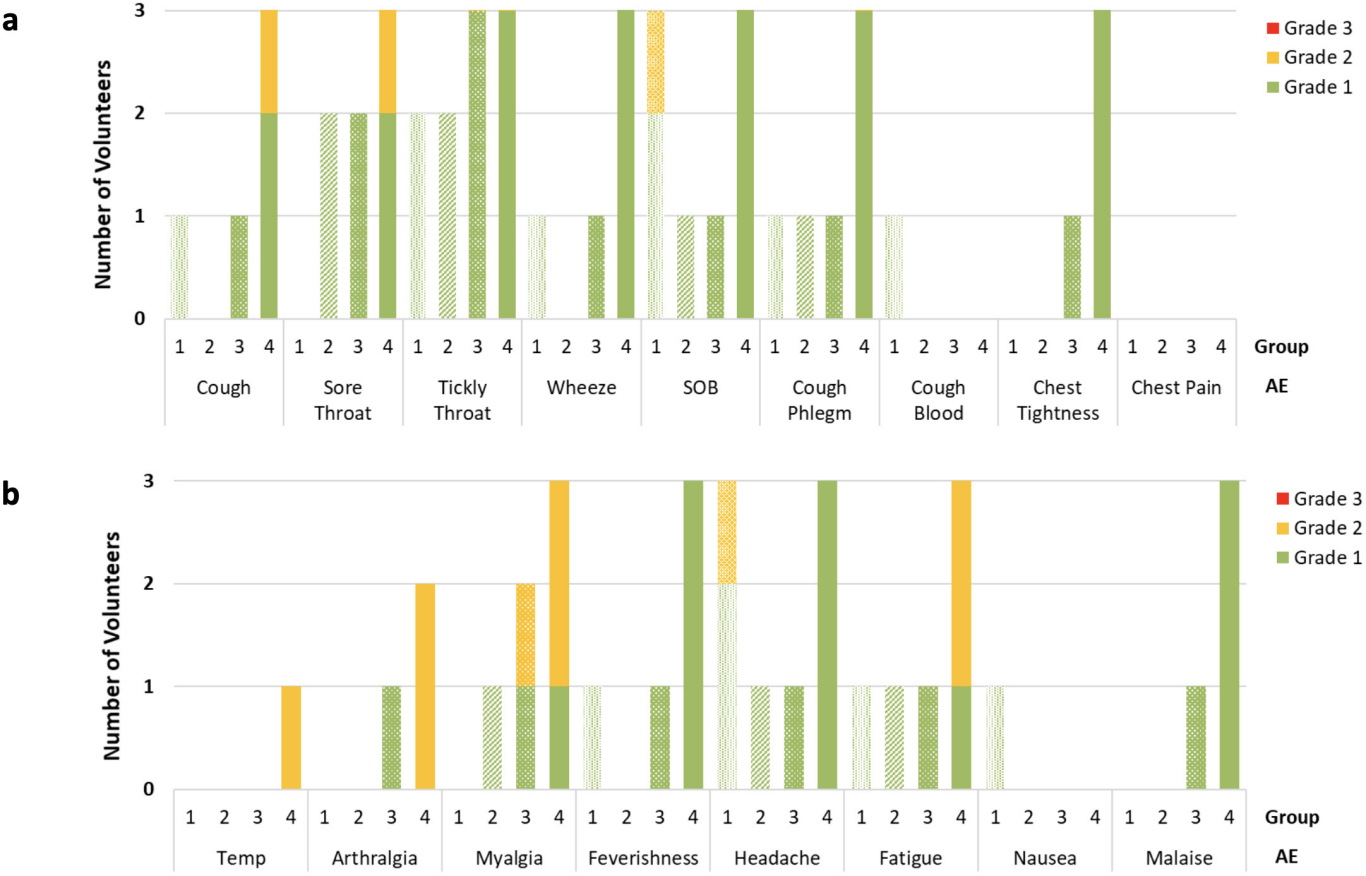
Number and maximum grade reported of respiratory **(A)** and systemic **(B)** adverse (AE) occurrence on daily volunteer diary in the 2 weeks following BCG aerosol challenge. AE’s reported split by group, dose of aerosol BCG delivered; Group 1 = 1 × 10^4^ CFU; Group 2 = 1 × 10^5^ CFU; Group 3 = 1 × 10^6^ CFU; Group 4 = 1 × 10^7^ CFU. Data obtained once daily in all cases, and volunteers in each group n=3. SOB, Shortness Breath.

The maximum grade and frequency of each solicited systemic and respiratory AEs reported in the first 14 days after aerosol BCG challenge are shown in **Figure 2**.

The most frequently reported systemic AEs after challenge were fatigue and headache, whereas the most frequently reported respiratory AEs were cough and tickly throat. There were no solicited AEs which were reported in the 14 days after aerosol BCG challenge, and not reported by any volunteers after bronchoscopy. One individual in group 4 reported a cough from D1 which continued throughout the diary and remained present at the end of the study, albeit at significantly reduced intensity. This volunteer also reported 2 days of grade 2 fever and an increased frequency of symptoms in the first 72 h following BCG challenge compared with other volunteers. **Figure 1 Supplementary Appendix** details the duration and severity of all solicited symptoms for this individual. Investigations including chest radiographs, blood cultures (conventional and mycobacterial), respiratory culture, and PCR were completed to rule out alternative causes. An initial chest radiograph taken on D2 revealed nodularity in the right middle lobe, spirometry on the same day was normal, and TL_CO_ on D7 was not significantly different from baseline. Macroscopic appearances of the bronchial mucosa inspected at the D14 bronchoscopy were unremarkable. Repeat chest radiograph 7 weeks later showed complete resolution of radiological changes. No alternative causative organisms were identified, and mycobacterial blood cultures were negative as was induced sputum at the day D168 visit.

More AEs were reported in volunteers in group 4 compared with groups 1 to 3, both for days of reported systemic AEs (68/335 vs. 36/1007 p<0.0001) and for respiratory AEs (18/1134 vs. 63/378 p<0.0001).

### Solicited adverse events reported in the 14 days following bronchoscopy

All bronchoscopies were completed without complication, and all were reported macroscopically as showing normal mucosa by the conducting consultant physician. Solicited AEs occurred in all study groups after bronchoscopy. Symptoms were consistent with those expected following all bronchoscopies. The most frequently reported AEs were cough, sore throat, tickly throat, and fatigue.

The median duration of AEs starting within the first 72 h of bronchoscopy was between 1 and 3 days in all cases (**Supplementary Table 2**). The frequency of AEs reported was similar between groups, and the increased frequency of AEs reported in group 4 compared with groups 1–3 after BCG challenge was not evident after bronchoscopy (53/714 vs. 171/2141 p=0.63).

The maximum grade for AEs after bronchoscopy was 2, apart from one individual who reported 2 days of grade 3 fever on D15 and D16 of the study. Upon PCR testing, this individual was found to have a nasopharyngeal swab which was positive for influenza A. Mycobacterial blood cultures were negative for growth after 6 weeks of incubation, and induced sputum at D168 was negative for BCG. One other volunteer had a grade 1 fever following bronchoscopy; as previously discussed, this individual also had negative mycobacterial blood cultures.

Overall, a greater number of AEs were reported in the 14 days after bronchoscopy than after aerosol BCG challenge (224/2,855 vs. 185/2,854 p=0.046). Separate analysis of each group showed the same association for groups 1 to 3; however, the trend was reversed in group 4 where significantly fewer AEs were reported after bronchoscopy than after aerosol BCG challenge (53/714 vs. 131/713 p<0.0001).

### Lung function tests

**Figure 3:**
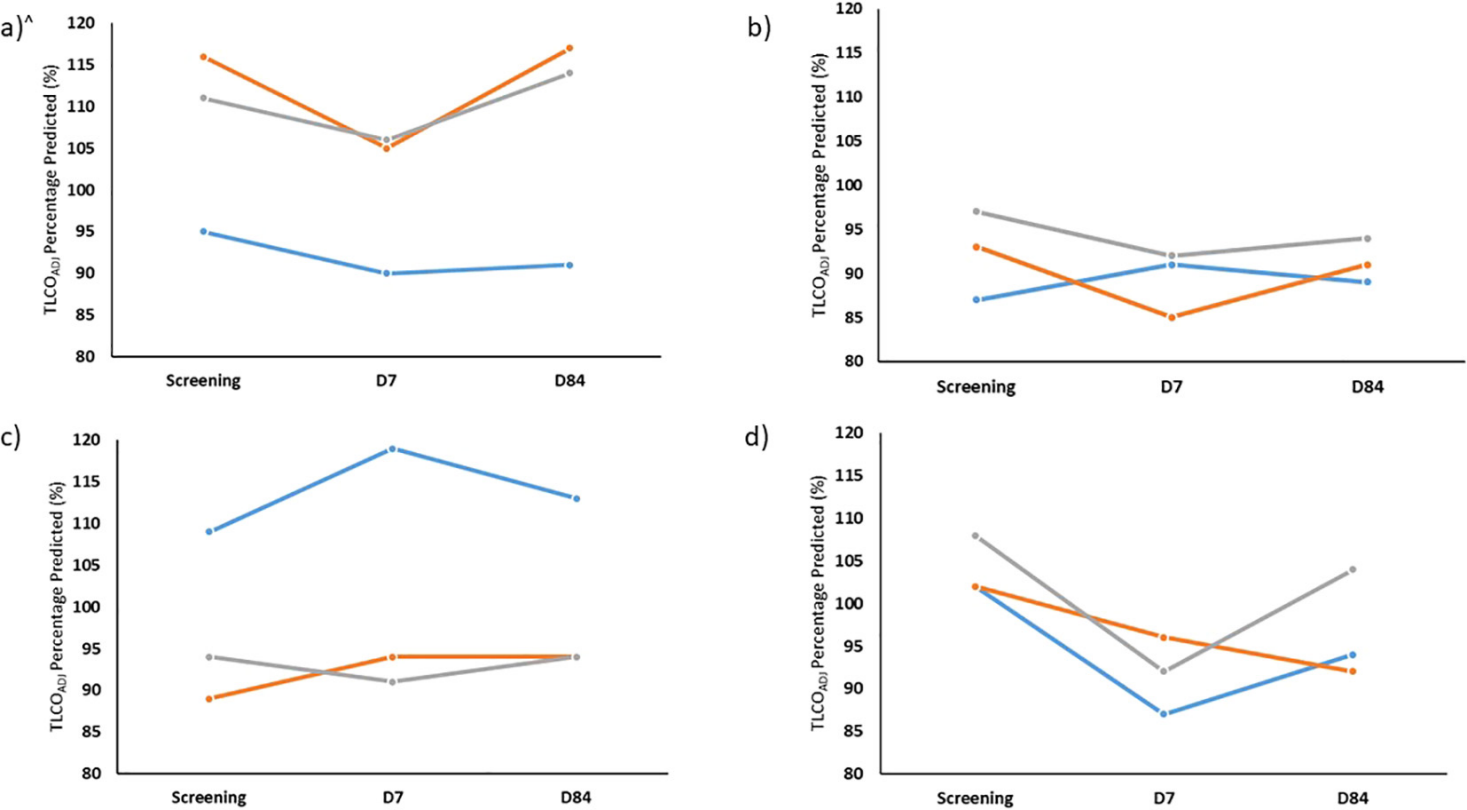
Percentage of predicted TLCO (TLCO_ADJ_) at screening and also 7 days (D7) and 84 days (D84) after aerosol BCG for Group 1 **(A)**, Group 2 **(B)**, Group 3 **(C)** and Group 4 **(D)** for each volunteer enrolled. TLCO, transfer capacity of the lung, for the carbon monoxide. All percentage predicted accounting for age, sex, ethnicity and height and adjusted for most recent laboratory haemoglobin. N.B. volunteer in blue TLCO repeated at D168 rather than D84.

None of the volunteers were found to have a clinically significant drop in Transfer Factor for carbon monoxide (TL_CO_) or transfer coefficient of the lung for carbon monoxide (K_CO_) at D7 after administration of BCG. Although the sample size was too small for statistical tests to be completed, there was a trend for greater drops in TL_CO_ and K_CO_ in group 4 compared with groups 1 to 3 (−11.7% vs. −2.3%, −8.3% vs. −2.5% mean change for TL_CO_ and K_CO_, respectively. Oxygen saturations were within the normal range at all study visits. Repeat TL_CO_ and K_CO_ at D84 remained within the expected variability of the test for all volunteers. **Figure 3** shows the TL_CO_ adjusted for haemoglobin (TL_COADJ_) for each group.

**Figure 4:**
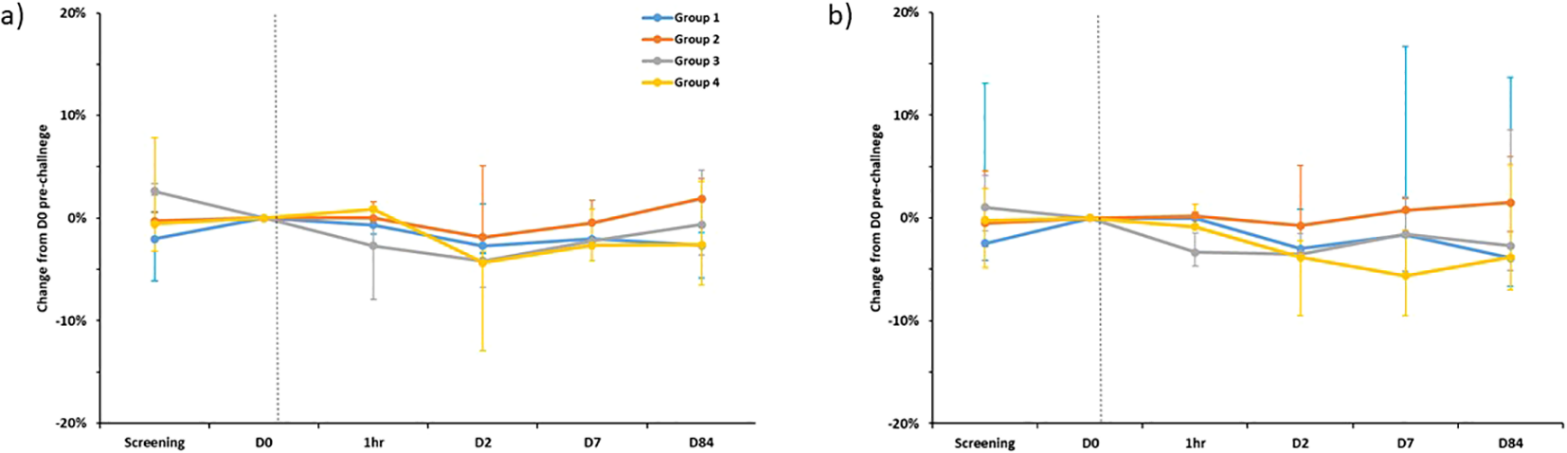
Percentage change in FEV1 **(A)** and FVC **(B)** compared to pre-challenge (D0) testing, separated by group. Plotted points are the median percentage change for each group. Bars show range. FEV1 - Forced Expiratory Volume in 1^st^ second, FVC, Forced Vital Capacity. Dotted line is time of BCG administration. D0 spirometry was completed on the day of enrolment immediately prior to challenge. 1hr- repeated spirometry taken one hour after challenge with BCG was completed. N.B Spirometry for one individual in group 1 completed at D168 rather than D84 visit.

Forced vital capacity (FVC) and forced expiratory volume in one second (FEV1) were similar across all groups and are shown in **Figure 4**. No significant drops in FVC or FEV1 were seen at any timepoint.

### Adverse safety blood tests

Haematology and biochemistry adverse events were rare. Four cases of hypokalaemia were identified after enrolment (grade 1: n=2, grade 2: n=1, grade 3: n=1); these were found to be normal on repeat testing and thought likely to be related delays in processing and raised ambient temperature, as described in other studies based at the same site (31). One individual was found to have an elevated eosinophil count (grade 1) on D14, which normalised by the time of repeat testing on D28. Another volunteer was found to have a transient rise in urea on D7 which normalised by D14; this individual reported use of protein supplements which were thought to have contributed to this result. Two volunteers were found to have grade 1 anaemia during follow-up. These were found after day 14 of the study and were static on repeat testing. They did not necessitate changes to the protocolised blood draws.

One volunteer in the high-dose group 4, previously discussed as having a larger number of AEs after aerosol BCG-challenge, was found to have a transient raise in bilirubin (grade 2) and alanine transaminase (grade 1) on D2 of the study; this was accompanied by a raised white cell count (grade 1) and grade 1 hyponatraemia. These AEs all normalised on repeat testing by D14. This individual also had a raised C-reactive protein measured at D2 of 209.2 mg/L (normal laboratory range 0 mg/L–5 mg/L), which gradually reduced on repeat testing to 19.2 mg/L by D7 and 6.2 mg/L by D28.

### BCG infection dose

**Figure 5:**
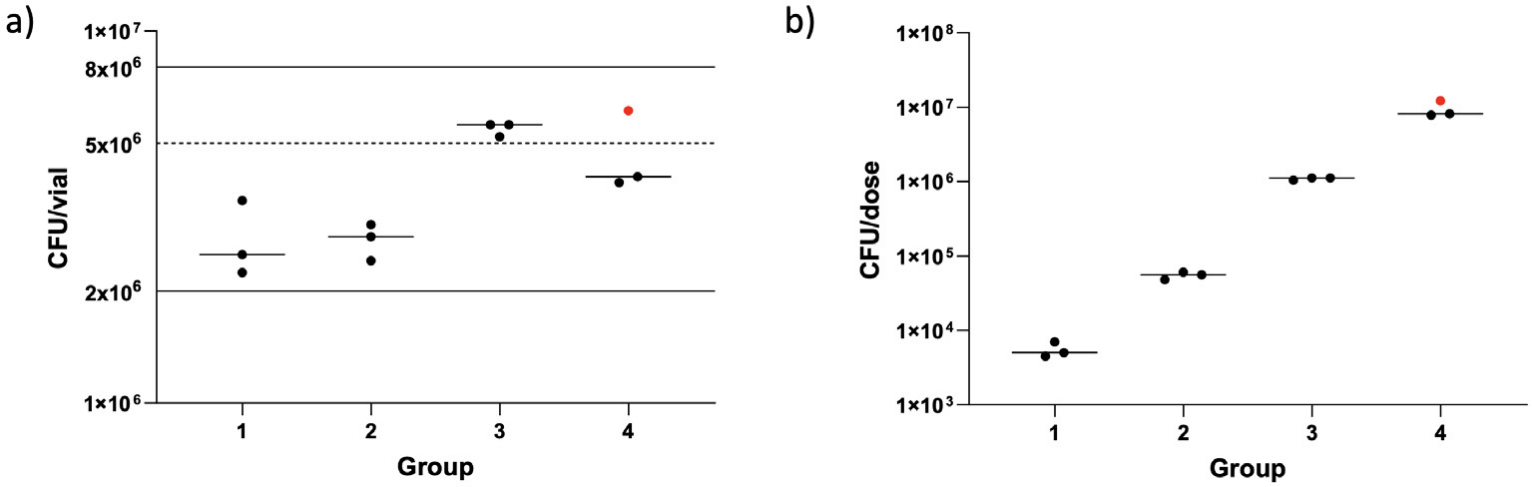
Enumeration of BCG from clinical vaccine vials **(A)** and dose loaded into the nebulizer **(B)**. Dots represent individual vaccine vials/dose per volunteer. Red dot shows a different batch of BCG used. Lines show median numbers per group, solid lines show the manufacture’s stated range for amount of BCG contained in a vaccine vial and the dotted line shows value used for dose calculations.

BCG was quantified, by BACTEC™ MGIT, from the diluted vaccine vials used to prepare the dose for nebulisation. The same batch of BCG was used for 11/12 volunteers; a new batch was used for the final volunteer enrolled due to the first batch having passed its expiry date. The number of BCG CFU/vial and number of BCG CFU/dose were calculated for each volunteer (**Figure 5**). The number of BCG CFU/vial ranged from 2.24 × 10^6^– 6.11 × 10^6^ (median 3.71 × 10^6^), with some variation between vials used for different groups; however, all were within the range stated by the manufacturer (2–8 × 10^6^ CFU/vial). The median dose given to volunteers in groups 1–4 was 5.01 × 10^3^, 5.60 × 10^4^, 1.12 × 10^6^, and 8.12 × 10^6^ CFU/vial, respectively. This was within 0.5 logs of the intended dose for each group.

### BCG was not detectable in BAL and sputum samples

BAL samples were pelleted, decontaminated, and added to BACTEC™ MGIT tubes for detection of live BCG 14 days post-aerosol BCG. No growth was detected in 11/12 BAL samples; one sample in group 2 became positive after 813 h. Out of the 12 volunteers in the study, 11 gave an induced sputum sample at D168 post-aerosol BCG; these samples were processed in the same way as the BAL samples for detection of growth. No growth was detected in 8/11 samples, with the other 3 becoming positive at 56 h, 545 h, and 636 h. DNA extraction and use of HAIN MTBC genotyping on all positive samples found that this growth was not BCG but identified as “High GC Gram Positive Bacterium”.

### BCG identified from PVA strips from face mask sampling

Two PVA strips from each mask, worn pre- and immediately post-aerosol BCG and 2 and 7 days post-aerosol BCG, in the two high-dose groups were dissolved, decontaminated, and added to BACTEC™ MGIT tubes for detection of live BCG. No growth of BCG was detected in any of the samples processed.

**Figure 6:**
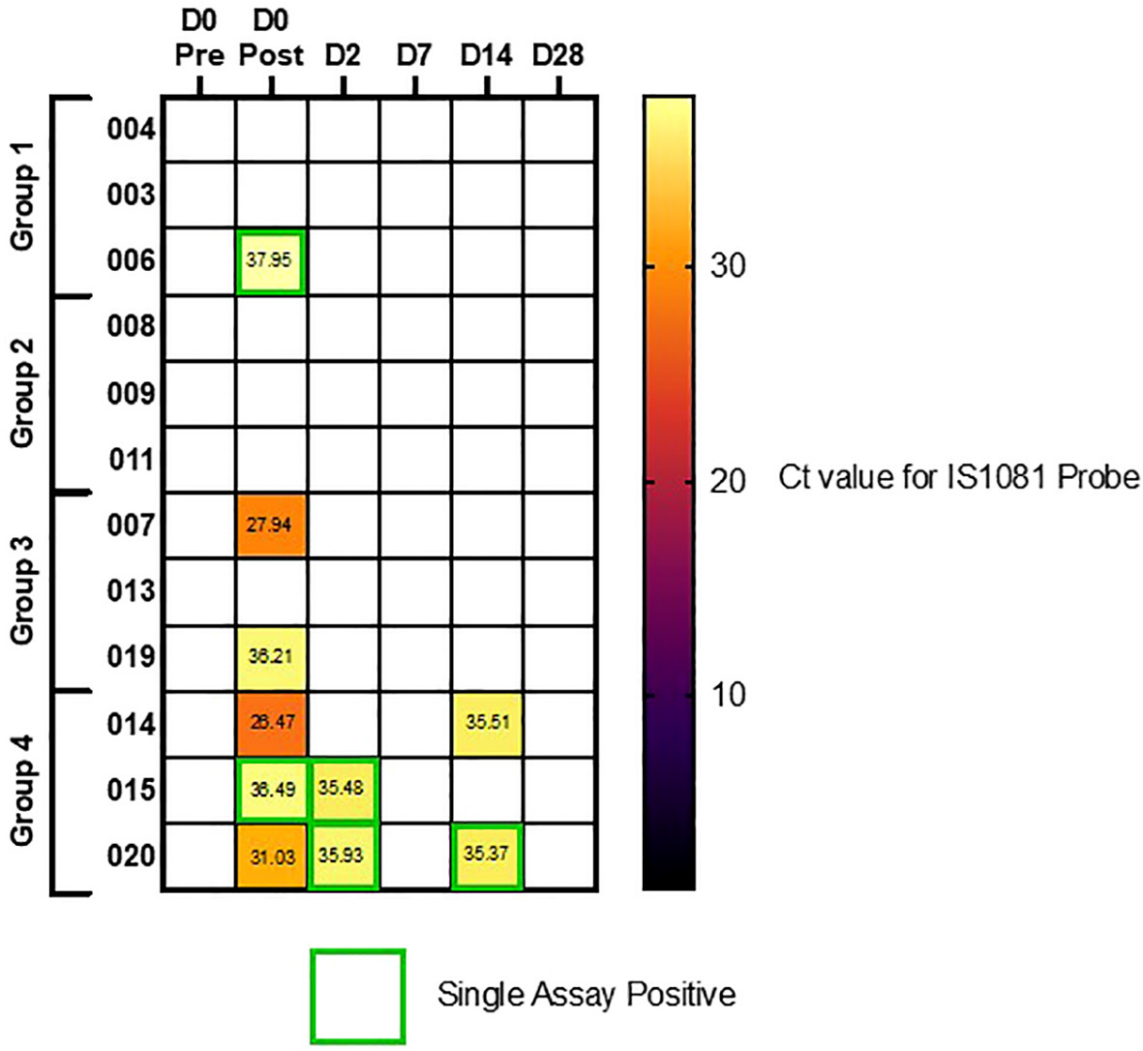
Heatmap of qPCR results using IS1081 probe, from PVA masks. Volunteers separated by dosing group (Group 1 = 1 x 10^4^ CFU, Group 2 = 1 x 10^5^ CFU, Group 3 = 1 x 10^6^ CFU and group 4 = 1 x 10^7^ CFU). Single assay positive probes highlighted in green boxes, where secondary IS6110 probe was not also positive.

qPCR performed on the remaining PVA strips showed detectable evidence of the *Mycobacterium bovis* BCG in 5/60 samples taken after infection in two target genes, whereas a further five post-infection samples were positive using a single BCG target gene. None of the pre-infection samples were positive for BCG. Seven out of 10 of the samples with detectable BCG were from volunteers in group 4, two out of 12 were detected in group 3, and one other sample was positive at D0 in group 1. **Figure 6** shows a heat map of the Ct values calculated for all mask samples processed, by volunteer and time point.

### *Ex vivo* IFN-γ ELISpot responses

**Figure 7:**
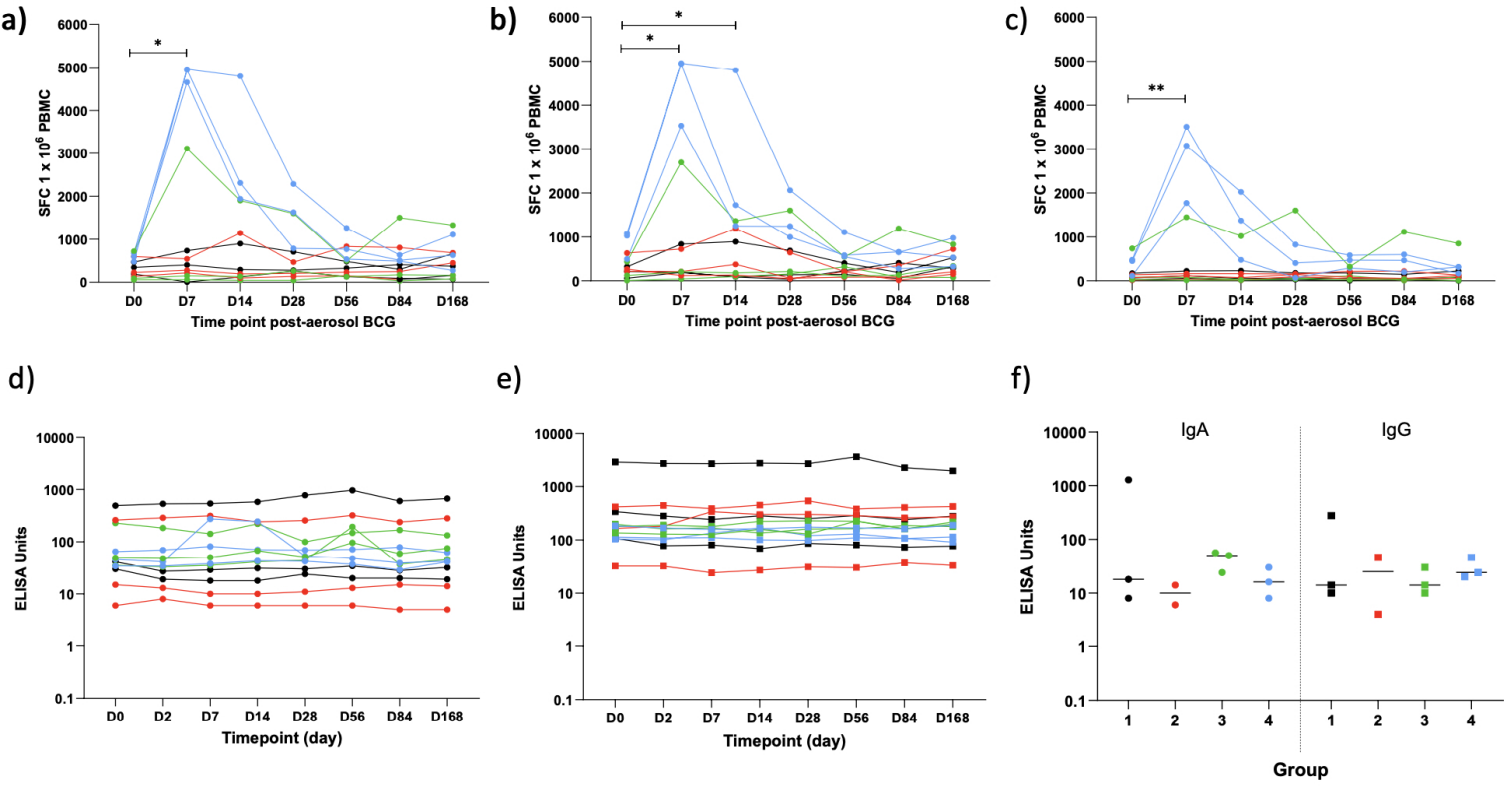
IFN-γ ELISpot responses to PPD **(A)**, BCG **(B)** and a pool of *M.tb* peptides - MTB300 **(C)** and ELISA PPD-specific IgA **(D)** and IgG **(E)** in serum at baseline and multiple time points post-aerosol BCG and in concentrated BAL fluid 14 days post-aerosol BCG **(F)**. Dots represent individual values, lines represent a volunteers response over time. Colours represent the different dose groups - black = 1 x 10^4^ CFU, red = 1 x 10^5^ CFU, green = 1 x 10^6^ CFU and blue = 1 x 10^7^ CFU. Stars denote significance *p = <0.05, **p = <0.01.

Fresh PBMC IFN-γ ELISpot responses to PPD, BCG, and a pool of MTB300 peptides were measured at D0 and at days 7, 14, 28, 56, 84, and 168 post-aerosol BCG. Due to only having three volunteers per dose group, it was not possible to perform statistical comparisons between them. However, a clear dose effect could be seen, with volunteers in the highest dose group having the biggest increase in responses between baseline and the peak of the response at D7 (**Table 3**). All groups were combined to compare responses at each time point to baseline (**Figure 7**). PPD, BCG, and MTB300 responses at D7 were all significantly higher than baseline (p = 0.020, 0.021, and 0.0034 respectively, Wilcoxon matched-pairs); only BCG responses remained significantly higher than baseline at D14 (p = 0.016), and no other time points were significantly different from baseline.

**Table 3:** Median IFN-γ ELISpot responses per million PBMC by dose group.

Time point (days)	PPD				BCG				MTB300			
	Group 1	Group 2	Group 3	Group 4	Group 1	Group 2	Group 3	Group 4	Group 1	Group 2	Group 3	Group 4
**0**	343	228	91	477	213	266	123	1,028	68	72	83	450
**7**	401	280	149	4,943	192	209	206	4,943	117	114	79	3,077
**14**	291	194	132	2,313	90	373	181	1,723	53	74	34	1,373
**28**	279	204	260	1,627	138	59	214	1,242	67	144	49	406
**56**	326	234	153	766	229	204	318	584	54	80	114	471
**84**	300	247	172	508	188	86	136	651	44	57	40	467
**168**	372	451	160	626	322	203	338	532	76	117	91	314

Group 1 = 1 × 10^4^ CFU; group 2 = 1 × 10^5^ CFU; group 3 = 1 × 10^6^ CFU; group 4 = 1 × 10^7^ CFU BCG SSI.

### PPD-specific IgA and IgG antibody responses

PPD-specific IgA and IgG were measured in the serum of all volunteers at D0 and 2, 7, 14, 28, 56, 84, and 168 days post-aerosol BCG and in concentrated BAL fluid at D14 for 11/12 volunteers (one volunteer’s BAL fluid was lost due to a processing error; **Figure 7**). There was no noticeable dose effect in the serum, so all 12 volunteers were combined for analysis; there was no significant increase in either IgA or IgG over time compared with baseline (Friedman test). Levels of PPD-specific IgA and IgG in the BAL fluid were generally lower than those in the serum, with no noticeable dose effect, but with only three samples in each group, it was not possible to do a statistical comparison.

### Direct PBMC mycobacterial growth inhibition assay

**Figure 8:**
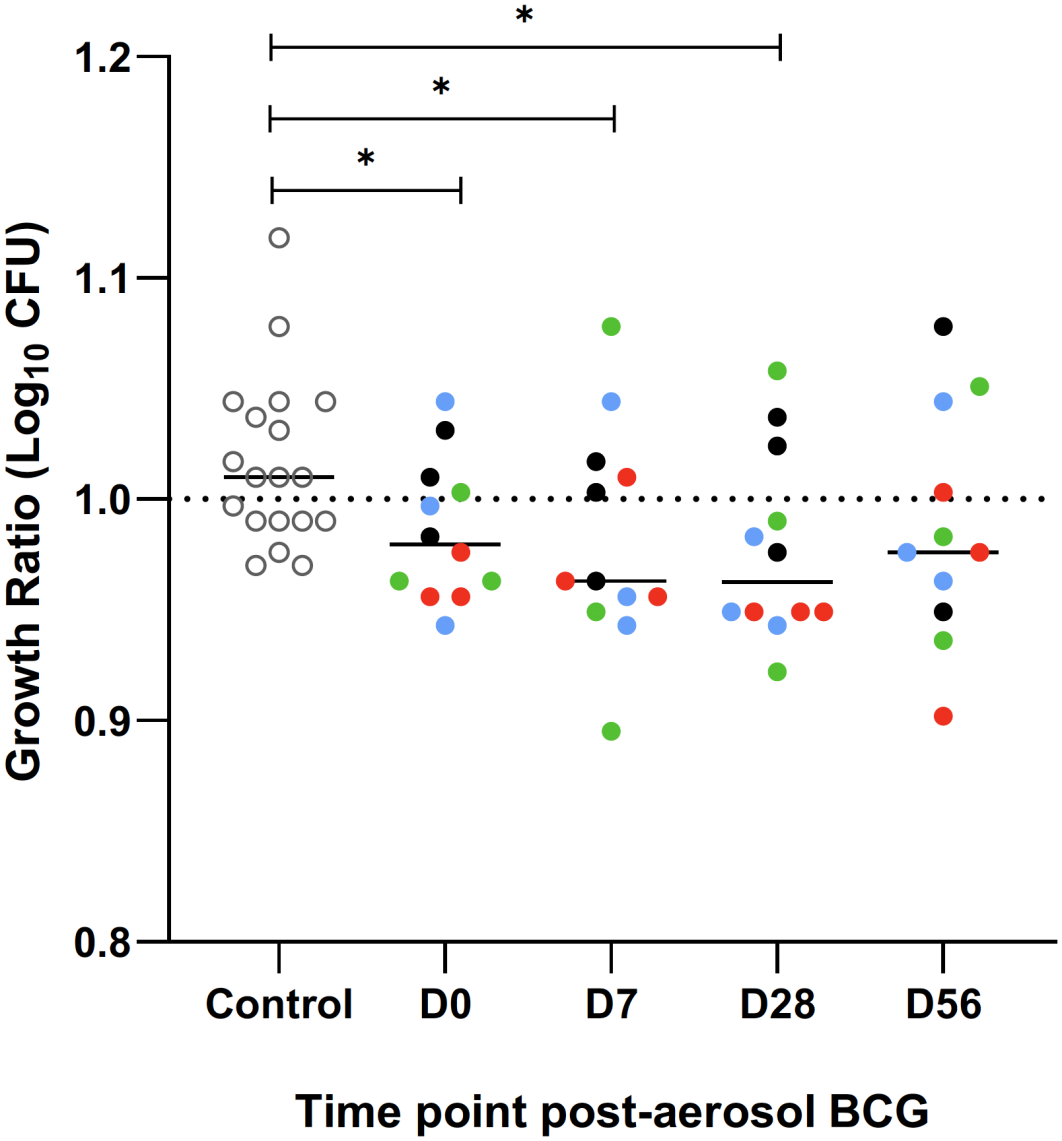
Ratio of BCG growth after a 96 hour incubation with PBMC, collected and frozen on day of aerosol BCG and 7, 28 and 56 days post-aerosol BCG, in the mycobacterial growth inhibition assay (MGIA). A group of BCG naïve volunteers (open circles) were run as a control for the assay. Colours represent the different dose groups - black = 1 x 10^4^ CFU, red = 1 x 10^5^ CFU, green = 1 x 10^6^ CFU and blue = 1 x 10^7^ CFU. Stars denote significance *p = <0.05.

The direct MGIA was performed in one batch on PBMC frozen at baseline and at 7, 28, and 56 days post-aerosol BCG from all volunteers. PBMC from 19 BCG naïve volunteers were run alongside to act as a control for the assay, and there was a significant reduction in the log10 CFU growth ratio in the historically BCG-vaccinated group at baseline compared with the naïve group (p = 0.023, Mann–Whitney; **Figure 8**). There was a trend towards better control of BCG growth after aerosol BCG, with lower median growth ratios at D7 and D28 compared with baseline, but this did not reach statistical significance.

## Discussion

This is the first aerosol-BCG CHIM to be completed on historically BCG-vaccinated volunteers. We have shown that aerosol challenge, which closely mimics the route of infection of *M.tb*, can be safely performed up to a loaded dose of 1 × 10^7^ CFU BCG Danish. Despite some variability in the frequency and severity of AEs between volunteers, this route of challenge and the following bronchoscopy was well-tolerated by volunteers.

All volunteers experienced some systemic and respiratory symptoms during the 28-day eDiary; however, no volunteers experienced severe (grade 3) symptoms as a direct result of BCG challenge and there were no serious adverse events in this study. The number of AEs reported within the first 72 h of challenge was greater in the highest-dose group (group 4), where both the frequency and maximum severity of systemic and respiratory symptoms increased. Despite the dose effect, even at the highest dose, the duration of AEs was generally short. Symptoms were predictable, with few unsolicited AEs recorded, and the most commonly reported AEs of cough, sore, or tickly throat and fatigue were generally mild, with only 10.4% of reported AEs more severe than grade 1. Compared with a previous aerosol BCG CHIM, which we have completed on BCG-naïve volunteers (18), the most commonly reported AEs were similar in type. Although the sample size of groups in this study are small, there does appear to be a trend for more symptoms in the 72 h following challenge in historically BCG vaccinated individuals than was seen previously in BCG-naïve volunteers (18). It is credible that a stronger and more rapid adaptive immunological response secondary to immunological memory meant that historically vaccinated individuals had a larger symptom burden. However, it is also important to note that this study was not blinded, whereas some aspects of our previous BCG-naïve study were, and this may have affected the rate of reporting.

AEs reported following bronchoscopy were also predictable and of a similar frequency to those reported in another healthy volunteer cohort (32). It was reassuring that we did not see a dose effect on the frequency of AEs post bronchoscopy, suggesting that BAL sampling at day 14 does not pose a significant risk of potentiating BCG infection after challenge; this is also in keeping with results from previous work in BCG naive volunteers, where there was no difference in the proportion of AEs reported by aerosol BCG and control groups (18, 33).

Comparison of symptom burden to another BCG respiratory challenge study published by Davids et al. is pertinent, as this study was also conducted on individuals who were historically BCG vaccinated, although some also had evidence of *M.tb* infection or previous disease (17). There are significant differences in methodology which make comparisons challenging, foremost that BCG was administered via bronchoscopy and only to one lobe of the lung and at a different dose (1 × 10^4^ CFU). The authors of this study state that 70% of individuals reported an AE deemed to be related to their bronchoscopy, whereas they concluded that there were no AEs definitively related to BCG (17). Making a clear distinction on causality, when the two events occur at the same timepoint, is challenging. We have shown that AEs related to BCG challenge and bronchoscopy are similar and we found no symptoms unique to each phase of our study. While aerosol BCG has historically been trialled as a treatment for lung carcinoma, the underlying pathology and concurrent use of other treatment make comparison difficult (34, 35).

One individual in group 4 of this study had markedly more symptoms within the first 72 h of challenge than were reported by other volunteers. This included several grade 2 symptoms: fever lasting 2 days, cough, arthralgia, myalgia, and fatigue as well as night sweats lasting 11 days. Symptoms were accompanied by a significantly raised C-reactive protein on day 2. It is difficult to be certain why this individual had such marked symptoms. Baseline characteristics, including time since initial BCG vaccination, was not significantly different to other volunteers. We considered whether this individual could have developed a BCGosis; however, serial mycobacterial blood cultures were negative, and the acuity of symptoms and speed of recovery, without the need for additional treatment, did not support this. Although we did not find microbiological evidence for a concurrent viral or bacterial infection, we cannot entirely rule out this possibility as routine diagnostics for respiratory infections do not yield a causative organism in the majority of cases (36). Given the similarity in time course of symptom onset, it is likely that a major driver was a systemic response triggered by BCG re-exposure. This is supported by the large BCG-specific ELISpot response seen for this individual, and the transitory chest radiograph changes. Although this volunteer had a mild ongoing cough even at final visit on day 168, this was significantly improved, with the volunteer reporting only an occasional non-productive cough occurring very intermittently which was not affecting any of their normal activities. Causality in this regard is also difficult as this volunteer had been given a diagnosis of gastric reflux prior to starting the study, which is a common cause of chronic cough (37). The volunteer had elected not to initiate any preventative therapy offered for their diagnosis of reflux, and therefore we cannot rule out this co-existing diagnosis as contributory. Given the small number of volunteers recruited to the highest BCG dose, recruitment of more volunteers would be of benefit to evaluate the spectrum of symptoms experienced after aerosol BCG challenge in historically vaccinated individuals at a dose of 1 × 10^7^ CFU/dose. There is significant heterogeneity in the side effect profile of those who receive intravesicular BCG for the treatment of bladder neoplasms (38), and it is likely that the same is true of BCG administered through the respiratory system.

We found no evidence of Koch phenomenon (39) after re-exposure to BCG, and despite frequent monitoring of lung function tests, there were no clinically significant drops in any of the functional tests. We did note a trend for larger drops from baseline of TL_CO_ and K_CO_ at day 7 in group 4. Measurement of TL_CO_ is known to be highly variable; even after appropriate adjustment for demographics and haemoglobin, it has been shown to be affected by recent exercise and food intake as well as diurnal variations and timing of the menstrual cycle (40–42). Pulmonary function tests are also well established to be effort dependent (43), and this may explain some of the trend seen when compared with screening results. Given the inflammatory responses observed in volunteers, modest changes to lung TL_CO_ could be expected. TL_CO_ is not routinely used in clinical practice to assess response to infection; however, a study of adult patients admitted with acute varicella zoster infection showed that the transfer factor was significantly reduced in the majority of individuals following infection, even in the absence of a diagnosis of pneumonia (44). Volunteers with small reductions in TL_CO_ in our study did not report associated clinical symptoms of breathlessness and had normal observations recorded and unremarkable physical examination at study visits. Reassuringly, we noted that the size of differences from baseline diminished upon retesting at day 84.

No BCG was detectable by BACTEC™ MGIT in BAL samples 14 days post-BCG challenge in any of the volunteers. The reasons for this are not immediately clear. We have previously shown that viable BCG can be consistently cultured from BCG-naïve healthy UK adults following challenge with a dose as low as 1 × 10^4^ CFU BCG Bulgaria (18). It is conceivable that the immunological memory afforded by historic intradermal BCG vaccination for these individuals aided clearance prior to D14 or that there is a BCG strain affect. BCG conferred 80% protection in the British MRC study, and this potential demonstration of a known BCG vaccine effect in our aerosol BCG infection model provides data to support the biological validity of this model (45). In a different respiratory BCG CHIM, Davids et al. instilled a dose of 1 × 10^4^ CFU BCG SSI (Danish) into a single lung segment of historically BCG-vaccinated volunteers, some of whom also had evidence of *M.tb* infection or previous disease (17). BCG was only recovered in 6 of 54 volunteers from a BAL completed 3 days after initial BCG challenge. Despite differences both in the modes of delivery, dose of BCG, and timing of bronchoscopy, it is credible in both studies that enhanced clearance was afforded from immunological memory of primary vaccination. As the sample size in each dosing group of our study was small, we cannot discount the possibility of successfully recovering BCG in a significant proportion of individuals. It is possible that the sensitivity to detect BCG in our study was hampered by the processing of BAL fluid samples. To reduce the risk of contamination by other typical bacteria, lavage samples underwent decontamination with MycoPrep™. NALC-NaOH-based approaches to decontamination such as MycoPrep™ are known to significantly reduce the viability of *M.tb* (46)., and it is credible that the same would be the case for BCG. Alternative processing techniques may improve the sensitivity of culture but would run a significant risk of contamination with other bacteria from the lung or oropharynx.

qPCR evidence of exhaled BCG was detected from face mask sampling in all three volunteers from group 4 immediately after challenge and from 2/3 volunteers in group 3. Previous studies have used this technology for identification of cases of incipient *M.tb* infections in those with subclinical disease, where it has been shown to have the potential to provide earlier diagnosis than sampling sputum (47), and an improved predictor of transmissibility (48). The larger proportion of positive samples in group 4 suggests a dose effect and provides support for the use of higher inoculums of BCG in this aerosol challenge model. It is notable that we were not able to isolate live BCG on any exhaled PVA strip samples after incubation in MGIT. As previously discussed, the sensitivity of culture is also likely to be reduced by the decontamination of samples with MycoPrep™, as has previously been reported during development of these adapted face masks (49). However, these data do provide a reassuring safety signal as it suggests that volunteers in this study do not pose a significant risk to close contacts even immediately after administration of aerosol BCG.

Two volunteers in group 4 also had positive PCRs on D14, despite having no culturable BCG in their BAL sample. This may be related to issues with sensitivity, but it is also credible that this constitutes genetic material from non-viable bacilli. Equally masks were worn for a relatively short time period (30 min) during which volunteers breathed normally. Although few studies have focussed directly on means to optimise sampling of infectious agents from adapted facemasks, the optimal duration of sampling is not established (50). Previous experience of face mask sampling with *M.tb* has highlighted that sensitivity of this tool is likely to be improved with prolonged durations of mask wearing (49). Further work is needed to optimise the sensitivity of this emerging technology.

All volunteers in this study were shown to elicit significant adaptive immune responses to BCG infection. ELISpot responses were significantly increased compared with baseline. There was also evidence of a dose effect with volunteers in group 4 showing greater fold change compared with baseline when stimulated with PPD, BCG, and the MTB300 peptide pool. This time course is similar to what we have previously shown in BCG-naïve volunteers (18), and in an aerosol delivery study of the *M.tb* viral vector vaccine MVA85A (24), with peak responses occurring at day 7. Although we did not directly compare ELISpot responses between BCG-naïve and historically vaccinated individuals in this study, heightened ELISpot responses following revaccination with intradermal BCG have been reported in other studies (51). Further work to directly compare the magnitude of immunological responses between intradermal and aerosol routes after re-challenge would be valuable.

We did not see a significant increase in PPD-specific IgA and IgG responses compared with baseline in the serum of challenged volunteers, nor was there a significant difference in IgA and IgG levels in the BAL fluid between the different dose groups. The importance of antibodies in the clearance of mycobacteria remains contested and the results inconsistent (52). Data from a small study in Turkey showed that intradermal BCG causes increases in PPD-specific serum IgG after primary intradermal vaccination (53). There is evidence for increasing titres of antibodies to components of the cell wall in revaccination studies which may be more pertinent to our results. One group showed a detectable increase in IgG to lipoarabinomannan after primary intradermal BCG vaccination which increased again following intradermal re-vaccination 6 months later (54). It is possible that the time interval between primary and secondary exposure is important, as another group showed that while both primary and secondary vaccination resulted in transient increases from baseline antibody titres against arabinomannan, there was no difference in the maximal antibody responses between those who were BCG-naïve and those who had been vaccinated in childhood (55). Whether the aerosol route of delivery is important is not entirely clear. NHPs given primary aerosol BCG vaccination were shown to have a significant rise in serum PPD-specific IgG following primary aerosol vaccination (56). We have previously shown that there are significant increases from baseline in PPD-specific IgG and IgA responses in the serum after aerosol BCG (Bulgaria) vaccination, although IgG responses were short lived falling to baseline by day 14 (18). We are unaware of data reporting humoral responses to BCG revaccination through the aerosol route in animal or human models.

It is interesting that there was a trend towards better control in the MGIA assay after aerosol BCG, and that this was more marked in the highest-dosing group even though these data did not reach statistical significance. The MGIA is a functional sum-of-the-parts assay designed to establish whether the control of the combined immunological response to mycobacteria is enhanced (57). In this study, we completed the MGIA on frozen PBMC and did not include paired serum samples to minimise variability; this comes at the cost of negating the role of neutrophils and antibodies present in whole blood. The small sample size means that this study was not powered to draw clear conclusions but provides a basis to explore the role for aerosolised BCG as a vaccine candidate in its own right given non-human primate data, suggesting that mucosal BCG is more protective than intradermal BCG (58).

Guided by our previous work developing a BCG CHIM, we elected to utilise the entire BAL pellet for culture in order to improve the sensitivity of viable BCG detection (18). This has limited the ability to characterise the local pulmonary immunological responses to aerosol BCG in this study. Previous works conducted by our group and others have highlighted that local immune response kinetics can be very different to those seen in circulation after administration of BCG (17, 18). Given the successful dose escalation in this study, we plan to extensively interrogate the local immunological responses after challenge in the future cohort of historically BCG-vaccinated healthy adults. This is a key role for CHIM studies in accelerating vaccine research, providing an opportunity to identify novel corelates of protection and to identify novel vaccination targets and approaches (11).

In this study, we have shown that the aerosol BCG challenge is safe in historically BCG-vaccinated healthy adults up to a dose of 1 × 10^7^ CFU. Although we showed subjects mounted significant BCG-specific immunological responses, we were not able to culture BCG from BAL samples taken 14 days after challenge. Future work should focus on exploring ways to maximise sensitivity of culture techniques in large cohorts to investigate whether aerosol BCG CHIMs are useful in assessing prime-boost vaccine regimes involving historically BCG-vaccinated individuals. Aerosol BCG CHIMs remain a promising tool to accelerate TB vaccine development and as an avenue to further appraise the role of aerosolised vaccines for respiratory infections, which could provide advantages over more conventional routes.

## Data Availability

The original contributions presented in the study are included in the article/**Supplementary Material**. Further inquiries can be directed to the corresponding author.
