# Supplementary material for: A dose escalation study to evaluate the safety of an aerosol BCG infection in previously BCG-vaccinated healthy human UK adults
Source: Front Immunol. 2024 Nov 14;15:1427371. doi: 10.3389/fimmu.2024.1427371 (PMC11602284; doi:10.3389/fimmu.2024.1427371)
Supplement: Supplementary file 1 [file DataSheet1.pdf]

CLINICAL STUDY PROTOCOL

A clinical challenge study to evaluate controlled human infection with BCG administered by the aerosol inhaled route in historically BCG-vaccinated healthy adult volunteers

Short title: Aerosol BCG challenge study in historically BCG-vaccinated volunteers

**Study Reference:**	TB044
**Protocol Version:**	3.1
**Date:**	05 Aug 2022
**REC Reference:**	**20/SC/0059**
**IRAS Reference:**	276608

**Chief Investigator:**	Professor Helen McShane
**Sponsor:**	University of Oxford
**Funder:**	National Institutes of Health (NIH)/<br>National Institute of Allergy and Infectious Diseases (NIAID)
**Author:**	Dr Hazel Morrison, Dr Ingrid Cabrera Puig, Dr Susan Jackson, Dr Timothy Fredsgaard-Jones, Hannah Scott

Key Trial Contacts

**Chief Investigator**	Professor Helen McShane
**Address**	Centre for Clinical Vaccinology and Tropical Medicine<br>University of Oxford<br>Churchill Hospital, Old Road, Headington<br>Oxford, OX3 7LE<br>Tel: +44 (0)1865 617606<br>Email: helen.mcshane@ndm.ox.ac.uk
**Clinical Trials Unit**	Primary Care and Vaccines Collaborative CTU
**Other Investigators**	Dr Hazel Morrison<br>Lead Clinician, CCTVM<br><br>Dr Susan Jackson<br>Study clinician, CCVTM<br><br>Dr Timothy Fredsgaard-Jones<br>Study clinician, CCVTM
**Lead Nurse**	Raquel Lopez Ramon<br>Clinical Research Matron<br>CCTVM
**Collaborators**	Dr Henry Bettinson<br>Consultant Respiratory and Intensive Care Physician<br>Oxford Centre for Respiratory Medicine<br>Oxford University Hospitals NHS Foundation Trust<br>Oxford, OX3 7LE<br>Tel: +44 (0)1865 225234<br>Email: henry.bettinson@ouh.nhs.uk<br><br>Dr Timothy Hinks<br>Senior Research Fellow and Honorary Consultant<br>Respiratory Medicine Unit, NDM Experimental Medicine<br>University of Oxford Rm 7406<br>Level 7, John Radcliffe Hospital<br>Oxford, OX3 9DU<br>Tel: +44 (0)1865 220885<br>Email: timothy.hinks@ndm.ox.ac.uk<br><br>Professor Michael Barer<br>Professor of Clinical Microbiology and Honorary Consultant Microbiologist<br>Department of Respiratory Sciences<br>Maurice Shock Medical Sciences Building<br>University Road<br>Leicester LE1 9HN<br>Tel: +44 (0)116 252 2951/2933<br>Email: mrb19@leicester.ac.uk

**Project Managers**	Dr Rebecca Powell Doherty and Hannah Scott<br>CCTVM
**Senior Immunologist**	Dr Iman Satti<br>The Jenner Institute, Old Road Campus Research Building<br>Roosevelt Drive, Headington<br>Oxford, OX3 7DQ<br>Email: iman.satti@ndm.ox.ac.uk
**Sponsoring Institution**	**University of Oxford**<br>Research Governance, Ethics and Assurance<br>Joint Research Office,<br>Boundary Brook House<br>Churchill Drive, Headington<br>Oxford OX3 7GB<br>Email: ctrg@admin.ox.ac.uk
**Funder**	National Institutes of Health (NIH)/<br>National Institute of Allergy and Infectious Diseases (NIAID)
**Monitor**	Susan Tonks Centre for Clinical Vaccinology and Tropical Medicine,<br>University of Oxford,<br>Churchill Hospital,<br>Oxford OX7 3LE
**Safety Monitoring Committee**	Dr Francesca Little (Chair)<br>SMC Statistician<br>PD Hahn Building, 5.36<br>Upper Campus<br>University of Cape Town<br>Tel: +27 21 650 3225<br>Email: francesca.little@uct.ac.za<br><br>Dr Hassan Mahomed<br>Division of Health Systems and Public Health<br>University of Stellenbosch<br>Francie van Zijl Drive<br>Tygerberg, 7505<br>South Africa<br>Tel: +27 21 938 9111<br>Email: Hassan.Mahomed@westerncape.gov.za<br><br>Professor Stephen Gordon<br>Director Malawi-Liverpool-Wellcome Trust Programme<br>Queen Elizabeth Central Hospital<br>College of Medicine<br>Chichiri<br>Malawi<br>Tel: +265 1874628<br>Email: sgordon@mlw.mw stephen.gordon@lstmed.ac.uk

Confidentiality Statement
This document contains confidential information that must not be disclosed to anyone other than the Sponsor, the investigator team, HRA, host organisation, and members of the Research Ethics Committee and other regulatory bodies. This information cannot be used for any purpose other than the evaluation or conduct of the clinical investigation without the prior written consent of Professor Helen McShane.

Statement of Compliance
The study will be conducted in compliance with the protocol, the principles of Good Clinical Practice, Medicines for Human Use (Clinical Trial) Regulations 2004 (as amended) and all other applicable regulatory requirements.

Chief Investigator approval and agreement

"I have read this protocol and agree to abide by all provisions set forth therein. I agree to comply with the International Conference on Harmonisation Tripartite Guideline on Good Clinical Practice."

Professor Helen McShane	[signature]	15Aug2022
Chief Investigator	Investigator Signature	Date

"According to the Declaration of Helsinki, 2008, I have read and hereby approve this version of the protocol. I declare no conflict of interest"

Conflict of Interest Details: ____

____

Professor Helen McShane	[signature]	15Aug2022
Chief Investigator	Investigator Signature	Date

Table of Contents

1. AMENDMENT HISTORY 8
2. SYNOPSIS 10
3. ABBREVIATIONS 13
4. BACKGROUND AND RATIONALE 15
   4.1 Background 15
   4.2 Pre-Clinical Studies 16
   4.3 Previous clinical experience 16
   4.4 Rationale 17
   4.5 Face-mask sampling 21
   4.6 BCG Challenge Study and COVID-19 Pandemic 22
5. OBJECTIVES AND ENDPOINTS 23
6. STUDY DESIGN 24
   6.1 Study groups 24
   6.2 Study volunteers 24
   6.3 Definition of Start and End of Study 24
   6.4 Potential Risks for volunteers 25
   6.5 Known Potential Benefits 27
7. RECRUITMENT AND WITHDRAWAL OF STUDY VOLUNTEERS 27
   7.1 Identification of Study Volunteers 27
   7.2 Informed consent 28
   7.3 Inclusion and exclusion criteria 29
   7.4 Compliance with Dosing Regime 32
   7.5 Pregnancy 32
8. CLINICAL PROCEDURES 33
   8.1 Schedule of Attendance 33

8.2 Observations 33
8.3 Blood tests, urinalysis, bronchial samples 33
8.4 SARS-COV-2 Testing 34
8.5 Mask wearing 34
8.6 Diary Card 34
8.7 Study visits 35
9. STUDY AGENTS AND DEVICES 39
9.1 BCG Description 39
9.2 Dispensing and administration 39
9.3 Saline 39
9.4 Salbutamol 39
9.5 MicroAIR NE-U22/U100 39
9.6 Ultrasonic nebuliser NE-U780 40
9.7 Sedative & anaesthetic agents for bronchoscopy 40
10. ASSESSMENT OF SAFETY 41
10.1 Definitions 41
10.2 Expectedness 42
10.3 Causality 42
10.4 Reporting Procedures for All Adverse Events (see local SOP for AE reporting) 43
10.5 Assessment of severity 44
10.6 Reporting Procedures for Serious AEs 44
10.7 Procedures to be followed in the event of abnormal findings 45
10.8 Interim Safety Reviews 45
10.9 Safety Monitor Committee 45
10.10 Safety Group Holding Rules 45
11. STATISTICS 48
12. DATA MANAGEMENT 49
12.1 Data Handling 49

12.2 Record Keeping 49
12.3 Source Data and Case Report Forms (CRFs) 50
12.4 Data Protection 50
12.5 Data Quality 50
13. QUALITY CONTROL AND QUALITY ASSURANCE PROCEDURES 51
13.1 Investigator procedures 51
13.2 Monitoring 51
13.3 Protocol deviation 51
13.4 Audit & inspection 51
14. SERIOUS BREACHES 51
15. ETHICS AND REGULATORY CONSIDERATIONS 52
15.1 Declaration of Helsinki 52
15.2 Guidelines for Good Clinical Practice 52
15.3 Ethical Approvals 52
15.4 Volunteer Confidentiality 52
16. FINANCING AND INSURANCE 53
16.1 Financing 53
16.2 Insurance 53
16.3 Compensation 53
17. PUBLICATION POLICY 53
18. REFERENCES 54
19. Appendices 57
Appendix A: Covid 19 study specific self-isolation guidance (Risk assessment) 57

1. AMENDMENT HISTORY

Amendment No.	Protocol Version No.	Date issued	Author(s) of changes	Details of Changes made
NSA01	3.1	03Aug 2022	Timothy Fredsgaard-Jones	Dr Timothy Fredsgaard-Jones added as author and study investigator. Miss Hannah Scott added as author<br><br>The exclusion criteria 'Any autoimmune conditions or immunodeficiency (including HIV)' has been joined with the prior bullet point, listing exclusion criteria of clinically significant conditions. This was erroneously formatted into a separate bullet point, and therefore missing the standard caveat of clinically significant.
SA02	3.0	25Apr 2022	Hannah Scott	Detail added in reference to an online pre-screening questionnaire.<br>Revision of Day 14 window from ±21 to -5 to +21.
SA01	2.0	24 Jan 2022	Dr Hazel Morrison, Dr Susan Jackson, Dr Ingrid Cabrera Puig, Dr Rebecca Powell Doherty	Change to study dates due to effect of COVID pandemic on starting TB044. Change to study personnel and revision to funders.<br>Change to sponsor name and monitor.<br><br>Addition of details on adaptations made to undertake study during COVID pandemic throughout the document including:<br>- Inclusion of SARS-CoV-2 tests prior to enrolment, bronchoscopy and induced sputum as required during COVID-19 pandemic.<br>- Update of exclusion criteria to include confirmed or strongly suspected COVID-19 disease in last 3 months<br>- Addition of positive SARS-CoV-2 test to challenge and bronchoscopy postponement criteria.<br>- Addition of COVID-19 study specific self-isolation guidance (Risk assessment)<br>- Removal of routine spirometry after Day 7 visit.<br><br>Addition throughout document on use of adapted disposable duckbill mask and update of exclusion criteria to include allergy to any study procedure<br><br>Update to details on previous clinical experience as we have enrolled further participants into TB043.<br><br>Changes to study windows for later scheduled visits (day 28 onwards) which allow larger windows for participant visits

				during COVID 19 pandemic (in case of self-isolation requirements) without affecting scientific integrity of study.<br><br>Inclusion of possible induced sputum procedure at day 28 and 6 month visits. Including details on use of hypertonic saline and salbutamol.<br><br>Addition of BMI (BMI<18.5and >45) and previous receipt of experimental TB vaccine to exclusion criteria<br><br>Change to enrolment schedule to allow up to three participants at each dose to be enrolled during the same schedule and for dose escalation review to take place at 7 days, given availability of new safety data. Details of safety data sharing with funders<br><br>Change to immunology blood volumes at day 2, to allow additional immunology looking at early systemic immune responses in previously BCG-vaccinated volunteers<br><br>Addition of reference to BCGatlas.com<br><br>BCG vaccination to be at least 12 month prior to enrolment<br><br>Change of terminology from female to women of child bearing potential in relation to pregnancy testing and contraception
N/A	1.1	7th June 2020	Dr Ingrid Cabrera Puig	Clarifications to funding from both NIH/NIAID and The Wellcome Trust. Included as part of the response package to REC/HRA conditions

2. SYNOPSIS

**Title**	A clinical challenge study to evaluate controlled human infection with BCG administered by the aerosol inhaled route in historically BCG-vaccinated healthy adult volunteers
**Study Identifier**	TB044
**Study Centres**	Centre for Clinical Vaccinology & Tropical Medicine (CCVTM), University of Oxford, Churchill Hospital, Old Road, Headington, Oxford, OX3 7LE<br>Oxford University Hospital NHS Foundation Trust (OUH), Headington, Oxford, OX3 7LE
**Clinical Phase**	Phase I
**Design**	Dose escalation clinical challenge study
**Population**	Healthy adult volunteers aged 18-50 years
**Planned Sample Size**	12 participants<br>Group 1: 3 historically BCG-vaccinated volunteers will receive 1 x 10^4 cfu aerosol inhaled BCG<br>Group 2: 3 historically BCG-vaccinated volunteers will receive 1 x 10^5 cfu aerosol inhaled BCG<br>Group 3: 3 historically BCG-vaccinated volunteers will receive 1 x 10^6 cfu aerosol inhaled BCG<br>Group 4: 3 historically BCG-vaccinated volunteers will receive 1 x 10^7 cfu aerosol inhaled BCG
**Visit Schedule**	Screening followed by 8-12 visits over six months:<br>D0 (BCG challenge), D2, D7, D14 (Bronchoscopy), D28, D56, D84 & D168<br>(plus up to four brief SARS-CoV-2 testing visits)
**Planned Study Duration**	18 months (March 2022- Sept 2023)

	Objective	Outcome Measure
**Primary**	To quantify the BCG recoverable from bronchial samples in healthy historically BCG-vaccinated adult volunteers	BCG culture by mycobacterial growth indicator tube; PCR quantification and DNA strip assay speciation as required
**Secondary**	To evaluate the human clinical response to BCG challenge by the aerosol inhaled route in healthy, historically BCG-vaccinated adult volunteers	Actively and passively collected data on adverse events; detailed participant symptom profiles, lung function test results.
**Exploratory**	To identify laboratory markers of the immune response that correlate with the levels of BCG recovered from bronchial and other samples	Laboratory markers of innate and adaptive immunity, which may include ex-vivo ELISpot and ELISAs; RNA sequence analysis and intracellular cytokine staining of blood and bronchial samples. BCG CFU counts, MGIT and/ or molecular diagnostics on induced sputum (if performed) and on matrix from adapted mask collection
	Sample analysis for the completion of exploratory endpoints may be performed under the OVC Biobank research tissue bank protocol (REC: 16/SC/0141)	

**Challenge agent**	BCG
**Formulation**	Lyophilized, single vial
**Route of Administration**	Aerosol inhalation via nebuliser
**Dose per Administration**	1 x 10^4, 1 x 10^5, 1 x 10^6 and 1 x 10^7 cfu BCG

Lay Summary

Tuberculosis (TB) remains one of the deadliest infectious disease killers worldwide. Key research priorities include the development of an effective vaccine.

Currently, the only licensed vaccine against TB is the BCG (Bacille Calmette-Guérin). This works well against TB in childhood but is often ineffective in adults. Developing a new TB vaccine is difficult, as it is hard to determine which will be effective. In other diseases, e.g. influenza or malaria, it is possible to experimentally-infect volunteers with the disease to see if the proposed vaccine is effective. This is called a “controlled human challenge or infection model” and is possible in easily treatable or self-limiting diseases. This is not possible with TB, where treatments may be harmful and complex. Using a related but less infectious bacterium is a feasible alternative.

The BCG is a live attenuated (weakened) strain of the bacteria that causes TB in cattle. The BCG bacteria do not cause disease in healthy individuals. As the BCG and TB bacteria are similar, we are developing a challenge model using the BCG as an infectious agent to mimic TB infection.

Mycobacterium tuberculosis, the bacterium that causes TB, infects people by inhalation into the lungs. Therefore, inhaled BCG more closely imitates TB infection than an injection. A previous (TB041) and current study (TB043) in our group use aerosol inhaled BCG, in volunteers who have not received the standard BCG vaccination before.

The purpose of this study is to evaluate the human clinical response to BCG challenge in people who have previously been vaccinated with BCG. It will involve 12 participants in a controlled dose escalation. We will perform bronchoscopies 14 days post-challenge to measure BCG recovered from bronchial samples. We will take blood tests to look at potential immunological markers of immunity. We may also collect further respiratory samples at other time points, via adapted face masks (to capture respiratory droplets) and/or inhaled salty water to provoke a cough and production of phlegm (termed “induced sputum”).

3. ABBREVIATIONS

**AE**	Adverse event
**AID**	Autoimmune disease
**APTT**	Activated partial thromboplastin time
**BCG**	Bacillus Calmette–Guérin
**BALF**	Bronchoalveolar lavage fluid
**BALT**	Bronchus associated lymphoid tissue
**BCC**	Basal cell carcinoma
**CCVTM**	Centre for Clinical Vaccinology and Tropical Medicine, Oxford
**CBF**	Clinical Bio Manufacturing Facility
**CFU**	Colony forming unit
**CHIM**	Controlled human infection model
**CI**	Confidence interval
**CIS**	Carcinoma in situ
**COP**	Code of practice
**CRF**	Case report form or Clinical research facility
**CTL**	Cytotoxic T lymphocyte
**DSUR**	Development safety update report
**ELISPOT**	Enzyme-linked immunospot
**GCP**	Good Clinical Practice
**GIA**	Growth inhibition assays
**GMO**	Genetically modified organism
**GP**	General Practitioner
**HCG**	Human chorionic gonadotrophin
**HBV**	Hepatitis B virus
**HCV**	Hepatitis C virus
**HIV**	Human immunodeficiency virus
**HLA**	Human leukocyte antigen
**HRA**	Health Research Authority
**IB**	Investigator brochure
**ICH**	International Conference on Harmonisation
**ICS**	Intracellular Cytokine Staining
**ID**	Intradermal
**IGRA**	Interferon gamma release assay
**IFNγ**	Interferon gamma
**IM**	Intramuscular
**IV**	Intravenous
**MGIT**	Mycobacterial growth indicator tube
**M. tb**	Mycobacterium tuberculosis
**MVA**	Modified vaccinia virus Ankara
**NHS**	National Health Service
**NIAID**	National Institute of Allergy and Infectious Diseases
**NIH**	National Institutes of Health
**NIHR**	National Institute for Health Research
**PBMC**	Peripheral blood mononuclear cell
**PCR**	Polymerase chain reaction
**PI**	Principal investigator
**pfu**	Plaque forming unit

**PFTs**	Pulmonary function tests
**PVA**	Polyvinyl alcohol
**QP**	Qualified Person
**qPCR**	Quantitative polymerase chain reaction
**REC**	Research Ethics Committee
**RGEA**	Research Governance, Ethics and Assurance Team
**SAE**	Serious adverse event
**SC**	Subcutaneous
**SmPc**	Summary of product characteristics
**SOP**	Standard operating procedure
**SUSAR**	Suspected unexpected serious adverse reaction
**µg**	Microgram
**UKHSA**	UK Health Security Agency
**vp**	Viral particle
**VV**	Viral vector
**WHO**	World Health Organisation
**WOCBP**	Women of child bearing potential

4. BACKGROUND AND RATIONALE

4.1 Background

Mycobacterium tuberculosis (*M.tb*) is a pathogen with worldwide preponderance that infects humans and causes the transmissible disease tuberculosis (TB). An estimated one-third of the world's population is latently infected with *M.tb*, carrying a 10% lifetime risk of developing active life threatening disease (1). In 2018, there were 10 million new cases worldwide and 1.5 million people died of TB (2). Co-infection with human immunodeficiency virus (HIV) greatly increases the risk of TB reactivation and death (3, 4). Diagnosis is challenging and drug treatment is often harmful, costly and complex. For these reasons, it is essential to develop a more effective vaccine against TB.

The Bacille Calmette-Guérin (BCG) vaccine is the only licensed *M.tb* vaccine and has been administered globally to several billion people over a 90 year period, by the intradermal route (5). Although it is effective in preventing disseminated TB disease including tuberculous meningitis in childhood, it does not protect against pulmonary TB in endemic areas (4, 6, 7).

Recent advances in TB vaccine development have primarily been in the area of viral-vectored vaccines, given in prime-boost regimes with BCG as the priming vaccine. The most advanced of these vaccine candidates, MVA85A, had promising phase I results in the UK when given by the intradermal route, but subsequently showed significantly lower immunogenicity in phase II efficacy trials in South Africa (8, 9). The failure of the MVA85A vaccine to improve efficacy in BCG-vaccinated infants and adults highlights our inability to predict which candidate TB vaccines might work in humans. The predictive value of preclinical animal models remains uncertain, and we do not have a validated immunological correlate of protection with which to guide vaccine design and the selection of which candidate vaccines should progress to efficacy trials.

Historically, the main immunological readout for assessing immunogenicity in TB vaccine trials has been measurement of antigen specific interferon-gamma (IFN-γ) release by T cells in one of several immunological assays (10). There is considerable evidence to support the use of IFN-γ as an immunological readout (11-13), but whilst IFN-γ is essential for protective immunity against *M.tb*, it may not be sufficient. There are other cytokines, such as tissue necrosis factor alpha (TNFα), which are known to be important, as well as other functions of T cells, and antibodies, which may be involved (10, 11).

Challenge models

Currently, there is no reliable alternative to large, randomized controlled trials in order to assess vaccine efficacy against TB. These efficacy trials for novel TB vaccines are challenging, time consuming and very costly (14). The development of a safe controlled human mycobacterial challenge model which would ultimately be validated against field efficacy studies could greatly facilitate TB vaccine development. Such a model could be used both to guide vaccine selection and facilitate identification and validation of potential immunological correlates of protection.

In vaccine development for other pathogens such as malaria and para-typhoid, where animal models and immunological readouts are of uncertain or limited relevance, such challenge studies have been shown to be of great utility. So far it has not been possible to consider a human challenge approach in TB vaccine development because humans cannot be safely challenged with wild type virulent *M.tb*. Efforts to develop safe, attenuated strains of *M.tb*, with reporter genes to facilitate sensitive detection of changes in bacterial load, are progressing (15). In the meantime, however, progress with establishing basic parameters surrounding a clinical challenge model can be conducted using the existing TB vaccine, Bacille Calmette-Guérin (BCG), a live attenuated strain of *M. bovis*, which is licensed for human use. Using BCG as a model organism allows us to establish the optimal parameters for a human challenge model, which can then be applied to the challenge reporter strain when it becomes available.

4.2 Pre-Clinical Studies

In animal models using *M.tb* challenge, BCG confers a one to three log reduction in colony forming units (CFU) counts, or a significant improvement in survival or pathology scores (16). Prior BCG immunisation has been shown to protect against intradermal/intranasal BCG challenge in mice, non-human primate and cattle models, with reductions seen in quantity of recovered BCG as determined by culture CFU and quantitative PCR (17,18,19).

4.3 Previous clinical experience

Three studies to date have evaluated BCG as a human intradermal challenge agent. In this approach, a skin biopsy was taken two weeks after BCG 'challenge' and the mycobacteria in the biopsy were quantified by culture and PCR (20-22). This model demonstrated that viable BCG, detected using culture and PCR, was recoverable from a skin biopsy taken two weeks after BCG challenge. Furthermore we have demonstrated that prior BCG vaccination modulates mycobacterial recovery from subsequent BCG challenge (20, 21), in a population where BCG has previously been shown to protect against TB (23). We have also shown that the magnitude of the immune response correlates inversely with mycobacterial growth and have used the challenge model to identify mechanisms responsible for this response (24).

Basis for the aerosol inhaled route

The natural route of infection for *M.tb* is by inhalation of aerosolised infectious droplets containing tubercle bacilli, leading to the establishment of primary infection in the lung. The lung has a distinct mucosal immune system characterized by bronchus associated lymphoid tissue (BALT), which is well adapted to encounter and process antigens such as *M.tb*. Administering BCG challenge via the airway should therefore have the advantage over other routes, of more correctly reflecting the mucosal response to infection with *M.tb*, thus helping us to understand the evolution of early mycobacterial infection in the lung. A successful challenge model by this route will be of great value in testing the efficacy of future vaccines. The inhaled route is a well-established route of drug delivery. Aerosolised droplets of bronchodilating, anti-inflammatory, and antimicrobial drugs are administered by inhalation to millions of patients each day (25,26). In the 1970s, BCG was safely delivered by aerosol in a small study in healthy participants and in two studies in patients with lung cancer (27, 28).

We have established a new experimental medicine paradigm in which aerosolised BCG or candidate TB vaccines are delivered to the lungs. This is followed by bronchoscopic assessment of mucosal immunity (29). We have conducted over 150 bronchoscopies on healthy human participants, of whom 31 received aerosolised BCG in a dose-finding safety study. A further 42 have received aerosolised BCG as part of an ongoing study into innate and adaptive immunity following BCG challenge. All have been without clinically significant adverse events (clinicaltrials.gov NCT02709278, NCT01954563, NCT02532036 + NCT03912207). We are therefore well-placed to use state-of-the-art immuno-monitoring to dissect the human pulmonary innate and adaptive immune responses to aerosol BCG challenge and to develop a clinically utilisable mycobacterial challenge model.

We have previously demonstrated that prior BCG vaccination modulates mycobacterial recovery from subsequent intradermal BCG challenge (20, 21). This study will extend our knowledge by allowing us to study the immune response and BCG recovery at a lung mucosal level following an aerosol BCG challenge in previously BCG-vaccinated healthy UK adult volunteers - a population where BCG is known to be protective (23).

Aerosol delivery devices

The World Health Organisation (WHO) has made major investments in developing new aerosol devices, providing a portable, low cost method of vaccine delivery (30). These devices are small, lightweight and aerosolise through a mesh to provide small and consistent particle size for vaccine or challenge agent delivery to the distal airway mucosa more accurately and precisely than conventional jet mechanisms. Two such mesh nebuliser are the MicroAIR NE-U22 and NE-U100 (Omron® Healthcare Limited, Japan) which use ultrasound to push liquid through a fine metal mesh. This generates an aerosol mist with a particle diameter of about 4-4.5µm. They are in current use with licensed drugs such as bronchodilating and antimicrobial agents (e.g. salbutamol, ipratropium bromide and tobramycin) and achieve good bioavailability. BCG particles are typically around 2-4µm in size and can therefore be aerosolised with minimal damage (32-33). We have used the U22 device in our previous human aerosol TB vaccine clinical trials TB026, TB035 (29), Clinicaltrials.gov NCT01954563, NCT02709278) and our current study TB043 (NCT03912207) with a good usability and safety profile.

4.4 Rationale

Hypothesis

We believe that aerosol inhalation is a safe and practical route of BCG challenge in historically BCG-vaccinated healthy volunteers. We hypothesise that in future studies, prior BCG vaccination will protect against subsequent aerosol BCG challenge (as defined by in-vitro PBMC mycobacterial growth inhibition assays and quantification of BCG recovered from the lungs) allowing identification of immune correlates of protection.

BCG and dosing

Current dosing and strain

The strain of BCG used in this study will be BCG Danish 1331 (AJ vaccines - formerly SSI), which is the strain licensed for (intradermal) use in the UK.

The dose of inhaled BCG used in our current study TB043 (Clinicaltrials.gov NCT03912207) is the target (maximum) dose that was safely tolerated in our previous dose escalation study TB041 (NCT02709278). This target dose was calculated as follows. The intradermal licensed dose of BCG Danish 1331 for adults is 2-8 x 10^5 cfu in 0.1ml (34). We calculated the equivalent dose for aerosol BCG based upon an average of this: $5x10^5$ cfu in a 0.1ml adult standard dose, which translates into a BCG concentration of $5x10^6$ cfu/ml.

We aim to deliver this standard dose of $5x10^5$ cfu into the lung by loading $1x10^7$ cfu BCG into the nebuliser. This accounts for the losses of BCG due to method of delivery as follows:

1. The amount of viable BCG cfu recoverable from a vial of BCG may be lower than the expected amount based on the Summary of Product Characteristics (SmPC). The viable BCG cfu from the vials of BCG Danish 1331 AJV reconstituted in TB043 is shown below (Figure 1).

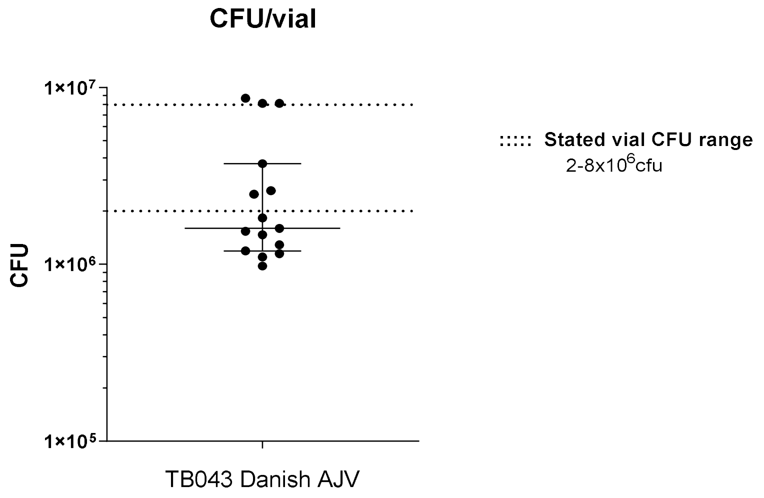

Figure 1. Viable BCG cfu from BCG Danish vials reconstituted in TB041

2. The vaccine vial is reconstituted to achieve the required dose and we have plated out these dilutions to obtain an estimate of the dose that is loaded onto the nebuliser (*Figure 2*). This loaded dose is consistently one-half to one log lower than our intended dose. Note BCG Bulgaria was utilised for part of TB041 due to shortage in supply of BCG Danish (SSI).

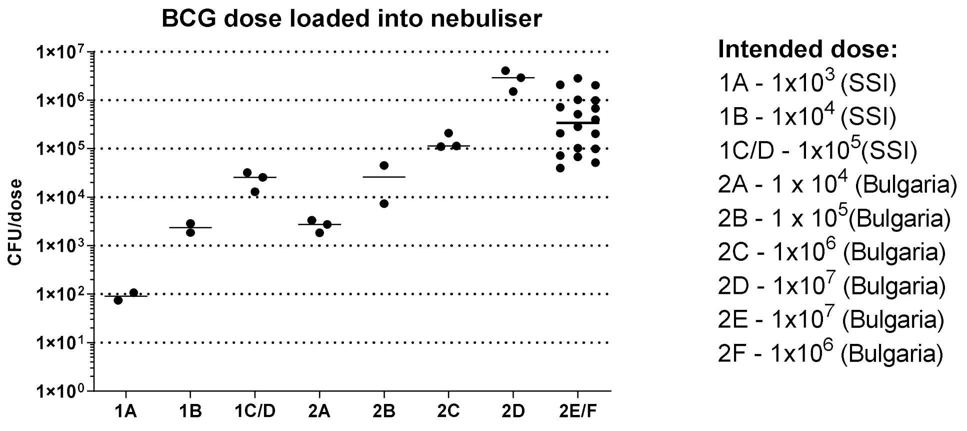

Figure 2. BCG cfu actual dose following reconstitution+/-dilution of either BCG SSI or BCG Bulgaria

3. We have developed an *in-vitro* model to quantify the actual delivered dose of nebulised BCG. This involved using a vacuum pump attached to an impinger to collect the aerosol expelled by the Omron nebuliser. The amount of BCG in this collected sample was quantified by culture plating. This model consistently measured an approximately 50% loss of BCG compared to the amount of BCG loaded into the nebulizer (*Figure 3*). This model does not take into account the further potential loss of BCG that occurs in a clinical setting through loss of aerosol into the environment, as the volunteer does not breathe in continuously, and the nebuliser continues to aerosolise the BCG between breaths.

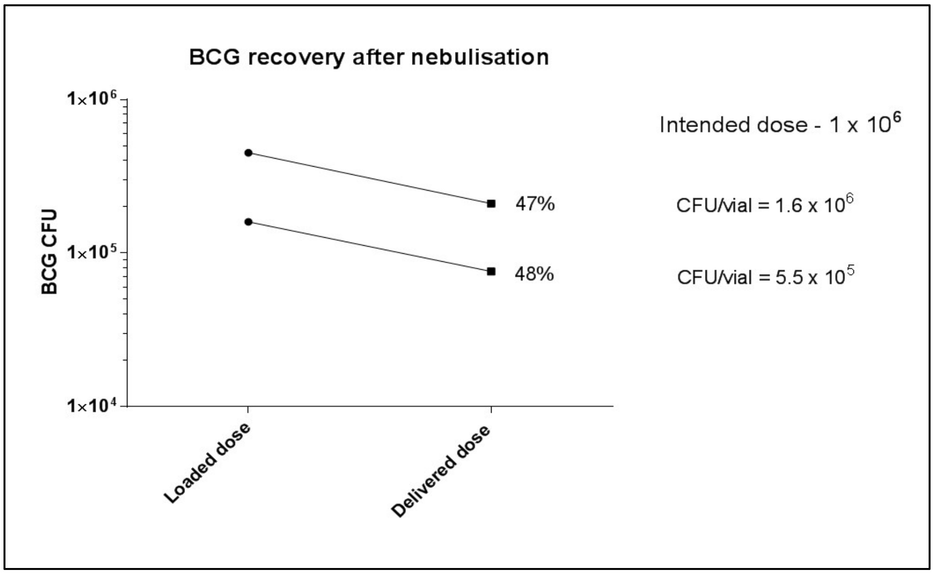

Figure 3. BCG recovery after nebulisation using vacuum model

Given these documented losses, we ensure the maximum dose delivered is $5x10^5$ cfu by utilising a prepared dose of $1x10^7$cfu. This dose was well tolerated by volunteers in our previous studies and was immunogenic.

Dose escalation and rationale

As documented above, $1x10^7$ cfu BCG dose will be the maximum targeted dose we would aim to deliver. As this is the first time we are delivering aerosol BCG to previously BCG-vaccinated volunteers, we plan to start at very low challenge doses with careful incremental dose increases. Doses used at each increment from $1x10^4$ are the same as our previous dose escalation study in BCG-naïve volunteers TB041 (NCT02709278). In TB041, 31 volunteers received BCG via the aerosol route, 12 at the highest dose of $1x10^7$ cfu. All have now completed their six-month follow up with no safety concerns. In our current study, TB043 (NCT03912207) 42 volunteers so far have received $1\ x10^7$cfu BCG with no safety concerns identified. Furthermore, a group in South Africa have recently instilled BCG directly into the lungs bronchoscopically (thus without the losses and subsequent lower actual delivered dose described above) at a dose of $1X10^4$ CFU in BCG-vaccinated and *M.tuberculosis* latently infected subjects and this was well tolerated, with no hypersensitivity reactions seen(47).

Due to a shortage in BCG Danish 1331 during the study, dose escalation above $1x10^5$cfu in TB041 was performed with BCG Bulgaria strain of BCG. Table 1 shows the strain and doses of aerosol inhaled BCG received by volunteers in our trials to date.

Trial	BCG strain and manufacturer	Dose (cfu)	Number of Volunteers
TB041	BCG Danish 1331 (SSI)	1×10^3	3
		1×10^4	3
		1×10^5	4
	BCG Bulgaria (Intervax)	1×10^4	3
		1×10^5	3
		1×10^6	3
		1×10^7	12
TB043	BCG Danish 1331 (AJ vaccines - formerly SSI)	1×10^7	42

Table 1. Strain and dose of aerosol inhaled BCG

Whilst numbers were small, no significant difference was seen in adverse events between the two strains of BCG administered in TB041 at the equivalent doses (see *figure 4*).

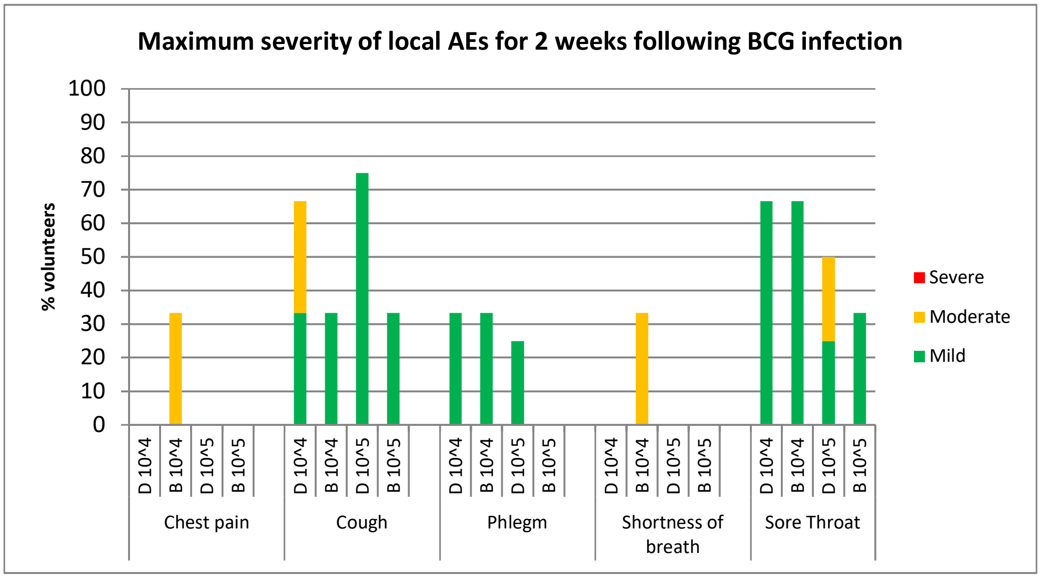

*no haemoptysis reported by any volunteer

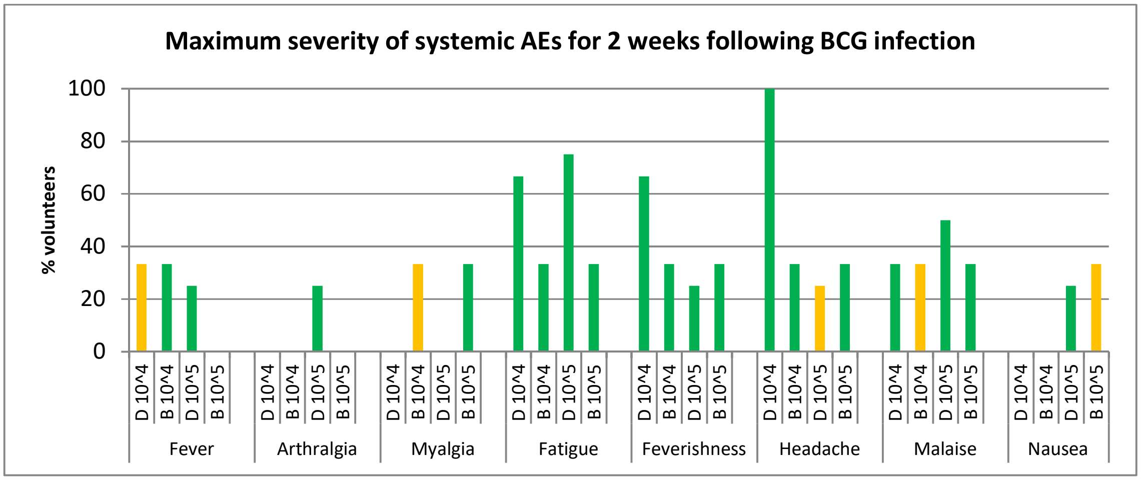

D = BCG Danish 1331, B = BCG Bulgaria

Figure 4. Comparison of AEs between different strains of aerosol inhaled BCG.

In this study, volunteers will receive BCG Danish 1331 (AJ vaccines - formerly SSI). Volunteers in group 1 will receive a very low dose of inhaled BCG – 1 x10^4 cfu. The first volunteer in each group will be challenged at least 1 hour apart from any subsequent volunteers. There will be a review of safety data at least 7 days after challenge of all volunteers in group 1. This safety review will be performed by the Safety Monitoring Committee (SMC). Further details of safety review and stopping rules are given in sections 6 and 10. As this is no longer a first in human study of pulmonary BCG in BCG-vaccinated individuals, and given the good safety prolife of bronchoscopic BCG given to BCG-vaccinated (and latently infected) South Africans (47), the requirement for the first volunteer in each group to be vaccinated on a separate day has been removed.

If considered safe to do so by the SMC, we will then dose escalate to 1 x 10^5 cfu in the same manner. There will be a further safety review by the SMC at least 7 days after challenge of all volunteers in group 2. If considered safe to do so, groups 3 and 4 will proceed in the same manner, with doses of 1 x 10^6 and 1 x 10^7 cfu respectively.

Contribution to overall strategy for TB vaccine development

The ability to measure and quantify growth inhibition of BCG following a human challenge could provide a functional measure of anti-mycobacterial immunity induced by vaccination. This may be used to determine if potential biomarkers in peripheral blood that can be correlated with BCG growth inhibition. The development and validation of a controlled human infection model (CHIM) for TB would provide a significant new tool in the armoury of rational TB vaccine development.

The exploratory end-points of this study will contribute to a better understanding of the innate and adaptive immune response, both at a local respiratory mucosal level and systemically, following an acute *in-vivo* human mycobacterial challenge. A greater understanding of mucosal innate and adaptive immune constituents could help to develop interventions aimed at halting *M.tb* infection at the point of entry. Knowledge of this mucosal adaptive immune response may aid in vaccine development, as a successful vaccine candidate may alter or augment the immune response differently at a local level when compared with peripheral blood.

4.5 Face-mask sampling

We are collaborating with Professor Barer's team from the University of Leicester who have developed an adapted duckbill face mask containing a collection matrix. The matrix consists of strips of Polyvinyl Alcohol (PVA) for the purpose of collecting exhaled bacteria. A proof of concept case finding study in South Africa demonstrated that this PVA matrix technology detected pulmonary tuberculosis with a greater sensitivity than a contemporaneous sputum sample when performed at one time-point in symptomatic individuals and also appeared to detect disease at an earlier stage. (50) It would be an unfair burden to perform repeated bronchoscopies on individuals in an aerosol CHIM, however, collecting BCG recovery data at different time-points in an individual would allow further evaluation of the human clinical response to BCG. Assessing this method for BCG recovery against both BAL and induced sputum recovery of BCG could potentially facilitate an easier process for BCG recovery in future aerosol BCG CHIMs.

4.6 BCG Challenge Study and COVID-19 Pandemic

In December 2019, a cluster of severe pneumonia cases of unknown aetiology was identified in Wuhan, China and was later confirmed to be due to a novel betacoronavirus. The virus was subsequently named SARS-CoV-2 due to its genetic similarity to the coronavirus responsible for severe acute respiratory syndrome (SARS-CoV). By January 2020, there was increasing evidence of human to human transmission and the number of cases of the new coronavirus disease, termed COVID-19, rapidly spread across the world. The World Health Organisation declared the outbreak of COVID-19 a pandemic on 11 March 2020 (43). As of 17th September 2021, there have been 7,406,017 confirmed COVID-19 cases and 135,314 deaths from COVID-19 in the United Kingdom(44).

Due to the aerosol route of delivery and need for respiratory monitoring and sampling, additional precautions will be implemented in this trial during the current COVID-19 outbreak.

There is currently very limited evidence available related to BCG and SARS-CoV-2 interaction. There is some interest in the potential non-specific innate immune system-boosting effects of BCG as a mechanism to reduce COVID-19 disease, but no current conclusive evidence and all ongoing studies into this effect are using ID BCG. Due to the lack of evidence and unknown interactions between BCG and SARS-CoV-2, we will exclude any volunteers who have had a laboratory confirmed (PCR or antibody) SARS-CoV-2 infection, evidence of current viral pneumonitis on chest radiograph or a high clinical suspicion of COVID-19 disease in the 3 months preceding enrolment.

During the pandemic SARS-CoV-2 testing will be undertaken 2 days (+/- 2 days) prior to enrolment and any participant who has a positive test would not be enrolled for at least 3 months. SARS-CoV-2 testing will also occur 2 days (+/- 2 days) prior to bronchoscopy. If positive, bronchoscopy will be postponed by at least 28 days from onset of symptoms (or test date if asymptomatic) as per British Thoracic Society guidelines (45). If this is not possible, the volunteer will not undergo bronchoscopy but may continue with other follow up visits at the discretion of the investigator once their self-isolation period has been completed. Immunology bloods tests will not be taken for 14 days from the onset of symptoms (or test date if asymptomatic) to minimise any infection risk.

Spirometry as a measure of pulmonary function will be routinely monitored at screening, day 0, day 2 and day 7, with additional transfer factor measured at screening and day 7.

If there is a significant drop in transfer factor (≥15% from baseline or fall to below 80% predicted) (46), then this will be repeated at 3 months +/- 6 months if needed. Staff members will wear appropriate personal protective equipment (PPE). There has been no evidence of early or late allergic response to inhaled BCG in any of our trials and there has been no clinically significant sustained decrease in any spirometry parameters seen in any volunteer. Although, this is our first trial to administer aerosolized BCG to previously BCG vaccinated volunteers, a group in South Africa have recently administered 10^4 CFU BCG per bronchoscope in BCG-vaccinated and *M. Tuberculosis* latently infected subjects (47). This was well tolerated with no participants developing a significant adverse event or showing evidence of hypersensitivity reactions. Given this and the aerosol generating nature of the procedure, routine spirometry at all other visits will not occur. Spirometry may still be performed at any visit if at clinical discretion it is felt to be indicated and will occur using appropriate PPE. Induced sputum procedures are also aerosol generating. SARS-CoV-2 testing may be undertaken 2 days (+/- 2 days) prior to induced sputum procedures if required.

Any volunteers who test positive for current SARS-CoV-2 infection will be required to self-isolate as per the latest public health guidelines. As required by law, all results (whether positive or negative) and personal data (including volunteer name, contact details and postcode) will be shared with UK HSA. Asymptomatic volunteers undergoing planned SARS-CoV-2 testing will not be required to isolate whilst awaiting their results. All adaptations are in line with current UK government advice and may be modified in line with this advice if it changes in future.

If a volunteer is required to self-isolate, either due to their own symptoms/positive test or due to contact with a positive case, any routine study visits that cannot be postponed until after the isolation period will be conducted as far as possible by telephone/video call during the study window visit. A protocol deviation will still be completed for missed study samples. If safety bloods are due or there are any clinical concerns, a face-to-face visit may still be conducted at the discretion of the investigator.

5. OBJECTIVES AND ENDPOINTS

The number of volunteers in this study has been chosen to generate sufficient data to meet the objectives described below, whilst minimising the number of volunteers exposed to BCG challenge.
Sample analysis for the completion of exploratory endpoints may be performed under the ethically approved OVC Biobank protocol.

Objectives	Outcome Measures	Time point
**Primary**<br>To quantify the BCG recoverable from bronchial samples in healthy historically BCG-vaccinated adult volunteers	BCG culture by CFU counts and mycobacterial growth indicator tube; PCR quantification and DNA strip assay speciation	Follow up visit at D14
**Secondary**<br>To evaluate the human clinical response to BCG challenge by the aerosol inhaled route in healthy, historically BCG-vaccinated UK adult volunteers	Actively and passively collected data on adverse events; detailed participant symptom profiles, lung function test results	Adverse events collected at each visit and via diary card.<br>Safety blood tests at baseline, day 7, 14 and 28.<br>Lung function at day 0 post challenge, day 2 and 7 and later time points if clinically indicated
**Exploratory**<br><br>To identify laboratory markers of the immune response that correlate with the levels of BCG recovered from bronchial samples	Laboratory markers of innate and adaptive immunity, which may include ex-vivo ELISpot and ELISAs; RNA sequence analysis and intracellular cytokine staining of blood and bronchial samples. BCG CFU counts, MGIT and/ or molecular diagnostics on induced sputum and on PVA matrix samples from duckbill face mask collection	Bronchial samples at D14<br>Bloods at each follow up visit<br>Induced sputum at D28 and D168 (not during peak of COVID pandemic).<br>Mask wearing at D0 (pre and post enrolment), D2, D7, D14, D28 and D168.

6. STUDY DESIGN

This is a phase I dose-escalation clinical challenge study in historically BCG-vaccinated healthy adult volunteers in which BCG will be administered via the aerosol inhaled route.

There will be four study groups with three volunteers in each group (Table 2). Volunteers will be sequentially enrolled into each group in turn based on their availability. The first volunteer in each group will be challenged at least 1 hour apart from any subsequent volunteers. There will be a review of safety data at least 7 days after challenge of all volunteers within each dose group. This safety review will be performed by the Safety Monitoring Committee (SMC). Dose escalation into the next group will only occur if there are no safety concerns. Safety review will include assessment of symptom diary card, safety bloods and lung function results.
.

6.1 Study groups

Volunteers will be recruited and challenged at the CCTVM, Oxford. A total of 12 volunteers will be enrolled in this study (Table 2).

Group	Number of Volunteers	Dose	Route
1	3	1 x 10^4 cfu	aerosol inhaled BCG
2	3	1 x 10^5 cfu	aerosol inhaled BCG
3	3	1 x 10^6 cfu	aerosol inhaled BCG
4	3	1 x 10^7 cfu	aerosol inhaled BCG

Table 2. Study groups.

6.2 Study volunteers

Study population will be healthy male and non-pregnant females between 18 and 50 years old who have previously received the BCG vaccine at least 12 months prior to enrolment, as verified by scar or medical (or occupational health) records. Eligibility will depend on review of medical and social history, physical exam and the results of laboratory tests. Volunteers will be considered enrolled immediately after receiving the BCG challenge.

6.3 Definition of Start and End of Study

The start of the study is defined as the date the first volunteer is recruited into the study (i.e. the date the first volunteer provides informed consent). The end of the study is the date of the last visit of the last volunteer.

Samples will be retained for a maximum of one year from the end of the study (last patient, last visit) to allow for analysis of protocol-defined laboratory endpoints relating to primary and secondary objectives. Following this period, samples will be transferred to the OVC biobank if consent has been obtained for this.

6.4 Potential Risks for volunteers

The potential risks to participants in this study are those associated with BCG challenge, bronchoscopy, induced sputum procedure, venepuncture, intravenous cannulation, chest radiography and lung function manoeuvres.

6.4.1 BCG challenge

BCG Danish 1331 is on the WHO list of pre-qualified vaccines and has a well-defined side effect profile. Full details are given in the SmPC (34). BCG is licensed for delivery via the intradermal route but it is not licensed for delivery via the aerosol route.

Anticipated local adverse events following aerosol challenge include mild throat discomfort and cough. Transient feelings of shortness of breath, wheeze, chest tightness or chest pain are also possible. Systemic reactions to aerosolised BCG may include transient fever or feverishness, malaise, nausea, headache, myalgia, arthralgia or fatigue. In the aerosol BCG trial TB041 (Clinicaltrials.gov NCT02709278), approximately 1/2 of participants experienced systemic symptoms, with the majority being mild (grade 1) and lasting an average duration of around 24 hours.

Following intradermal BCG, disseminated complications of BCG, such as bone infections, have been reported, but are extremely rare and usually reported in immunocompromised individuals. No such complications have occurred in any trial delivering aerosolised BCG. Volunteers are screened to ensure they are immunocompetent prior to enrolment. BCG Danish 1331 is fully susceptible to isoniazid, rifampicin, ethambutol and all second line anti-tuberculous drugs. As with all *M. bovis* derived strains, BCG is inherently resistant to pyrazinamide.

Allergic reactions from mild to severe may occur in response to any constituent of a medicinal product's preparation. Anaphylaxis is extremely rare (<1/1000 people) but can occur in response to any vaccine/ medication.

6.4.2 Bronchoscopy

Bronchoscopy is a widely and safely used investigative procedure in clinical research studies involving both healthy volunteers and patients with respiratory conditions such as asthma and interstitial lung disease (35, 36). Clinical guidelines for performing investigative bronchoscopy in research studies are well established (37).

The bronchoscopies will be carried out in a dedicated NHS bronchoscopy suite with an excellent safety record by highly skilled and experienced consultant respiratory physicians. Intravenous sedation and topical local anaesthesia are administered prior to bronchoscopy to reduce discomfort, facilitate the procedure, and remove memory of the event. Trained, experienced staff and facilities for resuscitation and drugs for reversal of sedation will be available.

The risks of bronchoscopy are discussed at the time of initial consent and again prior to the procedure itself. Risks comprise: adverse reaction to sedation or local anaesthetic, sore throat and /or transient hoarse voice, laryngospasm/bronchospasm, hypoxia, infection, post-procedure flu-like symptoms (1-2 days), air leak (pneumothorax) requiring chest tube insertion (≤0.1%), bleeding requiring treatment (≤0.5%), low blood pressure, abnormal heart rhythm (≤0.1%) and risk of death (<0.02%). No transbronchial biopsies will be taken to minimise the risk of pneumothorax.

To further minimise risk in general, volunteers will be excluded if they have an abnormal chest radiograph, increased bleeding risk, a significant smoking history, a history of severe atopy or any evidence of lung disease, including asthma (as defined by: a confirmed clinical diagnosis of asthma; recurrent prescription of asthma medication; airflow obstruction on spirometry; history of nocturnal or exercise-induced wheeze).

Bronchoalveolar lavage is a routine procedure in investigative bronchoscopy. The risk of infection or febrile reactions will be minimised by appropriate bronchoscope sterilisation. Respiratory depression secondary to sedation is rare. No rescue medication for over sedation has been required for volunteers participating in our aerosol clinical trials (TB026, TB035, TB041 or TB043) to date. Allergic reactions from mild to severe may occur in response to any constituent of the local anaesthetic or sedative agents. Anaphylaxis is extremely rare but can occur. The Summary of Product Characteristics (SmPC) for the local anaesthetic and sedative agents contain full details of the indications and side effects of these licensed medications.

6.4.3 Venepuncture and intravenous cannulation

Localised bruising and discomfort can occur at the site of venepuncture. Infrequently fainting may occur. Very rarely soft tissue infection may occur. The total volume of blood drawn over a six month period will be 523mL. This should not compromise these otherwise healthy volunteers, as they would donate 470mL during a single blood donation for the National Blood transfusion Service over a 3-4 month period. Volunteers will be asked to refrain from blood donation for the duration of their involvement in the study.

An intravenous cannula is routinely inserted into a peripheral (usually forearm) vein prior to bronchoscopy in order to administer intravenous sedation. This it is removed once the procedure is completed. The risks of cannulation are identical to those associated with venepuncture but include an additional small risk of soft tissue infection. This risk will be minimised by an aseptic insertion technique and is easily recognisable and treatable. The short duration of cannulation (a few hours) further minimizes this risk. Additional rare risks associated with intravenous cannulation include superficial phlebitis/thrombophlebitis, air embolism, extravasation/infiltration and intra-arterial injection.

6.4.4 Chest radiograph

A chest radiograph is a painless radiological investigation that exposes volunteers to approximately 0.02 milliSieverts of radiation, equivalent to around 3 days of natural background radiation. The additional risk of cancer due to one chest radiograph is insignificant (1/900,000).

6.4.5 Spirometry and Transfer factor of the Lung for Carbon Monoxide (TLCO)

Vigorous respiratory manoeuvres such as forced expiration through the spirometer can occasionally lead to coughing or light-headedness, but these symptoms are mild and rapidly self-limiting.

6.4.6 SARS-CoV-2 testing

This is currently most widely performed via a combined oropharyngeal and nasopharyngeal swab for SARS-CoV-2 PCR. It is not painful but can be uncomfortable. Swabbing the back of the throat can cause individuals to cough or gag. The deep nasal swab can cause eye watering or rarely cause nose bleeding. Lateral flow testing may be utilised instead of PCR testing prior to induced sputum procedures, which take place in a negative pressure isolation tent. If lateral flow test is positive, the visit would not proceed and the volunteer would be advised to follow current government guidelines.

6.4.7 Mask wearing

Volunteers will be asked to wear an adapted disposable duckbill mask for 30 minutes prior to and following Day 0 challenge, for 30 minutes immediately prior to bronchoscopy (Day 14), and for 30 minutes at the Day 2, Day 7, Day 28 and 6 month follow-up visits. The mask is fitted with a PVA collection matrix that captures aerosolized droplets from the lungs. Analysing the matrix will allow us to compare bacterial recovery at baseline and over time. There are no anticipated risks associated with mask wearing although it may not be suitable for individuals who suffer from claustrophobia. History of claustrophobia will be taken at screening visits.

6.4.8 Induced Sputum

Inhalation of hypertonic saline to induce sputum production can occasionally result in excessive bouts of coughing or sensation of shortness of breath but these symptoms are mild and rapidly self-limiting. Rarely, hypertonic saline can induce bronchoconstriction but this is very unlikely to occur in our cohort of patients who have no clinically significant history of atopy and no asthma. Hypertonic saline-induced bronchospasm is quickly reversed by treatment with an inhaled short-acting B_2 agonist. Volunteers will be pre-treated with inhaled salbutamol and their lung function will be monitored throughout the procedure. This procedure has been used in TB041 and TB043 (Clinicaltrials.gov NCT02709278 and NCT03912207) with no adverse events and has been well tolerated by volunteers.

6.5 Known Potential Benefits

Volunteers are not expected to benefit directly from participation in this study. Volunteers will gain some information about their general health as a result of the screening, examination, blood tests, urine tests, chest radiograph or pulmonary function. They may also gain health information from the bronchoscopy. Volunteers found to have a previously undiagnosed condition thought to require further medical attention will be referred appropriately to their GP or an NHS specialist service for further investigation and treatment, with their permission.

It is hoped that their contribution will further the development of a safe and successful vaccine for TB and our knowledge about TB infection and protection.

7. RECRUITMENT AND WITHDRAWAL OF STUDY VOLUNTEERS

7.1 Identification of Study Volunteers

Healthy adults aged 18-50 years who have previously received the BCG vaccine will be recruited by use of an advertisement +/- registration form formally approved by the ethics committee(s) and distributed or posted in the following places

- In public places, including buses and trains, with the agreement of the owner / proprietor
- In newspapers or other literature for circulation
- On radio via announcements
- On a website or social media site operated by our group or with the agreement of the owner or operator (including on-line recruitment through our web-site)
- By e-mail distribution to a group or list only with the express agreement of the network administrator or with equivalent authorisation
- By email distribution to individuals who have already expressed an interest in taking part in any clinical trial at the Oxford Vaccine Centre
- On stalls or stands at exhibitions or fairs

- Via presentations (e.g. presentations at lectures or invited seminars)
- Direct mail-out: This will involve obtaining names and addresses of adults via the most recent Electoral Roll. The contact details of individuals who have indicated that they do not wish to receive postal mail-shots would be removed prior to the investigators being given this information. The company providing this service is registered under the Data Protection Act 2018. Investigators would not be given dates of birth or ages of individuals but the list supplied would only contain names of those aged between 18-50 years (as per the inclusion criteria)
- Oxford Vaccine Centre databases: We may contact individuals from databases within the CCVTM (including the Oxford Vaccine Centre database) of previous trial participants who have expressed an interest in receiving information about all future studies for which they may be eligible
- Prior to invitation for a screening visit, individuals will be directed to an online pre-screening questionnaire covering key eligibility criteria such as age. Respondents that are potentially eligible at this stage will be invited to the trial site for the screening visit.
- Potential volunteers who express an interest in the study may also be contacted by phone or email by a member of the study team (clinician or nurse) prior to being formally invited for a screening visit.

7.2 Informed consent

All volunteers will sign and date the informed consent form before any study specific procedures are performed. The information sheet will be made available to the volunteer at least 24 hours prior to the screening visit. At the screening visit, the volunteers will be fully informed of all aspects of the study, the potential risks and their obligations. The following general principles will be emphasised:

- Participation in the study is entirely voluntary
- Refusal to participate involves no penalty or loss of medical benefits
- The volunteer may withdraw from the study at any time
- The volunteer is free to ask questions at any time to allow him or her to understand the purpose of the study and the procedures involved
- There is no direct benefit to the volunteer from participating
- The volunteer will be registered on the TOPS database (The Over-volunteering Prevention System; www.tops.org.uk)
- The volunteer's GP will be contacted to corroborate their medical history. Volunteers will only be enrolled in the study if written or verbal information regarding the volunteer's medical history is obtained from the GP. This can either be via the study team accessing patient's electronic care summaries from local systems, by contacting the GP or volunteers bringing their medical care summaries from the GP to the study clinicians
- If agreed to, samples taken as part of the study may be sent outside of the UK and Europe to laboratories in collaboration with the University of Oxford. These will be anonymised. Volunteers will undergo a separate, optional consent process (under the Oxford Vaccine Centre Biobank protocol) regarding future storage of any leftover samples for use in other ethically approved research.
- The aims of the study and all tests to be carried out will be explained. The volunteer will be given the opportunity to ask about details of the study, and will then have time to consider whether or not to participate. If they do decide to participate, they, and the investigator, will sign and date the consent form. The volunteer will then be provided with a copy of the consent form to take away and keep, with the original being stored in the case report form (CRF).

7.3 Inclusion and exclusion criteria

This study will be conducted in healthy adults, who meet the following inclusion and exclusion criteria:

7.3.1 Inclusion Criteria

The volunteer must satisfy all the following criteria to be eligible for the study:

- Healthy adults aged 18-50 years
- Previously vaccinated with the BCG (at least 12 months prior to enrolment, as evidenced by a visible scar or documentation in medical or occupational health records)
- Resident in or near Oxford for the duration of the study period
- Provide written informed consent
- Willing to allow the investigators to discuss the volunteer's medical history with their General Practitioner (or review summary care record, if available)
- Allow the investigator to register volunteer details with a confidential database (The Over-volunteering Protection Service) to prevent concurrent entry into clinical studies/trials
- Agreement to refrain from blood donation during the course of the study
- For women of child-bearing potential* only, willingness to practice continuous effective contraception (see below) during the study and a negative pregnancy test on the day(s) of screening, challenge and bronchoscopy
- Able and willing (in the investigator's opinion) to comply with all study requirements
- No clinically relevant findings in medical history or on physical examination
- Screening IGRA negative
- Willing to be tested for evidence of SARS-CoV-2 infection and to allow public health notification of the results*For the purpose of this document, a woman is considered of childbearing potential (WOCBP), i.e. fertile, following menarche and until becoming post-menopausal unless permanently sterile. Permanent sterilisation methods include hysterectomy, bilateral salpingectomy and bilateral oophorectomy. A postmenopausal state is defined as no menses for 12 months without an alternative medical cause.

7.3.2 Exclusion Criteria

The volunteer may not enter the study if any of the following apply:

- Any clinically significant respiratory disease, including asthma
- Current smoker (defined as any smoking including e-cigarettes in the last 3 months)
- History of anaphylaxis to vaccination or any allergy likely to be exacerbated by any component of the study agent, any essential study procedure, sedative drugs, or any local or general anaesthetic agents
- Clinically significant history of skin disorder, allergy, atopy, cancer (except BCC of the skin or CIS of the cervix), cardiovascular disease, gastrointestinal disease, liver disease, renal disease, endocrine disorder, neurological illness, psychiatric disorder, drug or alcohol abuse, or any autoimmune conditions or immunodeficiency (including HIV)

- Previous diagnosis or treatment for TB disease or latent TB infections
- Clinical, radiological, or laboratory evidence of current active TB disease or latent TB infection
- Previous receipt of any investigational TB vaccine
- Positive HBsAg, HCV or HIV antibodies
- Concurrent use of oral, inhaled or systemic steroid medication or use for more than 14 days within the last 6 months (steroids used as a cream or ointment are permissible), or the use of other immunosuppressive agents concurrently or for more than 14 days within the last 6 months
- Administration of immunoglobulins and/or any blood products within the three months preceding the planned study challenge date
- Current use of any medication or other drug taken through the nasal or inhaled route including cocaine or other recreational drugs
- Any nasal, pharyngeal, or laryngeal finding which precludes bronchoscopy
- Pregnancy, lactation or intention to become pregnant during study period
- Previously resident for more than 12 months concurrently in a tropical climate where significant non-tuberculous mycobacterial exposure is likely
- Shares a household with someone with clinically significant immunodeficiency (either from infection or medication) who is deemed to be at risk of developing disseminated BCG infection if exposed to BCG
- Participation in another research study involving receipt of an investigational product in the 30 days preceding enrolment, or planned use during the study period
- Clinically significant abnormality on screening chest radiograph
- Clinically significant abnormality of lung function testing
- Any clinically significant abnormality of screening blood or urine tests
- Laboratory confirmed (PCR or antibody) SARS-Cov-2 infection, evidence of viral pneumonitis on chest radiograph or a high clinical suspicion of COVID-19 disease in the 3 months preceding enrolment.
- A body mass index (BMI) of <18.5 or >45
- Any other significant disease, disorder, or finding, which, in the opinion of the investigator, may either put the volunteer at risk, affect the volunteer’s ability to participate in the study or impair interpretation of the study data

Volunteers who are excluded from the study because they have been discovered during screening procedures to be suffering from a previously undiagnosed condition thought to require further medical attention will, with their consent, be referred appropriately to their GP or an NHS specialist service for further investigation and treatment.

7.3.3 Effective contraception for female volunteers

Women of child-bearing potential are required to use an effective form of contraception during the course of the study. Acceptable forms of contraception include:

- Established use of oral, injected or implanted hormonal methods of contraception
- Placement of an intrauterine device (IUD) or intrauterine system (IUS)
- Barrier methods of contraception (condom or occlusive cap with spermicide)
- Male sterilisation, if the vasectomised partner is the sole partner for the participant
- Same sex intercourse only

True abstinence, when this is in line with the preferred and usual lifestyle of the participant (Periodic abstinence and withdrawal are not acceptable methods of contraception)

7.3.4 Temporary Exclusion Criteria

Challenge Postponement Criteria

Challenge will not proceed on the scheduled day in any of the following situations:

- The volunteer has a current or recent upper respiratory tract infection, unless they have been symptom-free for at least one week
- The volunteer has a temperature $\geq$ 37.8°C
- The investigator judges the volunteer to have an acute moderate or severe illness (whether febrile or not)
- The volunteer has received a live vaccine within the preceding 28 days
- The investigator has any other concern that challenge may not be in the volunteer's best interests
- The volunteer has a recent positive SARS-CoV-2 PCR result or symptoms highly consistent with COVID-19 disease since screening (unless had a negative PCR result at that time and symptoms have resolved).

In these cases, the volunteer may be challenged at a later date or withdrawn from the study at the discretion of the investigator.

Bronchoscopy Postponement criteria

Bronchoscopy will not proceed on the scheduled day in any of the following situations:

- The volunteer has a temperature $\geq$ 37.8°C
- The investigator judges the volunteer to have an acute moderate or severe illness (whether febrile or not)
- The investigator has any other concern that bronchoscopy may not be in the volunteer's best interests
- The volunteer has a positive SARS-CoV-2 PCR result or symptoms which the investigator considers to be highly consistent with COVID-19 disease since enrolment (unless had a negative PCR result at that time and symptoms have resolved).

In these cases, the volunteer may proceed to bronchoscopy at a later date (ideally next available), not undergo a bronchoscopy but continue with other follow up visits or be withdrawn from the study at the discretion of the investigator.

7.3.5 Prevention of 'Over Volunteering'

Volunteers will be excluded from the study if they are concurrently involved in another trial where an IMP has been administered within 30 days prior to enrolment, or will be administered during the study period. In order to ensure this, volunteers will be asked to provide their National Insurance or Passport number (if they are not entitled to a NI number) and will be registered on a national database of participants in clinical trials (www.tops.org.uk). They will not be enrolled if found to be actively registered on another trial.

7.3.6 Withdrawal of Volunteers

Every reasonable effort will be made to maintain protocol compliance and participation in the study.

In accordance with the principles of the current revision of the Declaration of Helsinki and any other applicable regulations, a volunteer has the right to withdraw from the study at any time and for any reason, and is not obliged to give his or her reasons for doing so. The investigator may withdraw the volunteer at any time in the interests of the volunteer's health and well-being (including on the advice of the SMC). In addition, the volunteer may withdraw/be withdrawn for any of the following reasons:

- Administrative decision by the investigator
- Ineligibility (either arising during the study or retrospectively, having been overlooked at screening)
- Significant protocol deviation
- Volunteer non-compliance with study requirements
- An AE, which requires discontinuation of the study involvement or results in inability to continue to comply with study procedures
- Confirmed pregnancy during the study

The reason for withdrawal will be recorded in the Care Report Form (CRF). If withdrawal is due to an AE, appropriate follow-up visits or medical care will be arranged, with the agreement of the volunteer, until the AE has resolved, stabilised or a non-study related causality has been assigned. Any volunteer who is withdrawn from the study may be replaced at the decision of the investigator. The Safety Monitoring Committee (SMC) may recommend withdrawal of volunteers. Any volunteer who fails to attend for two or more follow-up visits during the study, despite active attempts to contact volunteer by study team, will be deemed to have withdrawn from the study.

If a volunteer withdraws from the study, samples and data collected before their withdrawal from the study will be used/stored unless the volunteer specifically requests otherwise. Long-term safety data collection will continue as appropriate if a volunteer has received a challenge dose.

7.4 Compliance with Dosing Regime

The volunteers will receive one dose of BCG only at enrolment.

7.5 Pregnancy

Should a volunteer become pregnant during the study, she will be followed up for clinical safety assessment with her ongoing consent and in addition will be followed until pregnancy outcome is determined. Any baby born may need to be followed up. We would not routinely perform venepuncture on a pregnant volunteer unless there is clinical need.

8. CLINICAL PROCEDURES

This section describes the clinical procedures for evaluating study participants and follow-up after administration of the study challenge.

8.1 Schedule of Attendance

All volunteers will have the same schedule of clinic attendances and procedures as indicated in Table 4. The total volume of blood donated during the study will be around 523ml. Additional visits or procedures may be performed at the discretion of the investigators, e.g. further medical history and physical examination, blood tests in event of blood AE, COVID-19 testing in the event of suggestive symptoms, or urine microscopy in the event of positive urinalysis.

8.2 Observations

Pulse, blood pressure, oxygen saturations and temperature will be measured at the time-points indicated in the schedule of procedures and may also be measured as part of a physical examination if indicated at other time-points.

8.3 Blood tests, urinalysis, bronchial samples

Urinalysis
Near-patient testing on urine will be performed at the CCVTM. Urine will be tested for protein, blood and glucose at screening. For female volunteers only, urine will be tested for beta-human chorionic gonadotrophin (β-HCG) at screening and prior to BCG challenge and bronchoscopy

Blood will be drawn for the following laboratory tests and processed at contractually agreed National Health Service (NHS) Trust laboratories using NHS standard procedures:

- Haematology; Full Blood Count
- Biochemistry; Sodium, Potassium, Urea, Creatinine, Albumin, Liver Function Tests (Bilirubin, ALT, ALP)
- Coagulation screen: Prothrombin time, APTT
- Diagnostic serology; HBsAg, HCV antibodies, HIV antibodies (specific consent will be gained prior to testing blood for these blood-borne viruses)
- Immunology; Human Leukocyte Antigen (HLA) typing; TB IGRA

Blood and bronchial samples will be processed at University of Oxford research laboratories:

- Exploratory Immunology: Immunogenicity will be assessed by a variety of immunological assays. This includes T and B cell assays which may include ex-vivo ELISpot assays for interferon gamma and flow cytometry, analysis of antibody responses such as ELISA. Other exploratory immunological assays including cytokine analysis, DNA analysis of genetic polymorphisms potentially relevant to vaccine immunogenicity and gene expression studies (both host and parasite) amongst others which may be performed at the discretion of the investigators. Mycobacterial GIA will be used to identify potential immune correlates of protection.
- Quantification of BCG recovered from bronchial samples will be undertaken using methods which may include quantitative PCR, culture including mycobacterial growth indicator tube (MGIT) and/or mycobacterial speciation including molecular genetic assays.

All initial investigations will be outlined in the study-specific laboratory plan and conducted according to local SOPs. Exploratory immunology assays may be performed under the OVC Biobank ethical approval.

Collaboration with other specialist laboratories in the UK, Europe and outside of Europe for further exploratory tests may occur. This would involve the transfer of serum, plasma, PBMC, BALF, mask PVA matrix strips and/or other study samples to these laboratories, but these would remain anonymised. Informed consent for this will be gained from volunteers.

Participants will be informed that there may be leftover samples (after all testing for this study is completed), and that such samples may be stored for possible future research (exploratory immunology), including genotypic testing of genetic polymorphisms potentially relevant to vaccine immunogenicity. Participants will be able to decide if they will permit such future use of any leftover samples. With the volunteers' informed consent, any leftover cells, serum/plasma, BALF or any other leftover samples will be frozen indefinitelyas per the Oxford Vaccine Centre Biobank consent for future analysis of tuberculosis-specific or vaccine-related responses. If a participant elects not to permit this, all of that participant's leftover samples will be discarded after the required period of storage to meet Good Clinical Practice (GCP) and regulatory requirements.

Samples that are to be stored for future research will be transferred to the OVC Biobank (REC 16/SC/0141).

8.4 SARS-COV-2 Testing

During the COVID 19 pandemic, participants will be tested for SARS-COV-2 infection prior to challenge, bronchoscopy and induced sputum procedures as per the schedule of attendances (table 4). PCR Tests will be processed at contractually agreed NHS Trust laboratories using NHS standard procedures. Results require notification to the relevant public health authority, which will be performed by the NHS trust laboratory processing the test. Samples will therefore be sent with the participant's personal identifiers including name and NHS number.

8.5 Mask wearing

Participants will be asked to wear an adaptedduckbill facemask for 30 minutes prior to and following D0 challenge, for 30 minutes immediately prior to bronchoscopy, and for 30 minutes at the Day 2, Day 7, Day 28 and 6 month follow-up visits. Each duckbill face mask (Integrity® 600-300) contains a collection matrix which consists of strips of Polyvinyl Alcohol (PVA) produced by 3D printing. This system allows the capture of aerosolized droplets from the lungs. These masks, which are categorised as "sample containers" under the MHRA in vitro diagnostics regulation, will be manufactured and supplied by Professor Michael Barer at the University of Leicester. Masks will be appropriately labelled to allow batch identification. Collection matrix samples at each time point will be divided for processing:

- At the University of Oxford Research Laboratories for evaluation of BCG recovery using MGIT culture. Analysing the matrix will allow us to compare bacterial recovery at baseline and over time
- With our collaborators at the University of Leicester for further exploratory and molecular assays

All investigations using the PVA matrix will be conducted according to site specific SOPs and mask batch number will be recorded on laboratory processing records. Evaluation and use of this technology in the TB044 study will be registered with the MHRA portal by the University of Leicester as manufacturers of the device. No additional MHRA approval is required.

8.6 Diary Card

Following the BCG challenge, volunteers will be asked to complete a diary card, which may be in paper or electronic format. Volunteers will be asked to record any specific solicited and unsolicited AEs daily, for 28 days. Diary cards will be reviewed with volunteers at the post-challenge follow up visits.

8.7 Study visits

The study visits and procedures will be undertaken by one of the clinical trials team. The procedures to be included in each visit are documented in the schedule of attendances. Each visit is assigned a time-point and a window period, within which the visit will be conducted.

8.7.1 Screening visit

All potential volunteers will have a screening visit, which may take place up to 120 days prior to vaccination. Informed consent will be taken before screening, as described in section 7.2. If consent is obtained, the procedures indicated in the schedule of attendances will be undertaken including a medical history, physical examination and blood tests. To avoid unnecessary additional venepuncture, if the appropriate blood test results for screening are available for the same volunteer from a screening visit for another Jenner Institute Clinical Trials group vaccine study, these results may be used for assessing eligibility (provided the results date is within the 3 months preceding enrolment in TB044).

Information will be recorded related to prior BCG vaccination, including date and country/region of vaccination (if known) and whether the volunteer has received more than one BCG vaccination. This will be recorded in the screening CRF. BCG World Atlas (www.bcgatlas.org), a database of global BCG policies and practices, may be consulted to gain further information including likely BCG strain and administration device if available.

The participant's general practitioner will be contacted with the written permission of the participant after screening to ascertain any significant medical history and as notification that the participant has volunteered for the study. During the screening, the volunteers will be asked to provide their national insurance or passport number so that this can be entered on to a national database which helps prevent volunteers from participating in more than one clinical trial simultaneously or over-volunteering for clinical trials (www.tops.org.uk). Abnormal clinical findings from the urinalysis or blood tests at screening will be assessed as detailed in VC027: Adverse Event Data Collection and Analysis.

Abnormal blood tests following screening will be assessed according to site-specific laboratory adverse event grading tables which are listed in the SOP. Any abnormal test result deemed clinically significant may be repeated to ensure it is not a single occurrence. If an abnormal finding is deemed to be clinically significant, the volunteer will be informed and referral for appropriate medical care arranged with the permission of the volunteer.

The eligibility of the volunteer will be reviewed at the end of the screening visit and again when all results from the screening visit have been considered. Decisions to exclude the volunteer from enrolling in the study or to withdraw a volunteer from the study will be at the discretion of the investigator. If eligible, a day 0 visit will be scheduled for the volunteer to receive the challenge and subsequent follow-up.

8.7.2 D0-2

Volunteers will be tested for SARS-CoV-2 during the pandemic period.

8.7.3 Day 0: Enrolment and vaccination visit

Volunteers will be considered enrolled in to the study at the point of BCG challenge. Enrolment should take place no longer than 120 days following the date of screening. If more than 120 days elapse, focussed rescreening of the volunteer should take place prior to enrolment to elicit any changes in medical circumstances and to repeat necessary blood tests.

Before BCG challenge, the eligibility of the volunteer will be reviewed. Pulse, blood pressure, oxygen saturations and temperature will be observed and lung function measured. If necessary, a medical history and physical examination may be undertaken to determine need to postpone BCG challenge depending on criteria listed in section 7.3.4. During the pre-challenge checks the volunteer will be asked to wear the duckbill mask for 30 minutes. BCG challenge will be administered as described below.

8.7.3.1 D0 Challenge

All movements of vials of the study agent in or out of the locked refrigerator will be documented. BCG accountability, storage, shipment and handling will be in accordance with local SOPs and other relevant local forms.

BCG will be administered by aerosol inhalation, according to the study-specific SOP. Volunteers will stay in the unit for 60 minutes (±10 minutes) after challenge. During the administration of BCG, monitoring equipment, oxygen, medicines including bronchodilators and resuscitation equipment will be immediately available for the management of anaphylaxis and bronchospasm according to the study-specific SOP.

In order to minimise dissemination of the BCG bacteria into the environment and to ensure the protection of staff, measures will be instituted during and following challenge to fully comply with local infection control and occupational health regulations.

Following challenge the following procedures will be undertaken:
The volunteers will be asked to wear a duckbill mask for another 30 minute period. Volunteers will be given an oral thermometer and a diary card to complete for 28 days with instructions on use, along with the emergency 24 hour telephone number to contact the on-call study physician if needed. Volunteers will be instructed on how to self-assess the severity of AEs. Specific solicited local and systemic AEs will be asked about. There will also be space in the diary card to self-document unsolicited AEs, and whether medication was taken to relieve any symptoms. If bronchoscopy is postponed, a further diary card may be utilised to ensure AEs for 7 days post bronchoscopy are captured.

Diary cards will collect information on the timing and severity of the following solicited AEs:

**Local (respiratory) solicited AEs**	**Systemic solicited AEs**
Cough (any cough)	Documented Fever (oral temp >37.5°C)
Coughing up phlegm	Feverishness
Coughing up blood	Joint pains (arthralgia)
Wheeze	Muscle pains (myalgia)
Shortness of breath	Fatigue
Sore throat	Headache
Tickly throat	Malaise
Chest tightness	Nausea
Chest pain	

Table 3. Solicited AEs as collected in post challenge diary cards.

8.7.3.2 Sequence of Enrolment and challenge of volunteers

Volunteers will be sequentially allocated into each group according to availability.

8.7.4 Subsequent visits: Day 2, 7, 14, 28, 56, 84 and 168

Follow-up visits will take place as documented in the visit schedule (Table 4). Volunteers will be assessed for local and systemic adverse events and interim history. Lung function, mask wearing and blood tests will take place at these time points as detailed in the schedule of attendances. Blood will also be taken for exploratory immunology. Bronchoscopy will be undertaken at the day 14 follow up visit. Induced sputum procedure may take place at the Day 28 and 6 month visits depending on resources and current COVID-19 pandemic situation. Lateral flow testing may be performed.

If volunteers experience adverse events (laboratory or clinical), which the investigator (physician), CI and/or SMC determine necessary for further close observation, the volunteer may be admitted to the infectious diseases or respiratory ward at Oxford University Hospitals, Oxford for observation and further medical management under the care of the consultant on call.

8.7.5 Bronchoscopy visit – 2 days

Volunteers will be tested for SARS-CoV-2 during pandemic period.

Table 4: Schedule of visits

**Visit number**	**1**	**2**	**3**	**4**	**5**	**6**	**7**	**8**	**9**	**10**	**11**	**12**	**13**
Timeline (days)*	Screen	-2	0	2	7	B-2	14	IS-2	28	56	84	IS-2	168
Time windows (days)		±2		±1	±2	±2	-5 to +21	±2	-7 to +14	±14	±21	±2	±28
Inclusion/exclusion criteria	X												
Review contra-indications	X		X				X						
Informed consent	X						X						
Medical history	X		(X)	(X)	(X)		(X)		(X)	(X)	(X)		(X)
Physical examination	X		(X)	(X)	(X)		(X)		(X)	(X)	(X)		(X)
Vital signs	X		X	X	X		X		X	X	X		X
Urinalysis	X												
PFTs	X		X	X	X		(X)		(X)	(X)	(X)		(X)
β-HCG urine test (females only)	X		X				X						
SARS-CoV-2 test**		X				X		(X)$^{+}$				(X)$^{+}$	
**BCG Challenge**			X										
**Bronchoscopy**							X						
Chest radiograph	X												
Mask wearing			X	X	X		X		X				X
Induced Sputum Procedure^									(X)				(X)
Local & systemic events			X	X	X		X		X	X	X		X
Diary card setup			X				(X)						
Diary card final review									X				
Biochemistry (U+Es, LFTs)	3		3		3		3		3				
Haematology (FBC)	2		2		2		2		2				
Coagulation (PT, APTT)	3												
HBV, HCV, HIV	5												
TB IGRA	6												
HLA typing			4										
Exploratory immunology			60	60	60		60		60	60	60		60
Blood vol per visit (mL)	19		69	60	65		65		65	60	60		60
Cumulative blood vol (mL)$^{\%}$	19		88	148	213		278		343	403	463		523

* Timeline is approximate, exact visit timings (± windows periods) relate to actual (not intended) date of previous visit
X Event scheduled to occur B-2 = bronchoscopy -2 days IS-2 = Induced Sputum -2 days
(X) If considered necessary, emphasising any complaint or change in medication
** During COVID-19 pandemic (X) If considered necessary based on current COVID-19 regulations.
$^{+}$May be lateral flow test or PCR
^ may be undertaken depending on resources and current COVID-19 situation. SARS-CoV-2 testing may be undertaken prior to induced sputum.

9. STUDY AGENTS AND DEVICES

9.1 BCG Description

9.1.1 BCG Danish 1331

BCG Danish 1331 is a dried preparation containing live bacteria derived from an attenuated strain of *Mycobacterium bovis* BCG. It is supplied as a powder and diluent for suspension. Each vial contains 2-8x10^6 cfu BCG (see SmPC).

9.1.2 Storage of BCG

BCG Danish will be shipped to the CCVTM, University of Oxford, Churchill Hospital, following standard vaccine cold chain precautions.

Dispensed BCG vials are supplied in boxes containing multiple vials and each box is clearly labelled with the market product and identified for local use.
BCG will be stored at +2 to +8°C (nominal temperature) in a secure, temperature-monitored refrigerator at the CCVTM, University of Oxford, Churchill Hospital.

9.2 Dispensing and administration

All movements of vials of the study agent in or out of the locked refrigerator will be documented. BCG accountability, storage, shipment and handling will be in accordance with local SOPs and other relevant local forms.

BCG will be administered by aerosol inhalation, according to the study-specific SOP. Volunteers will stay in the unit for 60 minutes (±10 minutes) after challenge. During the administration of BCG, monitoring equipment, oxygen, medicines including bronchodilators and resuscitation equipment will be immediately available for the management of anaphylaxis and bronchospasm according to the study-specific SOP.

In order to minimise dissemination of the BCG bacteria into the environment and to ensure the protection of staff, measures will be instituted during and following challenge to fully comply with local infection control and Occupational Health regulations.

9.3 Saline

0.9% saline will be mixed with the reconstituted BCG to add to the nebuliser.

Hypertonic saline (3%) will be delivered via nebuliser during the induced sputum procedure according to the study-specific SOP.

9.4 Salbutamol

Volunteers undergoing the induced sputum procedure will be pre-treated with a prescribed dose of salbutamol (200mcg delivered via spacer) in accordance with the European Respiratory Society's guidelines [48].

9.5 MicroAIR NE-U22/U100

This nebuliser is used for aerosol delivery of the BCG. This is an approved electromedical device, CE0197, EAN code 40 15672 10142. Information about the specifications, usage, and maintenance of the nebuliser device can be found in the device documentation (39).

9.6 Ultrasonic nebuliser NE-U780

This nebuliser is used for delivering the hypertonic saline during the induced sputum procedure. This is an approved electromedical device, CE0197, EN60601-1-2-2007. Information about the specifications, usage, and maintenance of the nebuliser device can be found in the device documentation [49].

9.7 Sedative & anaesthetic agents for bronchoscopy

Fentanyl is a licensed opioid used routinely to provide analgesia and sedation during medical procedures. It will be stored, dispensed and administered in accordance with standard NHS procedures and the SmPC (40). Midazolam is a licensed benzodiazepine used routinely to provide sedation and amnesia during medical procedures. It will be stored, dispensed and administered in accordance with standard NHS procedures and the SmPC (41). Lignocaine is a licensed local anaesthetic used routinely during medical procedures. It will be stored, dispensed and administered in accordance with standard NHS procedures and the SmPC (42). Other licensed drugs may be used during bronchoscopy at the discretion of the respiratory consultant performing the bronchoscopy and/or the investigators.

10. ASSESSMENT OF SAFETY

Safety will be assessed by the frequency, incidence and nature of AEs and SAEs arising during the study.

10.1 Definitions

10.1.1 Adverse Event (AE)

An AE is any untoward medical occurrence in a volunteer, which may occur during or after administration of the BCG and does not necessarily have a causal relationship with the intervention. An AE can therefore be any unfavourable and unintended sign (including any clinically significant abnormal laboratory finding or change from baseline), symptom or disease temporally associated with the study intervention, whether or not considered related to the study intervention.

10.1.2 Adverse Reaction (AR)

An AR is any untoward or unintended response to the challenge agent (BCG). This means that a causal relationship between the agent and an AE is at least a reasonable possibility, i.e., the relationship cannot be ruled out. All cases judged by the reporting medical investigator as having a reasonable suspected causal relationship to the agent (i.e. possibly, probably or definitely related to it) will qualify as AR.

10.1.3 Unexpected Adverse Reaction

An adverse reaction, the nature or severity of which is not consistent with the applicable product information (e.g. SmPC).

10.1.4 Serious Adverse Event (SAE)

An SAE is an AE that results in any of the following outcomes, whether or not considered related to the study intervention.

- Death
- Life-threatening event (i.e. the volunteer was, in the view of the investigator, at immediate risk of death from the event that occurred). This does not include an AE that, if it occurred in a more severe form, might have caused death.
- Persistent or significant disability or incapacity (i.e. substantial disruption of one's ability to carry out normal life functions).
- Hospitalisation, regardless of length of stay, even if it is a precautionary measure for continued observation. Hospitalisation (including inpatient or outpatient hospitalisation for an elective procedure) for a pre-existing condition that has not worsened unexpectedly does not constitute a serious AE.
- An important medical event (that may not cause death, be life threatening, or require hospitalisation) that may, based upon appropriate medical judgment, jeopardise the volunteer and/or require medical or surgical intervention to prevent one of the outcomes listed above. Examples of such medical events include allergic reaction requiring intensive treatment in an emergency room or clinic, blood dyscrasias, or convulsions that do not result in inpatient hospitalisation.
- Congenital anomaly or birth defect.

10.1.5 Serious Adverse Reaction (SAR)

An AE (expected or unexpected) that is both serious and, in the opinion of the reporting investigator or sponsors, believed to be possibly, probably or definitely due to the challenge agent or any other study treatments, based on the information provided.

10.1.6 Suspected Unexpected Serious Adverse Reaction (SUSAR)

A serious adverse reaction, the nature and severity of which is not consistent with the information about the medicinal product in question set out in the SmPC.

10.2 Expectedness

No BCG related SAEs are expected in this study. All SARs will therefore be reported as SUSARs.

10.3 Causality

For every AE, an assessment of the relationship of the event to the administration of BCG will be undertaken by the CI or delegated clinician. An interpretation of the causal relationship of the intervention to the AE in question will be made, based on the type of event; the relationship of the event to the time of BCG challenge; and the known biology of BCG (Table 5). Alternative causes of the AE, such as the natural history of pre-existing medical conditions, concomitant therapy, other risk factors and the temporal relationship of the event to BCG administration will be considered and investigated. Causality assessment will take place during planned safety reviews, interim analyses (e.g. if a holding or stopping rule is activated) and at the final safety analysis, except for SAEs, which should be assigned by the reporting investigator.

0	**No Relationship**	No temporal relationship to study product ***and***<br>Alternate aetiology (clinical state, environmental or other interventions); ***and***<br>Does not follow known pattern of response to study product
1	**Unlikely**	Unlikely temporal relationship to study product ***and***<br>Alternate aetiology likely (clinical state, environmental or other interventions) ***and*** does not follow known typical or plausible pattern of response to study product.
2	**Possible**	Reasonable temporal relationship to study product; ***or***<br>Event not readily produced by clinical state, environmental or other interventions; ***or***<br>Similar pattern of response to that seen with other challenge agents
3	**Probable**	Reasonable temporal relationship to study product; ***and***<br>Event not readily produced by clinical state, environment, or other interventions ***or***<br>Known pattern of response seen with other challenge agents
4	**Definite**	Reasonable temporal relationship to study product; ***and***<br>Event not readily produced by clinical state, environment, or other interventions; ***and***<br>Known pattern of response seen with BCG

Table 5. *Guidelines for assessing the relationship of challenge administration to an AE.*

10.4 Reporting Procedures for All Adverse Events (see local SOP for AE reporting)

All AEs occurring in the 14 days following challenge and the 14 days following bronchoscopy observed by the investigator or reported by the volunteer, whether or not attributed to BCG, will be recorded on the diary card. Data from the diary cards will be extracted following the last volunteer last visit (LVLV) or at any time prior to this in order to perform an interim safety analysis. Outside the diary periods, respiratory and systemic AEs (listed in 6) will be specifically solicited at each visit, and graded by severity (as detailed in section 9.5). All AEs starting after the diary period(s), or persisting after this period, will be recorded in the AE line listing of the CRF.

Recording and reporting of all AEs will take place as detailed in SOP VC027 Adverse Event Data Collection and Analysis. All AEs that result in a volunteer's withdrawal from the study will be followed up until a satisfactory resolution occurs, or until a non-study related causality is assigned (if the volunteer consents to this). SAE will be collected throughout the entire study period.

	**Adverse event**
**Respiratory**	Cough
	Sore throat
	Tickly throat
	Wheeze
	Shortness of breath
	Coughing up phlegm
	Coughing up blood
	Chest tightness
	Chest pain
**Systemic**	Documented fever (oral temperature > 37.5° C)
	Myalgia
	Arthralgia
	Feverishness
	Headache
	Fatigue
	Nausea
	Malaise

Table 6. Routinely solicited adverse events

10.5 Assessment of severity

The severity of clinical and laboratory adverse events will be assessed according to the scales in table 7 and 8.

	**Grade 1 (mild)**	**Grade 2 (moderate)**	**Grade 3 (severe)**
Fever (oral)	37.6°C - 38.0°C	38.1°C – 39.0°C	>39.0°C
Tachycardia (bpm)*	101 - 115	116 – 130	>130
Bradycardia (bpm)**	50 – 54	40 – 49	<40
Systolic hypertension (mmHg)	141 - 159	160 – 179	≥180
Diastolic hypertension (mmHg)	91 – 99	100 – 109	≥110
Systolic hypotension (mmHg)***	85 – 89	80 – 84	<80

Table 7. ***Severity grading criteria for physical observations.*** **Taken after ≥10 minutes at rest **When resting heart rate is between 60 – 100 beats per minute. Use clinical judgement when characterising bradycardia among some healthy participant populations, for example, conditioned athletes. ***Only if symptomatic (e.g. dizzy/ light-headed)*

**GRADE 0**	None
**GRADE 1**	Mild: Transient or mild discomfort (< 48 hours); no medical intervention/therapy required
**GRADE 2**	Moderate: Mild to moderate limitation in activity – some assistance may be needed; no or minimal medical intervention/therapy required
**GRADE 3**	Severe: Marked limitation in activity, some assistance usually required; medical intervention/therapy required, hospitalisation possible

Table 8. Severity grading criteria for local and systemic AEs.

10.6 Reporting Procedures for Serious AEs

All SAEs will be reported on the SAE forms to members of the study team immediately, once the Investigators become aware of their occurrence. Copies of all reports will be forwarded for review to the Chief Investigator (as the Sponsor's representative) within 24 hours of the Investigator being aware of the suspected SAE. The safety monitoring committee (SMC) chair will be notified of SAEs that are deemed possibly, probably or definitely related to study interventions; the SMC will be notified immediately (within 24 hours) of the Investigators' being aware of their occurrence. The funder will be informed of all SAE notifications reported to the SMC. SAEs will not normally be reported immediately to the ethical committee(s) unless there is a clinically important increase in occurrence rate, an unexpected outcome, or a new event that is likely to affect safety of study volunteers, at the discretion of the Chief Investigator and/or SMC.

A serious adverse event (SAE) occurring to a participant should be reported to the REC that gave a favourable opinion of the study where in the opinion of the Chief Investigator the event was ‘related’ (resulted from administration of any of the research procedures) and ‘unexpected’ in relation to those procedures. Reports of related and unexpected SAEs should be submitted within 15 working days of the Chief Investigator becoming aware of the event.

10.7 Procedures to be followed in the event of abnormal findings

Eligibility for enrolment in the study in terms of laboratory findings will be assessed as detailed in SOP VC027. Abnormal clinical findings from medical history, examination or blood tests will be assessed as to their clinical significance throughout the study. Laboratory AEs will be assessed using the site-specific tables in SOP VC027. If a test is deemed clinically significant, it may be repeated, to ensure it is not a single occurrence. If a test remains clinically significant, the volunteer will be informed and appropriate medical care arranged as appropriate and with the permission of the volunteer. Decisions to exclude the volunteer from enrolling in the study or to withdraw a volunteer from the study will be at the discretion of the Investigator.

10.8 Interim Safety Reviews

The safety profile of aerosol inhaled BCG will be assessed on an on-going basis by the investigators with communication to the SMC as necessary. The CI and relevant investigators (as per the study delegation log) will also review safety issues and SAEs as they arise.

The SMC will be consulted to provide a review of safety data and adverse events at least 7 days following completed enrolment of each group and prior to proceeding to dose escalation. All reports to the SMC will be shared with the funder.

10.9 Safety Monitor Committee

An independent Safety Monitoring Committee (SMC) will be appointed to provide real-time safety oversight. The SMC will be notified within 24 hours of the investigators’ being aware of the occurrence of SAEs. The SMC has the power to place the study on hold if deemed necessary following a study intervention-related SAE. The SMC will be chaired by Dr Francesca Little with Dr Hassan Mahomed and Professor Stephen Gordon as committee members. All correspondence between the investigators and SMC will be conveyed by the investigator to the study sponsor or their delegate. The Chair of the SMC will be contacted for advice and independent review by the investigator or study sponsor in the following situations:

- Following any SAE deemed to be possibly, probably, or definitely related to BCG.
- Prior to dose escalation occurring
- Any other situation where the investigator or study sponsor feels independent advice or review is important

10.10 Safety Group Holding Rules

Safety holding rules have been developed to take into account the fact that this is a dose escalation study of aerosolised BCG in BCG historically-vaccinated volunteers. Solicited AEs are those listed as foreseeable AEs in section 10.4 of the protocol. Unsolicited adverse events are adverse events other than these foreseeable AEs.

10.10.1 Group holding rules

The following would act as a trigger to place the study on hold and the SMC would need to be consulted prior to recommencement:

- Solicited local (respiratory) adverse events: If more than one dose of the challenge agent is followed by the same grade 3 solicited local AE beginning within 2 days after challenge (day of challenge and one subsequent day) and persisting at Grade 3 for >48 hours.
- Solicited systemic adverse events: If more than one dose of the challenge agent is followed by the same grade 3 solicited systemic AE beginning within 2 days after challenge (day of challenge and one subsequent day) and persisting at grade 3 for >48 hours.
- Unsolicited adverse events: If more than one volunteer develops a grade 3 unsolicited AE (that is considered possibly, probably or definitely related to challenge) and persists at grade 3 for >48 hours.
- Laboratory adverse event: If more than one volunteer develops the same grade 3 laboratory AE considered possibly, probably or definitely related that persists at grade 3 for >72 hours
- Any serious adverse event considered possibly, probably or definitely related to the challenge agent occurs
- Death considered possibly, probably or definitely related to challenge occurs
- A life-threatening reaction considered possibly, probably or definitely related to challenge occurs
  Communication of any study pauses will be shared with the funder.

Previously, we had planned to wait 14 days after challenge prior to safety review and further challenge or dose escalation, based on timing of AEs collected in TB041 (NCT02709278). Mild local (respiratory) solicited AEs were seen to continue for at least 2 weeks in several volunteers (volunteers underwent bronchoscopy at 2 weeks, so local AEs seen after this would be expected due to this procedure). A possible small increase in systemic solicited AEs was seen at around day 13. Several unsolicited AEs of grade 1 or 2 “chest tightness” were seen around the day 8-12 mark and the 14 day review period was planned in TB044 in case there was a clustering of chest symptoms around the Day 10 mark.

Chest tightness was added as a solicited symptoms in TB043. As groups 1 and 2 in TB043 underwent a bronchoscopy prior to day 14, this significantly confounded the adverse event profile in the first two weeks. Now we have further data from groups 3-5 in TB043, who do not undergo bronchoscopy until after the initial 14 days solicited symptoms diary is complete. This data has shown no evidence of late clustering of chest symptoms or other adverse events. In all of these TB043 volunteers who have received 1 x 10^7 BCG (n=20), there has been no chest tightness reported after Day 7. There have been no reported respiratory AEs above a Grade 1 after Day 6 (Figure 5). There has been no evidence of a clustering of respiratory symptoms around the Day 10 timepoint, and therefore we plan to shorten our safety review duration to 7 days, in keeping with more normal practice in dose escalation or first in human studies.

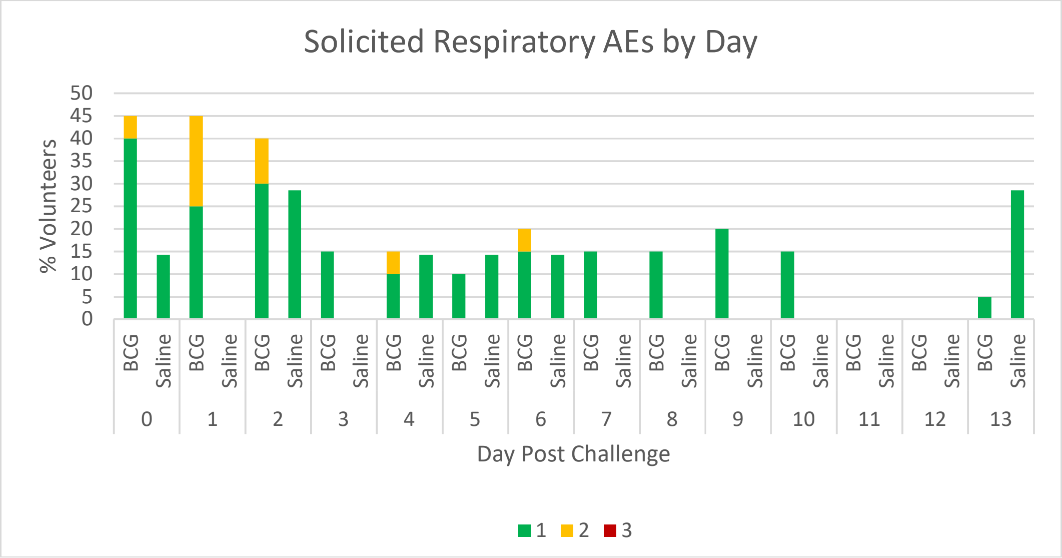

Figure 5. TB043. Solicited AE within 2 weeks of aerosol BCG (1 x 10^7) or saline placebo

If a holding rule has been met and, following an internal safety review by the SMC it is deemed appropriate to restart dosing, a request to restart dosing with pertinent data will be submitted to the REC. The internal safety review will consider:

- The relationship of the AE or SAE to the challenge agent.
- The relationship of the AE or SAE to the challenge dose, or other possible causes of the event.
- If appropriate an amendment will be submitted, for example with additional screening or laboratory testing for other volunteers to identify those who may develop similar symptoms and alterations to the current Participant Information Sheet (PIS) if required.

The local ethics committee will be notified if a holding rule is activated or released.

All enrolled volunteers will be followed for safety until resolution or stabilisation (if determined to be chronic sequelae) of their AEs, providing they consent to this.

10.10.2 Individual stopping rules (will apply to all vaccinated individuals)

Separate individual stopping rules will not apply in this study, as each volunteer will only receive one challenge dose of BCG. Postponement criteria are in place for bronchoscopy, see section 7.3.4.

In addition to these pre-defined criteria, the study can be put on hold upon advice of the Chief Investigator, Study Sponsor, regulatory authority, Ethical Committee(s) or Safety Monitoring Committee, for any single event or combination of multiple events which, in their professional opinion, jeopardise the safety of the volunteers or the reliability of the data.

11. STATISTICS

This is primarily an exploratory study with descriptive endpoints. We will recruit a total of 12 participant into the study. Our previous experience with similar clinical studies suggests that this sample size is a feasible number to recruit, screen, enrol, and follow up in practical terms, whilst also allowing the evaluation of the human response to BGC challenge and immunogenicity of aerosol inhaled BCG in this population. The sample size has not been determined with the aim of achieving statistical significance.

Based on previous experience with similar studies, it is expected that the amount of missing, unused or spurious data will be insignificant. Unused and spurious data will be listed separately and excluded from the statistical analysis. Missing data will be excluded from the statistical analysis.

A detailed statistical analysis plan will be prepared prior to the data lock at the end of the study. Any deviations from this plan will be detailed in the analysis report. Statistical support will be provided by an ongoing collaboration with the statistical team at the Nuffield Department of Primary Care Health Sciences Clinical trials Unit (safety and non-transcriptomic data) and with the Jenner Transcriptomic Core Facility (transcriptomic data).

12. DATA MANAGEMENT

12.1 Data Handling

The Chief Investigator will be responsible for all data that accrues from the study.

The data will be captured directly into the volunteers' electronic CRFs or transferred from a paper source in to the eCRF created on the study specific database designed using OpenClinica ™ (an open source clinical trial software for Electronic Data Capture (EDC)). Electronic data will be stored on secure servers, which are outsourced by OpenClinica™. Data will be entered directly into the study database via a web browser using encrypted (https) transfer. OpenClinica™ meets FDA part 11B standards. This includes safety data, laboratory data and outcome data.

All adverse event data (both solicited and unsolicited) reported by the volunteer will be entered onto a volunteer's electronic diary card for a maximum of 14 days following the administration of BCG and 14 days following the bronchoscopy. The Diary provides a full audit trial of edits and will be reviewed at each review time-points indicated in the schedule of events. Any adverse event continuing beyond the period of the diary will be copied into the eCRF (OpenClinica) and followed to resolution, if there is a causal relationship to BCG, or to the end of the study if there is no causal relationship.

The participants will be identified by a unique study specific number and code in any database. The name and any other identifying detail will NOT be included in any study data electronic file.

12.2 Record Keeping

The Investigators will maintain appropriate medical and research records for this study, in compliance with GCP and regulatory and institutional requirements for the protection of confidentiality of volunteers. The Chief Investigator, co-Investigators and clinical research nurses will have access to records. The Investigators will permit authorised representatives of the Sponsor(s), as well as ethical and regulatory agencies to examine (and when required by applicable law, to copy) clinical records for the purposes of quality assurance reviews, audits and evaluation of the study safety and progress.

With the volunteers' consent, we will keep their contact details after participation in the study is complete, so we may inform them of opportunities to participate in future vaccine related research. This will be entirely optional and participation in this study will not be affected by their decision to allow or not allow storage of their contact details beyond participation in this study. Details will be stored electronically on a secure server and only authorised individuals at the CCVTM will have access to it. We will not, under any circumstances, share their contact details with any third party institutions without their permission. Volunteers will be informed that being contacted does not oblige them to agree to take part in future research and they can ask us to have their contact details removed from our database at any time

All original source records will be kept on site for the duration of the study. Following the closure of the study, all records will be transfer to a secure archiving facility for a maximum of 7 years. Subsequently a data base will be maintained with de-identified data.

12.3 Source Data and Case Report Forms (CRFs)

All protocol-required information will be collected in CRFs designed by the investigator. All source documents will be filed in the CRF. Source documents are original documents, data, and records from which the volunteer's CRF data are obtained. For this study, these will include, but are not limited to, volunteer consent form, blood and microbiology results, radiology report, GP response letters, copy of bronchoscopy report, laboratory records, diaries and correspondence. In the majority of cases, CRF entries will be considered source data as the CRF is the site of the original recording (i.e. there is no other written or electronic record of data). In this study this will include, but is not limited to medical history, medication records, vital signs, physical examination records, urine assessments, blood results, bronchial sample results, adverse event data and details of BCG administration. All source data and volunteer CRFs will be stored securely.

On all study-specific documents, other than the contact form, signed consent, the chest X-ray report, GP/medical correspondence, formal pulmonary function testing and the bronchoscopy report, the participant will be referred to by the study participant number/code, not by name.

12.4 Data Protection

The study will comply with the UK General Data Protection Regulation (GDPR) and Data Protection Act 2018, which require data to be de-identified as soon as it is practical to do so. The processing of the personal data of participants will be minimised by making use of a unique participant study number only on all study documents and any electronic database(s). All documents will be stored securely and only accessible by study staff and authorised personnel. The study staff will safeguard the privacy of participants' personal data. Anonymised safety data will be shared with the funder on a regular basis. In the event of any SAEs, pseudo-anonymised data will be shared with the funder. No information concerning the study or the data will be released to any unauthorised third party, without prior written approval of the Sponsor.

12.5 Data Quality

Data collection tools will undergo appropriate validation to ensure that data are collected accurately and completely. Datasets provided for analysis will be participant to quality control processes to ensure analysed data is a true reflection of the source data.
Study data will be managed in compliance with local data management SOPs (including the overarching SOP OVC007 Data and Database Management). If additional, study-specific information is required, an approved Data Management Plan will be implemented.

13. QUALITY CONTROL AND QUALITY ASSURANCE PROCEDURES

13.1 Investigator procedures

Approved site-specific standard operating procedures (SOPs) will be used at all clinical and laboratory sites.

13.2 Monitoring

Regular monitoring will be performed according to GCP by the monitor. Following written SOPs, the monitor will verify that the study is conducted and data are generated, documented and reported in compliance with the protocol, GCP and the applicable regulatory requirements. The site will provide direct access to all study related source data/documents and reports for the purpose of monitoring and auditing by the Sponsor and inspection by local and regulatory authorities.

13.3 Protocol deviation

Any deviations from the protocol will be documented in a protocol deviation form and filed in the trial master file. Each deviation will be assessed as to its impact on volunteer safety and study conduct. Significant protocol deviations will be listed in the end of study report.

13.4 Audit & inspection

The QA manager conducts systems based internal audits to check that trials are being conducted according to local procedures and in compliance with GCP and applicable regulations.

The Sponsor, trial sites, and ethical committee(s) may carry out audit to ensure compliance with the protocol, GCP and appropriate regulations.

14. SERIOUS BREACHES

A serious breach is defined as “A breach of GCP or the study protocol which is likely to affect to a significant degree:

- the safety or physical or mental integrity of the participants of the study
- the scientific value of the study”

In the event that a serious breach is suspected, the Sponsor will contact the REC within 7 days within becoming aware of the breach of GCP.

15. ETHICS AND REGULATORY CONSIDERATIONS

15.1 Declaration of Helsinki

The Investigators will ensure that this study is conducted according to the principles of the current revision of the Declaration of Helsinki.

15.2 Guidelines for Good Clinical Practice

The Investigator will ensure that this study is conducted in accordance with relevant regulations and with Good Clinical Practice.

15.3 Ethical Approvals

The protocol, informed consent form, participant information sheet and any proposed advertising material will be submitted to an appropriate Research Ethics Committee (REC), HRA and host institution(s) for written approval.

No substantial amendments to this protocol will be made without consultation with, and agreement of, the sponsor. Any substantial amendments that appear necessary during the course of the study must be discussed by the investigator and sponsor concurrently. If agreement is reached concerning the need for an amendment, it will be produced in writing by the chief investigator (or delegate) and will be made a formal part of the protocol following ethical approval.

The Investigator is responsible for ensuring that changes to an approved study, during the period for which ethical committee(s)' approval has already been given, are not initiated without ethical committee(s)' review and approval except to eliminate apparent immediate hazards to the participant (i.e as an Urgent Safety Measure).

The Investigators will notify deviations from the protocol or SAEs occurring at the site to the Sponsor and will notify the REC of these in accordance with local procedures.

15.4 Volunteer Confidentiality

All data will be de-identified: volunteer data will be identified by a unique study number in the CRF and database. A separate confidential file containing identifiable information will be stored in a secured location in accordance with the Data Protection Act 2018. Only the sponsor representative, investigators, the clinical monitor and the REC will have access to the records. Photographs taken of challenge sites (if required, with the volunteer's written, informed consent) will not include the volunteer's face and will be identified by the date, trial code and participant's unique identifier. Once developed, photographs will be stored as confidential records, as above. This material may be shown to other professional staff, used for educational purposes, or included in a scientific publication.

An exception will be reporting of SARS-CoV-2 test results, as required by law. All results (either positive or negative) and personal data (including volunteer name, contact details, and postcode) may be shared in a secure manner with UK HSA.

For new uses of BCG that may be licensed, we may store research data securely at the University of Oxford for up to 15 years after the end of the study

16. FINANCING AND INSURANCE

16.1 Financing

This study will be funded by a research grant from the NIH/NIAD. Funds for the NIH/NIAD grant have been awarded for the whole duration of the study and will be released on year per year basis, upon review of annual results.

16.2 Insurance

The University has a specialist insurance policy in place which would operate in the event of any participant suffering harm as a result of their involvement in the research (Newline Underwriting Management Ltd, at Lloyd's of London).

16.3 Compensation

Volunteers will be compensated for their time, inconvenience and their travel expenses. The total amount compensated will be approximately £705-750 depending on the exact number of visits, and whether any repeat or additional visits are necessary.

Should the volunteer decide to withdraw from the study before it is completed, payment will be pro rata

17. PUBLICATION POLICY

The Investigators will be involved in reviewing drafts of the manuscripts, abstracts, press releases and any other publications arising from the study. Data from the study may also be used as part of a thesis for a PhD or MD. When the study is complete, a manuscript describing the primary study results will be written and published in a peer-reviewed, open access journal. International guidelines will be followed regarding authorship. There may also be secondary publications on more exploratory results.

18. REFERENCES

1. Dye C, Scheele S, Dolin P, Pathania V, Raviglione MC. Consensus statement. Global burden of tuberculosis: estimated incidence, prevalence, and mortality by country. WHO Global Surveillance and Monitoring Project. JAMA : the journal of the American Medical Association. 1999;282(7):677-86.
2. WHO. Global tuberculosis report 2019. Geneva http://www.who.int/tb/publications/global_report/en/ (Accessed 21 November 2019): World Health Organization; 2019. Contract No.: CC BY-NCSA 3.0 IGO.
3. Corbett EL, Marston B, Churchyard GJ, De Cock KM. Tuberculosis in sub-Saharan Africa: opportunities, challenges, and change in the era of antiretroviral treatment. Lancet (London, England). 2006;367(9514):926-37.
4. Colditz GA, Brewer TF, Berkey CS, Wilson ME, Burdick E, Fineberg HV, et al. Efficacy of BCG vaccine in the prevention of tuberculosis. Meta-analysis of the published literature. Jama. 1994;271(9):698-702.
5. Fine PE. The BCG story: lessons from the past and implications for the future. Rev Infect Dis. 1989;11 Suppl 2:S353-9.
6. Trunz BB, Fine P, Dye C. Effect of BCG vaccination on childhood tuberculous meningitis and miliary tuberculosis worldwide: a meta-analysis and assessment of cost-effectiveness. Lancet (London, England). 2006;367(9517):1173-80.
7. Rodrigues LC, Diwan VK, Wheeler JG. Protective effect of BCG against tuberculous meningitis and miliary tuberculosis: a meta-analysis. International journal of epidemiology. 1993;22(6):1154-8.
8. Tameris MD, Hatherill M, Landry BS, Scriba TJ, Snowden MA, Lockhart S, et al. Safety and efficacy of MVA85A, a new tuberculosis vaccine, in infants previously vaccinated with BCG: a randomised, placebo-controlled phase 2b trial. Lancet (London, England). 2013;381(9871):1021-8.
9. Ndiaye BP, Thienemann F, Ota M, Landry BS, Camara M, Dieye S, et al. Safety, immunogenicity, and efficacy of the candidate tuberculosis vaccine MVA85A in healthy adults infected with HIV-1: a randomised, placebo-controlled, phase 2 trial. The Lancet Respiratory medicine. 2015;3(3):190-200.
10. Scriba TJ, Coussens AK, Fletcher HA. Human Immunology of Tuberculosis. Microbiology spectrum. 2016;4(4).
11. Brighenti S, Andersson J. Local immune responses in human tuberculosis: learning from the site of infection. The Journal of infectious diseases. 2012;205 Suppl 2:S316-24.
12. Flynn JL, Chan J, Triebold KJ, Dalton DK, Stewart TA, Bloom BR. An essential role for interferon gamma in resistance to Mycobacterium tuberculosis infection. J Exp Med. 1993;178(6):2249-54.
13. Newport MJ, Huxley CM, Huston S, Hawrylowicz CM, Oostra BA, Williamson R, et al. A mutation in the interferon-gamma-receptor gene and susceptibility to mycobacterial infection. The New England journal of medicine. 1996;335(26):1941-9.
14. Flynn JL, Goldstein MM, Chan J, Triebold KJ, Pfeffer K, Lowenstein CJ, et al. Tumor necrosis factor-alpha is required in the protective immune response against Mycobacterium tuberculosis in mice. Immunity. 1995;2(6):561-72.
15. Abramovitch, R. B. Mycobacterium tuberculosis reporter strains as tools for drug discovery and development. IUBMB Life. 2018; 70, 818–825
16. McShane H, Williams A. A review of preclinical animal models utilised for TB vaccine evaluation in the context of recent human efficacy data. Tuberculosis (Edinb). 2014;94(2):105–110.
17. Minassian AM, Ronan EO, Poyntz H, et al. Preclinical development of an in vivo BCG challenge model for testing candidate TB vaccine efficacy. PLoS One. 2011; 6(5): e19840
18. Harris SA, White A, Stockdale L, et al.: Development of a non-human primate BCG infection model for the evaluation of candidate tuberculosis vaccines. *Tuberculosis (Edinb).* 2018; 108: 99–105.
19. Villarreal-Ramos B , Berg S, Chamberlain L, McShane H, Hewinson RG, Clifford D, Vordermeier M. Development of a BCG challenge model for the testing of vaccine candidates against tuberculosis in cattle. Vaccine. 2014 Sep 29; 32(43):5645-9

20. Harris SA, Meyer J, Satti I, Marsay L, Poulton ID, Tanner R, et al. Evaluation of a human BCG challenge model to assess antimycobacterial immunity induced by BCG and a candidate tuberculosis vaccine, MVA85A, alone and in combination. The Journal of infectious diseases. 2014;209(8):1259-68.
21. Minassian AM, Satti I, Poulton ID, et al.: A human challenge model for Mycobacterium tuberculosis using Mycobacterium bovis bacille CalmetteGuerin. J Infect Dis. 2012;205(7):1035-42
22. Minhinnick A, Harris S, Wilkie M, et al. Optimization of a human bacille calmette-guérin challenge model: a tool to evaluate antimycobacterial immunity. J Infect Dis. 2016;213(5):824–830
23. Hart PD, Sutherland I. BCG and Vole Bacillus Vaccines in the Prevention of Tuberculosis in Adolescence and Early Adult Life. BMJ. 1977;2:293–5.
24. Matsumiya, M., et al., Gene expression and cytokine profile correlate with mycobacterial growth in a human BCG challenge model. J Infect Dis, 2015. 211(9): p. 1499-509.
25. Zhou QT, Leung SS, Tang P, Parumasivam T, Loh ZH, Chan HK. Inhaled formulations and pulmonary drug delivery systems for respiratory infections. Advanced drug delivery reviews. 2015;85:83-99.
26. Rau JL. The inhalation of drugs: advantages and problems. Respiratory care. 2005;50(3):367-82
27. Cusumano CL, Jernigan JA, Waldman RH. Aerosolized BCG (Tice strain) treatment of bronchogenic carcinoma: phase I study. J Natl Cancer Inst. 1975;55(2):275-9.
28. Garner FB, Meyer CA, White DS, Lipton A. Aerosol BCG treatment of carcinoma metastatic to the lung: a phase I study. Cancer. 1975;35(4):1088-94.
29. Satti I, Meyer J, Harris SA, Manjaly Thomas ZR, Griffiths K, Antrobus RD, et al. Safety and immunogenicity of a candidate tuberculosis vaccine MVA85A delivered by aerosol in BCG-vaccinated healthy adults: a phase 1, double-blind, randomised controlled trial. The Lancet Infectious diseases. 2014;14(10):939-46.
30. WHO. IVR strategic plan 2010 - 2020. http://whqlibdoc.who.int/hq/2010/WHO_IVB_10.02_eng.pdf?ua=1 (accessed 21 November 2019) 2010.
31. Rosenthal SR, McEnery JT, Raisys N. Aerogenic BCG vaccination against tuberculosis in animal and human subjects. J Asthma Res. 1968;5(4):309-23.
32. Nuermberger EL, Yoshimatsu T, Tyagi S, Bishai WR, Grosset JH. Paucibacillary tuberculosis in mice after prior aerosol immunization with Mycobacterium bovis BCG. Infect Immun. 2004;72(2):1065-71.
33. White AD, Sarfas C, West K, Sibley LS, Wareham AS, Clark S, et al. Evaluation of the Immunogenicity of Mycobacterium bovis BCG Delivered by Aerosol to the Lungs of Macaques. Clinical and vaccine immunology : CVI. 2015;22(9):992-1003.
34. Vaccines A. BCG Danish 1331 AJ Vaccines Summary of Product Characteristics. 2018
35. Kariyawasam, H.H., et al., Safety and tolerability of three consecutive bronchoscopies after allergen challenge in volunteers with mild asthma. Thorax, 2007. 62(6): p. 557-8. 31.
36. Elston, W.J., et al., Safety of research bronchoscopy, biopsy and bronchoalveolar lavage in asthma. Eur Respir J, 2004. 24(3): p. 375-7. 32.
37. Busse, W.W., et al., Investigative bronchoprovocation and bronchoscopy in airway diseases. Am J Respir Crit Care Med, 2005. 172(7): p. 807-16.
38. Stahl DL, Richard KM, Papadimos TJ. Complications of bronchoscopy: A concise synopsis. Int J Crit Illn Inj Sci. 2015;5(3):189–195.
39. OMRON. OMRON Microair website http://www.omron-healthcare.com/eu/en/our-products/respiratory-therapy/microair-u22 (accessed 21 November 2019). 2019.
40. Fentanyl www.medicines.org.uk/emc/product/2852/smpc (accessed 21 November 2019).
41. Midazolam www.medicines.org.uk/emc/product/5668/smpc (accessed 21 November 2019).
42. Lignocaine www.medicines.org.uk/emc/product/4781/smpc (accessed 21 November 2019).
43. Lurie N, Saville M, Hatchett R, Halton J. Developing Covid-19 Vaccines at Pandemic Speed. New England Journal of Medicine. 2020;382(21):1969-73
44. Public Health England, gov.uk coronavirus website https://coronavirus.data.gov.uk/ (accessed 20 April 2021).

45. Baldwin DR, WS Lim, R Rintoul, N Navani, L Fuller, I Woolhouse, M Evison. R Booton, S Janes, R Thakrar, M Callister, M Munavvar. Recommendations for day case bronchoscopy services during the COVID-19 pandemic. Version 2: Services during the restoration and recovery COVID-19 endemic phase: British Thoracic Society;2020.
46. Hegewald MJ, Jensen RL, Teeter JG, Wise RA, Riese RJ, England RD, et al. Long-term intersession variability for single-breath diffusing capacity. Respiration. 2012;84(5):377-84.
47. Davids, M., et al. A Human Lung Challenge Model to Evaluate the Safety and Immunogenicity of PPD and Live Bacillus Calmette-Guérin. Am J Respir Crit Care Med,2020. 201(10): 1277-1291.
48. Weiszhar Z & Horvath I. Induced sputum analysis: step by step. Breathe, 2013 9(4): 300-306
49. OMRON. OMRON ultrasonic nebuliser NE-U780 Instruction manual webpage: https://www.omronhealthcare-ap.com/Content/uploads/products/563bac0e447d4d33b8ae3b1e5baa5609.pdf (accessed 18th November 2020)
50. Williams CM, Abdulwhhab M, Birring SS, De Kock E, Garton NJ, Townsend E, Pareek M, Al-Taie A, Pan J, Ganatra R, Stoltz AC, Haldar P, Barer MR. Exhaled Mycobacterium tuberculosis output and detection of subclinical disease by face-mask sampling: prospective observational studies. Lancet Infect Dis. 2020 May;20(5):607-617.

19. Appendices

Appendix A: Covid 19 study specific self-isolation guidance (Risk assessment)

In this section we describe study specific self-isolation guidance based on either government guidance or expert opinion in combination with reviewed data on side effects seen post challenge and post bronchoscopy from participants enrolled into TB041 or TB043.

With regard to fever post-challenge:

- UK Health Security Agency (UKHSA) have written **specific guidance** for fever following routine vaccinations recognising that fever is an expected side effect post vaccination.
- This details*: "vaccines given may cause a fever which usually resolves within 48 hours (or 6 to 11 days following MMR). This is a common, expected reaction and isolation is not required, unless COVID-19 is suspected."* And they also clarify that *"This advice applies to recently vaccinated people of all ages."*
- The link for this guidance can be found at: https://www.nice.org.uk/media/default/about/covid-19/specialty-guides/maintaining-immunisation-programmes.pdf
- Having liaised with local immunisation teams for the COVID-19 vaccine trial this guidance is being interpreted as no need for self-isolation or COVID-19 testing in the immediate post vaccination period **when isolated fever** is an expected side effect.
- Fever is a recognised side effect of BCG as listed in the British National Formulary.
- We know from TB041/TB043 – 5.6% of participants had a temperature of >37.8 after either aerosolised or intradermal BCG.
- Prior to their challenge visit we will ensure that participants have no symptoms concerning for COVID-19 infection and we will also perform a swab to identify asymptomatic/pre-symptomatic COVID-19 disease.
- Given that we have already looked for COVID-19 infection it can be safely assumed that an isolated fever in the 48 hours after challenge is highly likely to be an expected side effect. We can therefore advise no need for isolation/ testing in the case of isolated fever lasting less than 48 hours post challenge.

With regard to fever post bronchoscopy:

- Bronchoscopy procedure has a 10% risk of fever (quoted in NHS consent). In TB041/TB043 9.7% of participants had a documented fever in the 48 hours post bronchoscopy.
- Given the frequency of fever and the fact we have already tested for COVID-19 infection immediately pre-bronchoscopy, we can be confident that a short lived fever is an expected side effect. We can therefore advise no need for isolation/testing if the fever occurs within the 48 hours of the procedure and lasts less than 48 hours duration.
- This has also been designed to match the advice the OUH team are giving other research bronchoscopy patients as discussed with respiratory consultants (Dr Tim Hinks & Alistair Moore, personal communication), who have compiled their guidance in discussion with the British Thoracic Society guideline group (45).

Cough post challenge & bronchoscopy:

- Cough is extremely common post challenge with aerosolised BCG and is almost universally reported post bronchoscopy.

- Cough post aerosol inhalation was seen in 43% of volunteers in TB041/TB043, with the majority occurring before day 5 (6.8% had a cough starting on or after day 5).
- Cough post bronchoscopy was recorded in 86% of volunteers
- The mean duration of post bronchoscopy cough was 5 days in TB041/TB043

- Prior to challenge & bronchoscopy, all participants will be tested for COVID-19 infection and in view of the high frequency of cough after both procedures we can be reassured that cough in the first 5 days is likely an expected side effect rather than new covid-19 infection.
- After 5 days cough can still occur in both groups, but the elapsed time period increases the risk of having subsequently been infected with COVID-19 and therefore we would advise isolation and testing after this time period.
- This advice is again in line with the OUH bronchoscopy advice being given.

Throughout the study, clinical discretion will be used and any symptoms felt to be unexpected or concerning for COVID-19 infection will be treated in line with UKHSA guidance. However, without study specific isolation advice, given the frequency of cough and fever post challenge and bronchoscopy we would almost certainly be asking the majority of our participants (and household contacts) to self-isolate if they want to partake in the trial. This will almost certainly reduce the number of volunteers willing to participate in our study and furthermore is not in line with current UKHSA vaccination advice or local/national research bronchoscopy advice.
