# Supplementary material for: A dose escalation study to evaluate the safety of an aerosol BCG infection in previously BCG-vaccinated healthy human UK adults
Source: Front Immunol. 2024 Nov 14;15:1427371. doi: 10.3389/fimmu.2024.1427371 (PMC11602284; doi:10.3389/fimmu.2024.1427371)
Supplement: Supplementary file 2 [file Table1.pdf]

Table 1. – Unsolicited and Solicited AEs first reported after the first 14 days of Aerosol BCG challenge coded according the MEDRRA preferred term and separated by maximum reported grade and study group.

	Participants reporting AE, n (%)				
	Group 1	Group 2	Group 3	Group 4	Total
AE's assessed as "possibly", "probably" or "definitely" related to BCG administration					
**General disorders and administration site conditions**					
Night sweat					
Grade 2	-	-	-	1 (33%)	1 (8%)
Shivers					
Grade 1	-	-	-	1 (33%)	1 (8%)
**Musculoskeletal and connective tissue disorders**					
Musculoskeletal discomfort					
Grade 1	-	-	-	1 (33%)	1 (8%)
Myalgia					
Grade 1	1 (33%)	-	-	-	1 (8%)
**Gastrointestinal disorders**					
Toothache					
Grade 1	-	1 (33%)	-	-	1 (8%)
Mouth ulceration					
Grade 1	-	1 (33%)	-	-	1 (8%)
Diarrhoea					
Grade 1	-	-	-	1 (33%)	1 (8%)
Abdominal pain	-	-	-	-	
Grade 1	-	-	-	1 (33%)	1 (8%)
**Respiratory, thoracic and mediastinal disorders**					
Rhinitis					
Grade 1	-	1 (33%)	-	-	1 (8%)
