# Supplementary material for: A dose escalation study to evaluate the safety of an aerosol BCG infection in previously BCG-vaccinated healthy human UK adults
Source: Front Immunol. 2024 Nov 14;15:1427371. doi: 10.3389/fimmu.2024.1427371 (PMC11602284; doi:10.3389/fimmu.2024.1427371)
Supplement: Supplementary file 3 [file Table2.pdf]

Table 2. Median duration of Solicited AEs reported within 72 hours of bronchoscopy.
Grading range 1-3, AE presented here started within the first 72 hours of e-diary self-reporting. Group 1 - 1 x 10^4 cfu; Group 2 - 1 x 10^5 cfu; Group 3 - 1 x 10^6 cfu; Group 4 - 1 x 10^7 cfu BCG SSI.

	Number of volunteers (proportion, max grade)					
**Solicited AE**	**Group 1 n=3**	**Group 2 n=3**	**Group 3 n=3**	**Group 4 n=3**	**Total n=12**	**Median duration of AE in days (range)**
Temperature	0	0	1 (0.33, 3)	1 (0.33, 1)	2	1.5 (1 – 2)
Arthralgia	0	0	1 (0.33, 2)	1 (0.33, 1)	2	2.5 (1 – 4)
Myalgia	1 (0.33, 1)	1 (0.33, 1)	2 (0.66, 2)	0	4	1 (1 – 5)
Feverishness	2 (0.66, 1)	1 (0.33, 1)	1 (0.33, 2)	1 (0.33, 1)	5	1 (1 – 6)
Headache	1 (0.33, 1)	1 (0.33, 2)	1 (0.33, 1)	0	3	2 (2 – 3)
Fatigue	3 (0.33, 1)	2 (0.66, 2)	2 (0.66, 1)	2 (0.66, 1)	9	1 (1 – 4)
Nausea	0	2 (0.66, 1)	1 (0.33, 1)	0	3	1 (1 – 1)
Malaise	0	2 (0.66, 1)	2 (0.66, 2)	1 (0.33, 1)	5	2 (1 – 2)
Cough	2 (0.66, 1)	3 (1, 1)	3 (1, 2)	3 (1, 1)	11	3 (1 – 148)
Sore Throat	2 (0.66, 2)	3 (1, 2)	3 (1, 2)	3 (1, 1)	11	2 (1 – 5)
Tickly Throat	1 (0.33, 1)	3 (1, 2)	1 (0.33, 1)	1 (0.33, 1)	6	2.5 (2 – 12)
Wheeze	2 (0.66, 1)	0	0	1 (0.33, 1)	3	2 (1 – 4)
SOB	0	2 (0.66, 2)	2 (0.66, 1)	0	4	4 (1 – 2)
Cough Phlegm	1 (0.33, 1)	1 (0.33, 1)	0	0	2	1.5 (1 – 2)
Cough Blood	0	0	0	0	0	– –
Chest Tightness	1 (0.33, 1)	0	0	0	1	1 –
Chest Pain	0	0	0	0	0	– –
