# Supplementary material for: A dose escalation study to evaluate the safety of an aerosol BCG infection in previously BCG-vaccinated healthy human UK adults
Source: Front Immunol. 2024 Nov 14;15:1427371. doi: 10.3389/fimmu.2024.1427371 (PMC11602284; doi:10.3389/fimmu.2024.1427371)

Figure 1. Heatmap for volunteer 014 of solicited systemic and respiratory AE's reported in 28 day E-dairy after aerosol BCG challenge with 1 x 10^7 cfu BCG SSI separated by grade.

Nausea, Cough with blood, and Chest pain not shown as grade 0 throughout duration of e-dairy. Aerosol BCG challenge performed on morning of Day 0 (green box), Bronchoscopy occurred on morning of Day 14 (purple box).

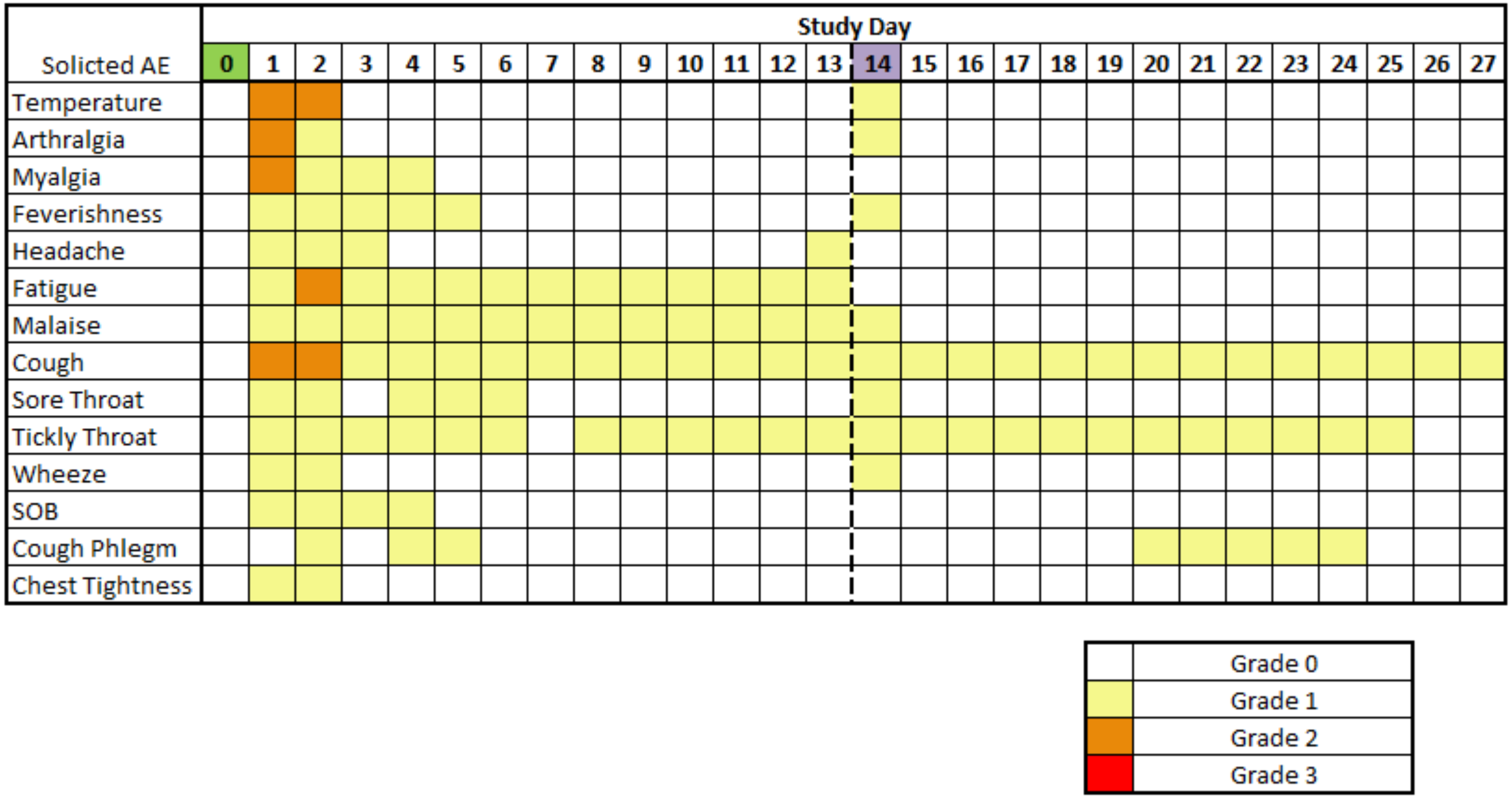

Supplement: Supplementary file 4 [file Image1.pdf]
